# Emergence and Pathogenicity of Highly Virulent *Cryptococcus gattii* Genotypes in the Northwest United States

**DOI:** 10.1371/journal.ppat.1000850

**Published:** 2010-04-22

**Authors:** Edmond J. Byrnes, Wenjun Li, Yonathan Lewit, Hansong Ma, Kerstin Voelz, Ping Ren, Dee A. Carter, Vishnu Chaturvedi, Robert J. Bildfell, Robin C. May, Joseph Heitman

**Affiliations:** 1 Department of Molecular Genetics and Microbiology, Duke University Medical Center, Durham, North Carolina, United States of America; 2 Department of Molecular Pathobiology, School of Biosciences, University of Birmingham, Birmingham, United Kingdom; 3 Mycology Laboratory, Wadsworth Center, Albany, New York, United States of America; 4 Department of Molecular and Microbial Biosciences, The University of Sydney, Sydney, New South Wales, Australia; 5 Department of Biomedical Sciences, Oregon State University, Corvallis, Oregon, United States of America; University of Melbourne, Australia

## Abstract

*Cryptococcus gattii* causes life-threatening disease in otherwise healthy hosts and to a lesser extent in immunocompromised hosts. The highest incidence for this disease is on Vancouver Island, Canada, where an outbreak is expanding into neighboring regions including mainland British Columbia and the United States. This outbreak is caused predominantly by *C. gattii* molecular type VGII, specifically VGIIa/major. In addition, a novel genotype, VGIIc, has emerged in Oregon and is now a major source of illness in the region. Through molecular epidemiology and population analysis of MLST and VNTR markers, we show that the VGIIc group is clonal and hypothesize it arose recently. The VGIIa/IIc outbreak lineages are sexually fertile and studies support ongoing recombination in the global VGII population. This illustrates two hallmarks of emerging outbreaks: high clonality and the emergence of novel genotypes via recombination. In macrophage and murine infections, the novel VGIIc genotype and VGIIa/major isolates from the United States are highly virulent compared to similar non-outbreak VGIIa/major-related isolates. Combined MLST-VNTR analysis distinguishes clonal expansion of the VGIIa/major outbreak genotype from related but distinguishable less-virulent genotypes isolated from other geographic regions. Our evidence documents emerging hypervirulent genotypes in the United States that may expand further and provides insight into the possible molecular and geographic origins of the outbreak.

## Author Summary

Emerging and reemerging infectious diseases are increasing worldwide and represent a major public health concern. One class of emerging human and animal diseases is caused by fungi. In this study, we examine the expansion on an outbreak of a fungus, *Cryptococcus gattii*, in the Pacific Northwest of the United States. This fungus has been considered a tropical fungus, but emerged to cause an outbreak in the temperate climes of Vancouver Island in 1999 that is now causing disease in humans and animals in the United States. In this study we applied a method of sequence bar-coding to determine how the isolates causing disease are related to those on Vancouver Island and elsewhere globally. We also expand on the discovery of a new pathogenic strain recently identified only in Oregon and show that it is highly virulent in immune cell and whole animal virulence experiments. These studies extend our understanding of how diseases emerge in new climates and how they adapt to these regions to cause disease. Our findings suggest further expansion into neighboring regions is likely to occur and aim to increase disease awareness in the region.

## Introduction

Newly emerging and reemerging diseases have become a major focus of infectious disease research in the 21^st^ century. Reemerging diseases are classified as those that have been previously documented, but are now rapidly increasing in incidence, geographic range, or both [1]. Emerging disease events have been occurring at higher than average rates in the United States due to several factors such as wildlife diversity, environmental change, international travel, and increases in host susceptibility [2], [3]. An additional factor contributing to increases in morbidity and mortality for many infectious diseases involves genetic recombination events or gene/pathogenicity island acquisitions. These events can occur via either horizontal gene transfer or conjugation/introgression, leading to novel pathogenic genotypes. This form of virulence evolution has been well characterized in bacterial, viral, fungal, and parasitic human diseases [4], [5], [6], [7], [8], [9]. The ability to cause damage to mammalian hosts is a common theme among all microbial pathogens, making it a key aspect of host-pathogen studies [10].

In the genomic era, it is now possible to combine conventional epidemiological approaches with newly developed molecular typing techniques to gain insight into the emergence and molecular epidemiology of pathogens. These approaches can improve understanding of population dynamics during an outbreak, and may lead to novel methods for the rapid identification, treatment, and diagnosis of emerging infections [11]. In addition, molecular typing serves as an initial approach to classify isolates into distinct genotypes for analysis. Further investigations may include the examination of virulence and phenotypic traits that may be common or distinct between genotypes [6], [12], [13]. Gaining insights into the molecular epidemiology and virulence of newly emerging diseases has considerable potential for the rapid assessment and management of newly emerging infections.

Over the past decade, *Cryptococcus gattii* has emerged as a primary pathogen in northwestern North America, including both Canada and the United States [6], [13], [14], [15], [16], [17], [18]. In the past, *C. gattii* has often been associated with *Eucalyptus* trees in tropical and subtropical climates, causing disease in immunocompetent hosts at low incidences [19], [20], [21]. *C. gattii* is distinct from its sibling species *Cryptococcus neoformans* [22], which more commonly infects immunosuppressed hosts and infects almost one million people annually with over 620,000 attributable mortalities [23], [24], [25]. *C. gattii* can be classified into four discrete molecular types (VGI-VGIV), which represent cryptic species as no nuclear allelic exchange between groups has been observed [6]. This molecular classification is significant because VGII is responsible for approximately 95% of the Pacific Northwest infections in Canada and the United States [12], [15]. The appearance of *C. gattii* in North America is alarming because this is the first major emergence in a temperate climate, indicating a possible expansion in the endemic ecology of this pathogen [26], [27].

Several significant questions persist regarding the outbreak and its expansion within the United States. As the global collection of *C. gattii* isolates expands, the molecular epidemiology of the species has become increasingly informative, particularly through multilocus sequence typing (MLST), which allows data to be readily compared between groups within the research community [6], [15], [28], [29], [30]. The increase in global and regional isolates that have been typed at the molecular level allows detailed analysis of *C. gattii*. The analysis of both conserved coding regions, and diverse noncoding regions provides insight into the genotypes responsible for the outbreak. A major finding in this study is a level of underlying diversity within the VGIIa/major genotype in the region of expansion and other geographic locales.

Prior studies documented that the *C. gattii* VGIIa/major genotype isolates from Vancouver Island are highly virulent in experimental murine infection assays [6]. Here we expanded this analysis to examine clinical VGIIa genotype isolates from Vancouver Island, the United States, and Brazil, in addition to an environmental VGIIa isolate from California. Our findings are consistent with recent macrophage intracellular proliferation studies, demonstrating that United States isolates from the recent Pacific NW outbreak exhibit high virulence [31]. The enhanced virulence of isolates from the outbreak region, when compared with those from other regions, suggests that the genotypes circulating in the Pacific NW are inherently increased in their predilection to cause disease in mammalian hosts.

In addition to the detailed examination of the VGIIa/major genotype clade, we report that the novel VGIIc genotype is highly virulent in a murine inhalation model. Moreover, the VGIIc genotype was found to have high intracellular proliferation rates in macrophages and a significantly increased percentage of mitochondria with tubular morphology after macrophage exposure, and thus VGIIc isolates share virulence attributes with the VGIIa/major genotype isolates from the Vancouver Island outbreak. These results extend the molecular and phenotypic understanding of the recently discovered VGIIc/novel genotype and help shed light into its possible geographic and molecular origins.

These studies provide insights into both the evolutionary history and virulence characteristics of this unique and increasingly fatal fungal outbreak in the temperate climate of the North American Pacific Northwest and highlight the importance of a collaborative interdisciplinary approach to the analysis of emerging pathogens. Application of these approaches may increase awareness of disease risks in the expansion zone, lead to more rapid diagnoses and, as a result, accelerate the implementation of appropriate therapy.

## Materials and Methods

### Isolate Identification

Human and veterinary cases of confirmed or suspected *C. gattii* infections in the states of Washington and Oregon were identified by referring physicians and veterinarians, and subsequently isolates were purified and examined. Melanin production was assayed by growth and dark pigmentation on Staib's niger seed medium, and urease activity was detected by growth and alkaline pH change on Christensen's agar. These tests established that isolates were *Cryptococcus* (*C. neoformans* or *C. gattii*). Isolates were concomitantly examined for resistance to canavanine and utilization of glycine on L-canavanine, glycine, 2-bromothymol blue (CGB) agar. Growth on CGB agar indicates that isolates are canavanine resistant, and able to use glycine as a sole carbon source, triggering a bromothymol blue color reaction indicative of *C. gattii*, whereas *C. neoformans* is sensitive to canavanine, and cannot use glycine as a sole carbon source, resulting in no growth or coloration in this selective indicator medium. All CGB positive isolates were then grown under rich culture conditions prior to storage at −80°C in 25% glycerol and genomic DNA extraction. For genomic DNA isolation, a modified protocol of the MasterPure Yeast DNA purification kit from Epicentre Biotechnologies was used. Briefly, 500 µl of glass beads (425–600 nm) were added into the combination of cells and 300 µl cell lysis solution. The rest of the method followed the protocol provided by the manufacturer.

### Molecular Epidemiology

For multilocus sequence typing analysis (MLST) [32], each isolate was analyzed with a minimum of eight and in some cases sixteen loci. For each isolate, genomic regions were PCR amplified (Table S1), purified (ExoSAP-IT), and sequenced. All primers used for the analysis were designed specifically to amplify open reading frame (ORF) gene sequence regions including those with non-coding DNA regions to maximize discriminatory power. Sequences from both forward and reverse strands were assembled, and manually edited using Sequencher version 4.8 (Gene Codes Corporations). Based on BLAST analysis of the GenBank database (NCBI), each allele was assigned a corresponding number. GenBank accession numbers with corresponding allele numbers are listed in the supplementary information (Table S2). To determine that the nine VGIIc/novel isolates are clonally related, given the level of diversity in the loci and the number of isolates that have been examined, we applied an equation to measure the probability of a genotype occurring more than once in the dataset [33], [34]. For the variable number of tandem repeat (VNTR) analysis, the Tandem Repeat Finder (TRF) version 4.00 software package was employed for marker development, using the genomic sequence of *C. gattii* isolate R265 (http://www.broadinstitute.org/annotation/genome/cryptococcus_neoformans_b.2/Home.html) [35]. The identified tandem repeat sequences and 400 bp of the flanking region were extracted from the genomic sequence and ranked according to the number of total repeats and the size of repeat units using an in-house Perl script (available upon request). Markers were examined for stability and those with high variability and stability were chosen for the analysis. Sequences were assembled and edited using Sequencher version 4.8 (Gene Codes Corporations) and aligned using the Clustal W web based software package (http://www.ebi.ac.uk/Tools/clustalw2/index.html).

### Mating Conditions

Mating analysis was conducted on V8 media (pH 5). Isolates were incubated at room temperature in the dark for 2–4 weeks in dry conditions. All strains were crossed with the VGIII mating type **a** isolate B4546 and the VGIII mating type α isolate NIH312, both of which are fertile and commonly used for mating studies [36]. Fertility was assessed by microscopic examination for hyphae, fused clamp cells, basidia, and basidiospore formation.

### Clustering and Haplotype Analyses

For each VNTR marker, a sequence type was defined as a sequence exhibiting a unique mutation. Each sequence type was confirmed to be unique by BLAST analysis of the NCBI GenBank database [37]. A concatenated VNTR sequence type (CVST) was defined as unique combinations of sequence types from the VNTR markers. A multiple alignment of the sequences was carried out using Clustal W software [38]. Analysis of the sequences was conducted using the Neighbor-Joining and Maximum Parsimony methods within the MEGA 3.1 software [39]. In addition, the use of the maximum likelihood method (PhyML 3.0) with SH-like approximate likelihood-ratio test and HKY85 substitution model was applied [40], [41]. For this purpose, sequences of the selected VNTR markers were concatenated. We additionally concatenated all of the strain-typing markers including the housekeeping genes used in MLST and VNTR loci for clustering analysis. The haplotype mapping analysis was carried out using TCS software version 1.21 (http://darwin.uvigo.es/software/tcs.html) [42].

### Intracellular Proliferation Rate (IPR) Determination

A proliferation assay was previously developed to monitor the intracellular proliferation rate (IPR) of individual strains for a 64-hour period following phagocytosis [31]. For this assay, J774 macrophage cells were exposed to cryptococcal cells that were opsonized with 18B7 antibody for 2 hr as described previously [43]. Each well was washed with phosphate-buffered saline (PBS) in quadruplicate to remove as many extracellular yeast cells as possible and 1 ml of fresh serum-free DMEM was then added. For time point T = 0, the 1 ml of DMEM was discarded and 200 µl of sterile dH_2_O was added into wells to lyse macrophage cells. After 30 minutes, the intracellular yeast were released and collected. Another 200 µl dH_2_O was added to each well to collect the remaining yeast cells. The intracellular yeast were then mixed with Trypan Blue at a 1∶1 ratio and the live yeast cells were counted. For the subsequent five time points (T = 16 hrs, T = 24 hrs, T = 40 hrs, T = 48 hrs and T = 64 hrs), intracellular cryptococcal cells were collected and independently counted with a hemocytometer. For each strain tested, the time course was repeated at least three independent times, using different batches of macrophages. The IPR value was calculated by dividing the maximum intracellular yeast number by the initial intracellular yeast number at T = 0. We confirmed that Trypan Blue stains 100% of the cryptococcal cells in a heat-killed culture, but only approximately 5% of cells from a standard overnight culture. Compared to a conventional colony counting method, this method was shown to be more sensitive in detecting the clustered yeast population or yeast cells undergoing budding. IPR values were used to assess how consistent the different VGII genotype subgroups were. For this statistical analysis the medians of each population were compared with the non-parametric Mann-Whitney U-test and values of p<0.025, after controlling for multiplicity, and were accepted as statistically significant (http://elegans.swmed.edu/~leon/stats/utest.cgi).

### Mitochondrial Morphology

The mitochondrial morphology assays were conducted in a similar way to those in previous studies, with modifications [31]. *C. gattii* cells, grown overnight at 37°C in DMEM in a 5% CO_2_ incubator without shaking for 24 hr, or isolated from macrophages 24 hr after infection, were harvested, washed with PBS twice and re-suspended in PBS containing the Mito-Tracker Red CMXRos (Invitrogen) at a final concentration of 20 nM. Cells were incubated for 15 min at 37°C. After staining, cells were washed in triplicate and re-suspended in PBS. For each condition, more than 100 yeast cells per replicate for each of the tested strains were chosen randomly and analyzed. For quantifying different mitochondrial morphologies, images were collected using a Zeiss Axiovert 135 TV microscope with a 100× oil immersion Plan-Neofluar objective. Both fluorescence images and phase contrast images were collected simultaneously. Images were captured with identical settings on a QIcam Fast 1394 camera using the QCapture Pro51 version 5.1.1 software. All Images were processed identically in ImageJ and mitochondrial morphologies were analyzed and counted blindly.

Three individual experiments were performed for each condition and the data were tested for normality using the Shapiro-Wilk test. For homogeneity of variances we used the Levene statistic. For statistically significant differences among the mean data we applied a One-Way ANOVA. Multi-comparisons using Tukey Honestly Significant Differences tests were performed to identify statistically significant differences between pairs. A p-value of p<0.05, after controlling for multiplicity, was considered to be statistically significant. Regression analysis was used to measure the correlation between tubular mitochondrial morphology and IPR values; an F-value of P<0.05 was considered to be a significant correlation.

### Murine Virulence Tests and Histopathology

To examine the virulence potential of global VGII isolates, with a specific emphasis on the Pacific NW VGII outbreak genotypes, two independent murine virulence experiments were conducted at two facilities (Duke University Medical Center and the Wadsworth Center). The murine virulence assays at Duke University Medical Center and the Wadsworth Center used a similar protocol to previous *C. gattii* and *C. neoformans* experimental infections [6], [44], [45].

At the Duke University Medical Center Animal Facility, virulence was assessed using female A/Jcr mice (NCI, 18–24 g). Strains were cultured in YPD broth for 18–20 h at 30°C, harvested, washed three times with sterile PBS and counted using a hemocytometer to determine cell concentrations. Inocula for both murine experiments were confirmed by plating on YPD and counting colony-forming units (c.f.u.). Nine to ten A/Jcr mice per strain were anesthetized with pentobarbital and infected via intranasal instillation with 5×10^4^ c.f.u. in 50 µl of sterile 1× PBS. Animals that displayed severe morbidity, based on twice-daily examinations, were euthanized. Time to mortality was evaluated for statistical significance using Kaplan–Meier survival curves within the Prism software package (GraphPad Software), and P values were obtained from a log-rank test. Survival data was plotted for graphical analysis using the Prism software package.

At the Wadsworth center animal facility, all assays were conducted using male BALB/c mice (approximately 6 weeks old, 15–20 g, Charles River Laboratories, Inc.). Strains were grown overnight in YPD broth at 30°C with shaking. The cells were harvested, washed in PBS, and counted using a hemocytometer. Five mice per strain were anesthetized with a mixture of xylazine–ketamine, and allowed to inhale 10^5^ (30 µl) cryptococcal cells per mouse, via intranasal instillation. Mice were given food and water *ad libitum* and monitored twice daily. At the first sign of poor health or discomfort, infected animals were euthanized. Brain and lung tissues from the dead animals were cultured on Niger seed agar for *C. gattii* recovery to confirm infections were due to this pathogen. Time to mortality was evaluated for statistical significance as described above.

Two animals from each strain assayed in the study conducted at Duke University were selected for histopathology analysis either at the time of sacrifice or at the conclusion of the experiment for the more attenuated isolates. For each animal, lung samples were collected and stored in 10% neutral buffered formalin. Samples were paraffin embedded and hematoxylin and eosin (H&E) stained at the Duke University Research Histology Laboratory. After staining and slide preparation, each sample was examined microscopically for analysis of cryptococcal cell burden and immune responses. Images were captured using an Olympus Vanox microscope (Duke PhotoPath, Duke University Medical Center).

### Ethics Statement

The animal studies conducted at the Wadsworth Center were in full compliance with all of the guidelines set forth by the Wadsworth Center Institutional Animal Care and Use Committee (IACUC) and in full compliance with the United States Animal Welfare Act (Public Law 98–198). The Wadsworth Center IACUC approved all of the vertebrate studies. The studies were conducted in facilities accredited by the Association for Assessment and Accreditation of Laboratory Animal Care (AAALAC).

The animal studies at Duke University Medical Center were in full compliance with all of the guidelines of the Duke University Medical Center Institutional Animal Care and Use Committee (IACUC) and in full compliance with the United States Animal Welfare Act (Public Law 98–198). The Duke University Medical Center IACUC approved all of the vertebrate studies. The studies were conducted in Division of Laboratory Animal Resources (DLAR) facilities that are accredited by the Association for Assessment and Accreditation of Laboratory Animal Care (AAALAC).

## Results

### Molecular Analysis of *C. gattii* VGII Outbreak vs. Global Isolates

**Figure 1:**
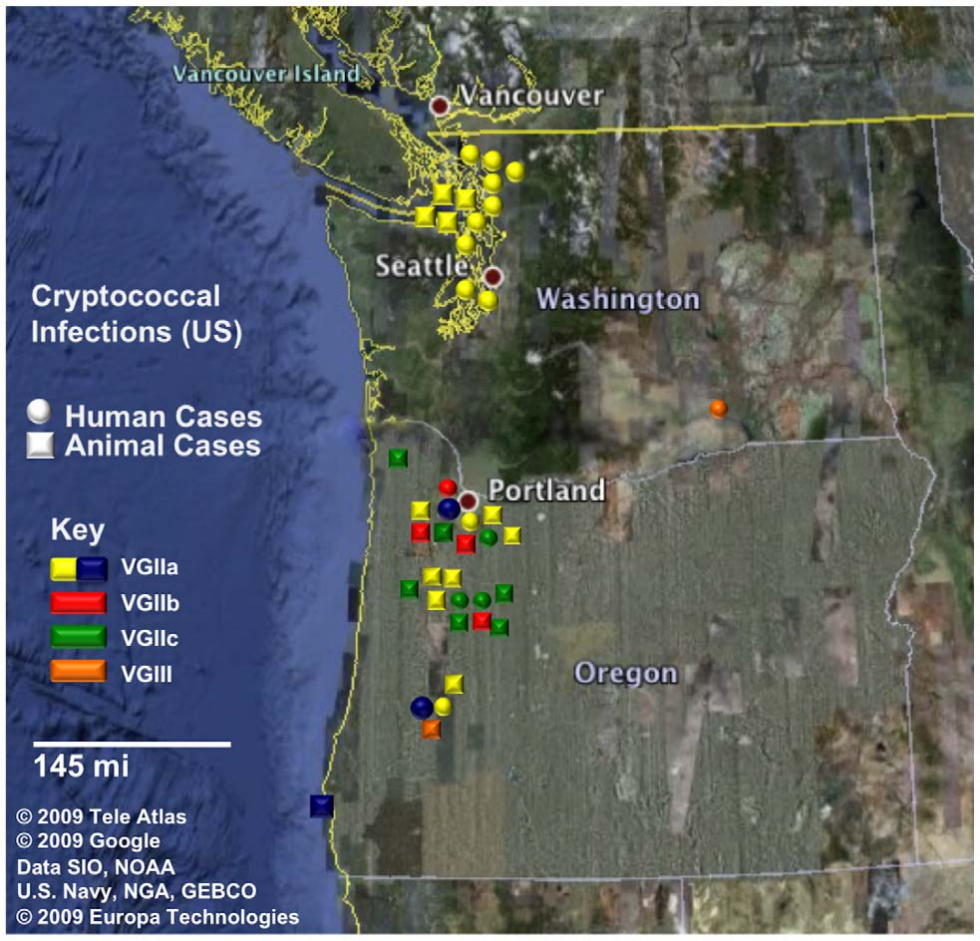
Geographic dispersal of pathogenic *C. gattii* genotypes in the United States. Circles represent human cases and squares represent animal (non-human mammalian) cases. All cases shown have been reported from 2005 to 2009. Isolates are color coded by genotype, in which yellow and blue correspond to VGIIa/major genotype cases (yellow ST1, blue ST30), red corresponds to VGIIb/minor, green corresponds to the novel VGIIc genotype, and orange corresponds to two cases determined to be molecular type VGIII. In total, there were 39 cases (18 human, 21 animal) that have been confirmed by phenotypic and genotypic profiling.

**Figure 2:**
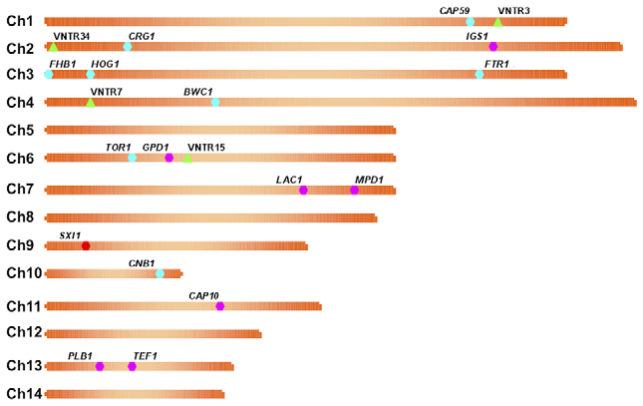
Markers used in the study are dispersed in the genome. A map of each chromosome is represented, illustrating the locations of each marker based on the genomic sequence of the *C. gattii* isolate WM276. MLST markers (n = 16) are indicated on the map by hexagons, with pink denoting the standard set used, blue the expanded set of loci, and red the *MAT* linked locus that is specific to α isolates. Green triangles represent the four VNTR loci that were examined.

**Figure 3:**
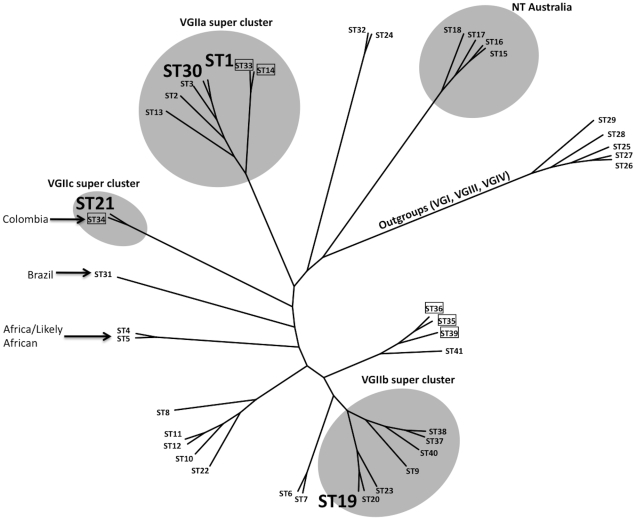
Clustering analysis of global VGII isolates shows high global diversity. This dendrogram, based on seven MLST loci and four VNTR loci, illustrates the global divergence seen in this molecular type. Major clusters are highlighted accordingly to illustrate the placements of the VGIIa/b/c super clusters as well as a unique NT cluster that has been found only in Australia thus far. Sequence types 1, 30, 19, and 20 are enlarged and represent the primary genotypes responsible for the Pacific NW outbreak. Boxed isolates represent those of the **a** mating type and all other sequence types represent the genotypes observed for mating type α isolates. Several genotypes are also combined with geographic information to illustrate the diversity surrounding several sequence types. Isolates from the VGI, VGIII, and VGIV molecular types serve as out-group sequence types.

**Figure 4:**
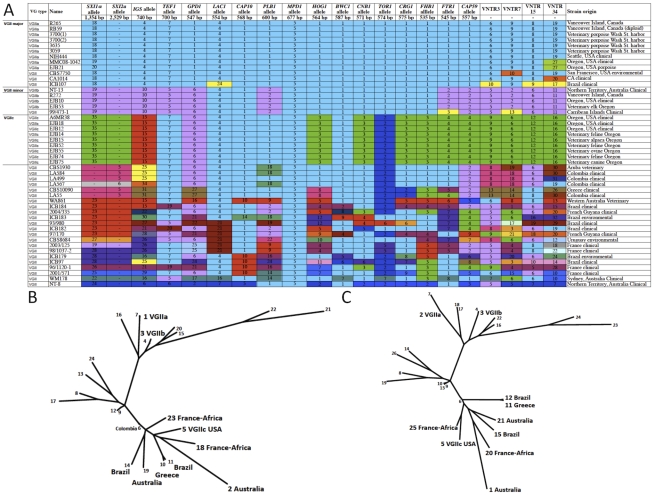
Expanded molecular analysis reveals increased divergence in VGIIc. A) Multilocus sequence typing analysis of 16 loci. Selected isolates from the outbreak in addition to global genotypes were selected for the expanded MLST analysis, including all nine of the VGIIc isolates available. Each unique allele is colored for each marker for visual discrimination, and each number represents a GenBank accession number (Table S2). B) A representation (ML) of the sequence data from panel A, with the exclusion of *MAT* locus linked markers (*SXI1*α/*SXI2* **a**). C) A combination of the sequence data from panel B, with the addition of the four highly variable VNTR markers.

To examine the *C. gattii* outbreak isolates collected from 2005 to 2009 (Figure 1), an in-depth stepwise molecular analysis was applied to each isolate, and the genotypes were compared with other global genotypes. In total, 20 markers were selected for analysis. These markers include both coding and noncoding genomic regions and range in size and allelic diversity (Table 1). Additionally, all of the markers are randomly distributed among the chromosomes in the most recent assembly of the reference *C. gattii* VGI genome, WM276 (Figure 2). Initially, all isolates were sequenced at a total of eight MLST markers, and four variable number of tandem repeats (VNTR) markers (Figure 3, Table 2). Next, global isolates were selected for diversity, and several isolates from each of the primary genotypes in the expansion region were chosen for sequence analysis at eight additional MLST loci, bringing the total number of genetic markers analyzed for these isolates to 20 (Figure 4A). As expected, the MLST markers were less variable and more conserved, while the VNTR markers allowed for higher-resolution differentiation between isolates that appeared identical by MLST analysis. The generated datasets were then concatenated both without and with VNTR data (Figure 4B, Figure 4C).

**Table 1:** Markers used in this study.

Marker	Length (bp)	Chromosome (WM276)	Alleles
*SXI1*α	1,354	9	10
*SXI2* **a**	2,529	N/A*	2
*IGS*	740	2	15
*TEF1*	700	13	5
*GPD1*	547	6	10
*LAC1*	554	7	5
*CAP10*	568	11	4
*PLB1*	600	13	9
*MPD1*	677	7	1
*HOG1*	564	3	11
*BWC1*	587	4	7
*CNB1*	571	10	6
*TOR1*	574	6	5
*CRG1*	575	2	9
*FHB1*	535	3	5
*FTR1*	545	3	7
*CAP59*	557	1	9
VNTR3	334	1	13
VNTR7	270	4	21
VNTR15	364	6	19
VNTR34	526	2	32

* *SXI2* **a** is an idiomorphic allele, and therefore not present in the α mating type isolate WM276.

**Table 2:** Isolates collected from cases within the United States, 2005–2009 (n = 40).

Isolate	Host	Residence	Molecular Type*
T67707	Human	Washington	VGIIa/major
W15209	Human	Washington	VGIIa/major
EJB4	Human	Washington	VGIIa/major
EJB5	Human	Washington	VGIIa/major
EJB6	Human	Washington	VGIIa/major
EJB7	Human	Washington	VGIIa/major
EJB8	Human	Washington	VGIIa/major
EJB9	Human	Washington	VGIIa/major
EJB13	Human	Washington	VGIIa/major
KB11632	Human	Oregon	VGIIa/major
EJB3	Human	Oregon	VGIIa/major
EJB19	Human	Oregon	VGIIa/major
MMC08-1042	Human	Oregon	VGIIa/major
EJB16	Alpaca	Oregon	VGIIa/major
EJB17	Dog	Oregon	VGIIa/major
3700 (1)	Porpoise	Washington	VGIIa/major
3700 (2)	Porpoise	Washington	VGIIa/major
3635	Porpoise	Washington	VGIIa/major
3059	Porpoise	Washington	VGIIa/major
EJB21	Porpoise	Oregon	VGIIa/major
EJB22	Dog	Oregon	VGIIa/major
EJB51	Alpaca	Oregon	VGIIa/major
EJB54	Cat	Oregon	VGIIa/major
EJB77	Dog	Oregon	VGIIa/major
EJB79	Alpaca	Oregon	VGIIa/major
A6MR38	Human	Oregon	VGIIc/novel
EJB12	Human	Oregon	VGIIc/novel
EJB18	Human	Oregon	VGIIc/novel
EJB14	Cat	Oregon	VGIIc/novel
EJB15	Alpaca	Oregon	VGIIc/novel
EJB52	Cat	Oregon	VGIIc/novel
EJB55	Ovine	Oregon	VGIIc/novel
EJB74	Cat	Oregon	VGIIc/novel
EJB75	Dog	Oregon	VGIIc/novel
EJB10	Human	Oregon	VGIIb/minor
MMC08-896	Dog	Oregon	VGIIb/minor
EJB53	Elk	Oregon	VGIIb/minor
EJB76	Cat	Oregon	VGIIb/minor
EJB11	Human	Washington	VGIII
MMC08-897	Cat	Oregon	VGIII

* The Molecular type designation is based on 8-loci MLST analysis.

The combined analysis of the results presented here, and a 30 marker MLST analysis conducted previously [6], [18], reveal several findings of interest in relation to VGII genotypes in the region. From the analysis of 34 markers (30 MLST/4 VNTR), we show that the Vancouver Island VGIIa/major isolates are fully identical at all loci to several recent isolates from Washington and Oregon, as well as a historical clinical isolate (1970's), NIH444, from Seattle. Additionally, the VGIIb/minor isolates from Australia and Vancouver Island are identical at 34 total loci, and also identical to VGIIb/minor isolates from Oregon at 20 loci (16 MLST/4 VNTR). Furthermore, all VGIIc isolates to date are identical across all 20 loci examined (Figure 4A). However, we also are able to discriminate the outbreak VGIIa genotype from an environmental VGIIa isolate from California, CBS7750, and clinical VGIIa isolates CA1014 and ICB107 from California and Brazil, respectively, at one or more MLST/VNTR loci. It is clear from prior studies that the VGIIa/major and VGIIb/minor isolates are clonal lineages [6], [12], [15], [46], and here we confirmed that this is the case for the nine VGIIc/novel isolates, based on 7-loci MLST analysis of the global VGII population (Figure S1) (p<0.0001).

The largest and most comprehensive dataset arose from the combined analysis of seven MLST and four VNTR loci, resulting in a total of 41 sequence types (STs). This dataset was generated from clinical, veterinary, and environmental *C. gattii* isolates (Figure 3, Figure S1, Table S3). From the analysis, it is clear that the VGIIa/b/c clusters are all related to each other, but also distinct. In addition, the data show that the VGIIa/major clade is closely clustered to VGIIc, further validating prior reports that examined a more limited number of loci [13], [47]. In addition, VGIIc (ST21) shares high sequence identity to ST34, represented by a mating type **a** clinical isolate from Colombia, suggesting that the VGIIc genotype may have resulted from **a**-α mating, even though all isolates related to the Pacific NW outbreak are exclusively α mating type. Additionally, Vancouver Island isolates from our collection that had not been fully typed by MLST were sequenced at two loci to determine if any were unrecognized VGIIc isolates (n = 56) (Figure S2). Of these, 51 were found to be VGIIa, five were VGIIb, and none were VGIIc, consistent with previous data from the region. Thus, VGIIc appears to remain exclusive to the United States, specifically Oregon, and has never been reported from Vancouver Island, the mainland of Canada, Washington State, or elsewhere globally.

Within the VGIIa/major cluster, based on the initial MLST analysis of 30 loci, only a single isolate (ICB107) could be distinguished from the other VGIIa isolates, and this was at only one locus [18]. To further investigate this homogeneous population causing the vast majority of the outbreak-related morbidity and mortality, we expanded the molecular analysis to include highly variable regions of the genome. The application of these VNTR markers, in combination with the MLST markers, allowed us to generate five independent STs from within the VGIIa/major genotype and related isolates (Figure 3).

These five sequence types (ST1, ST2, ST3, ST13, ST30) contained a total of 44 isolates (Figure 3, Table S3). The canonical VGIIa/major outbreak genotype, ST1, contained the vast majority of the 44 isolates (n = 38). As expected based on previous models of the *C. gattii* outbreak expansion [13], ST1 consisted of isolates exclusively from the initial outbreak and expansion zones, including British Columbia, Washington, and Oregon (Table S3). These results further validate the hypothesis that the epicenter of the outbreak was on Vancouver Island, beginning in the late 1990's, with a direct expansion into neighboring mainland British Columbia and subsequently into the United States [13]. The only exception in this dataset is isolate NIH444, an older isolate from the region that was isolated from a patient sputum sample in Seattle in the early 1970's [18], which is also identical at all 34 markers examined. This suggests that the VGIIa/major genotype responsible for most of the outbreak cases may have been circulating in the region prior to the outbreak. The possible travel history of this patient is unknown, and could therefore have involved exposure on Vancouver Island. Overall, this analysis provides increased evidence that the outbreak genotype is unique to the region thus far, and molecularly distinct from closely related isolates from both California and South America.

While the homogeneous nature of the VGIIa/major isolates based on robust molecular typing validated previous models, an underlying diversity within this group was also discovered. First, we further validated that the isolate ICB107 (ST13), from Brazil, was indeed distinct from the ST1 VGIIa/major clade. This isolate differs at one MLST marker (*LAC1*), and three VNTR markers (VNTR3, VNTR15, VNTR34). Additionally, the high-resolution sequence analysis was able to discriminate other VGIIa isolates that were collected from California. These include isolate CBS7750 (ST3), collected from the environment in San Francisco in 1990 [48], and isolate CA1014 (ST2), which was isolated from a patient with HIV infection in southern California. Each of these two isolates differs from ST1 due to unique mutations within the VNTR7 and VNTR34 loci, respectively. This shows that similar VGIIa genotype isolates have been found elsewhere, but that none are identical to those circulating as part of the ongoing Vancouver Island outbreak. Whether these isolates are a result of drift from ST1, or if ST1 arose from one of these related genotypes is not known.

In addition to discriminating VGIIa isolates that were not from the outbreak region, we also found a novel ST, ST30, which is highly similar to ST1, but divergent at a unique region of VNTR34. Interestingly, all three of the ST30 isolates are exclusively from Oregon, including two human clinical cases and one marine mammal case (Figure 1, Figure 3, Table S3). These results are consistent with an expansion followed by genetic drift in the highly variable VNTR loci. Isolates of ST30 have not been detected on Vancouver Island, indicating that this divergence is recent, and likely occurred after the expansion of ST1 into the United States. Alternatively, both ST1 (VGIIa/major) and ST30 may have been present for a long period, with only ST1 having been transferred to Vancouver Island.

To gain insights into the potential origins of the VGIIc genotype, and to assess its position within the overall VGII clade, clustering analysis was applied. Analysis of the combined dataset including 41 sequence types generated from 115 *C. gattii* isolates shows that the VGIIc genotype is independent, but similar to VGIIa (Figure 3). The closest relationship determined from the analysis was to ST34, an isolate from Colombia, which is also of the opposite **a** mating type. Moving beyond the direct branch, it appears that the VGIIc genotype shares sequence similarities to global isolates from South America, Africa, and also European isolates with likely African origins based on collected clinical case histories. Additionally, the VGIIc group also shares the *IGS1* allele with isolates from Australia, further obscuring the possible origins and necessitating a more thorough analysis (Figure 4A).

When the clustering analysis was expanded to include additional MLST loci (Figure 4A), both with and without the VNTR markers, the relationships of VGIIc to other global genotypes was further elucidated, with close relationships observed with global isolates from South America, Africa, Europe (Greece), and Australia (Figure 4B, Figure 4C, Table S4). These results increase the comprehensiveness of the analysis, and allow predictions of the relationship of this genotype to global isolates. Examination of alleles illustrates that, when the analysis is expanded, the VGIIc group appears to be more diverse from VGIIa and VGIIb. Each allele represented in green was initially denoted as an allele that was unique to the VGIIc genotype, with a total of seven such alleles (Figure 4A). To further elucidate the possible origins of these alleles, isolates selected based on their global diversity were sequenced at these loci (Figure 4A). Identical matches for four of the seven VGIIc-unique alleles were identified in isolates from Brazil, Australia, Europe, and European isolates with likely African origins, while three alleles (*SXI1*α, *HOG1*, and *CRG1*) remain unique to this novel genotype and only seen in Oregon thus far (Figure 4A).

To further characterize the genetic relationships among the global isolates in relation to the outbreak isolates, maximum likelihood (ML) analysis was applied. Initially, the isolates were characterized at 15 MLST loci, excluding the *MAT* locus so that both α and **a** isolates could be included. This analysis indicates that VGIIc may be more distantly related to the VGIIa/major genotype than initially observed. In addition, analysis of the 15 MLST loci shows a possible relation of VGIIc with isolates from South America, Africa, Europe, and Australia (Figure 4B). When this analysis was expanded to also include the four VNTR loci, similar results for the global comparisons of all genotypes and the relation of VGIIc to global isolates were observed (Figure 4C). For these reasons, additional sampling and analysis will be necessary to more precisely elucidate if this novel virulent genotype originated locally, or originated in an under-sampled region.

**Figure 5:**
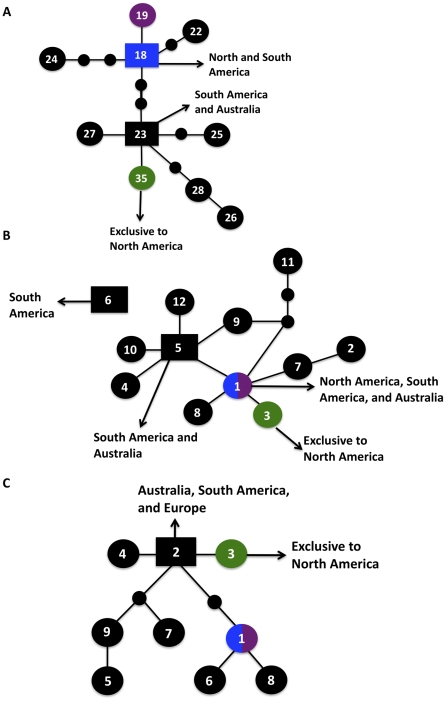
Haplotype networks define allele ancestry. Allele placements are indicated numerically, with the VGIIa/major genotype also represented by blue coloration, the VGIIb/minor genotype by purple coloration, and the VGIIc/novel genotype by green coloration. Large circles represent alleles extant in the population, and the small circles represent alleles that have not been recovered, or which may no longer be extant in the population. Each connecting line represents one postulated evolutionary event, with the squared allele representing the posited ancestral allele (two possible ancestral alleles depicted for *SXI1*α). A–C) Haplotype networks of the unique VGIIc alleles, *SXI1*α, *HOG1*, and *CRG1*, respectively, with geographic origins indicated.

**Figure 6:**
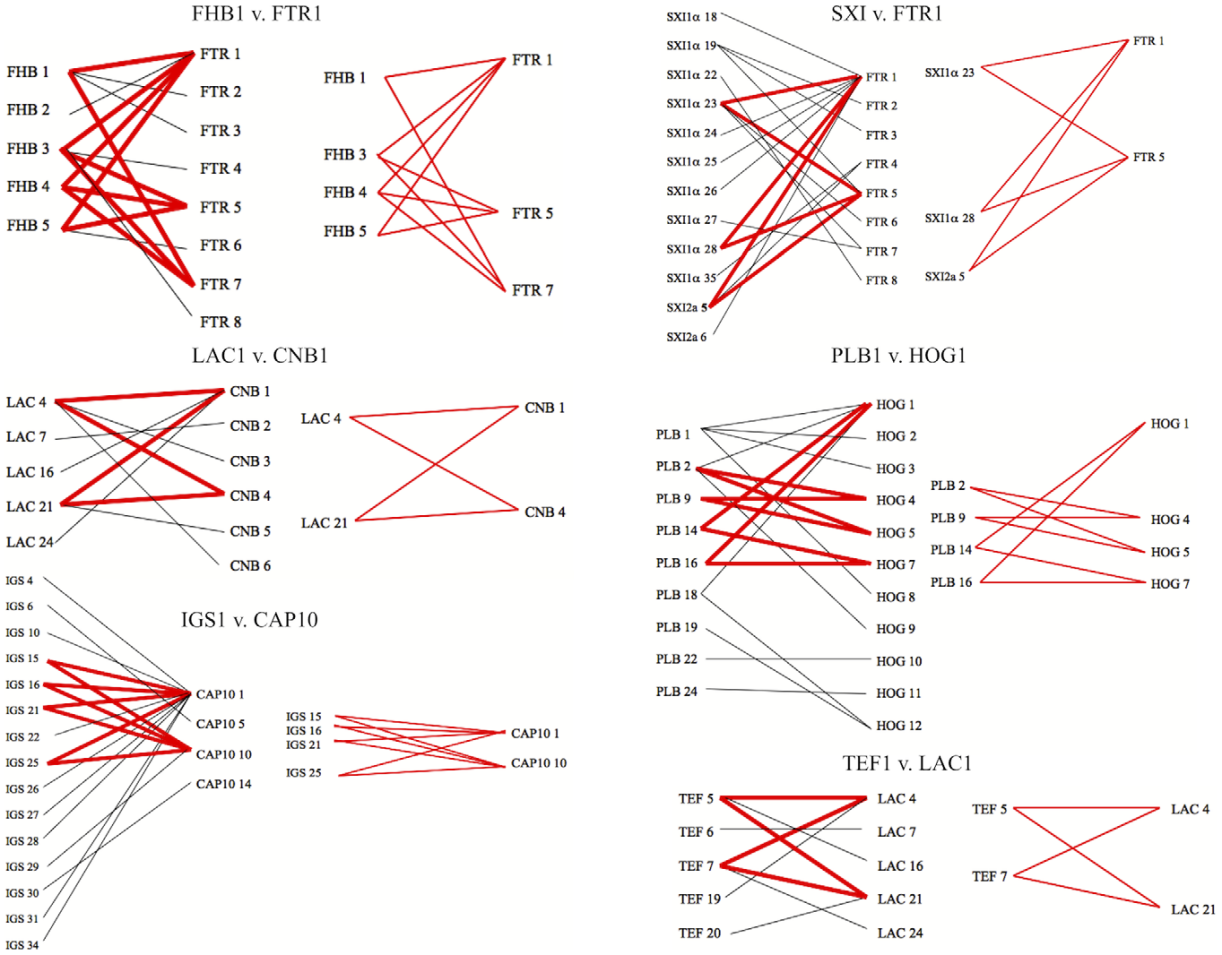
Evidence for recombination within the VGII molecular type. Informative paired allele graphs from VGII global isolates. An hourglass shape indicates the presence of all four possible pairs of alleles and serves as evidence for recombination. A total of 56 graphs with at least one possible recombining allele pair were generated from a set of 25 representative genotypes within the VGII molecular type, including isolates of both mating type **a** and α (see also Figure S4).

In addition to clustering analyses, TCS haplotype-mapping software was applied to establish the evolutionary histories of the MLST alleles examined during the analysis (Figure 5, Figure 6, Figure S3). From the sequence results, all of the VGIIc isolates were determined to be 100% identical, indicating that there was likely a recent emergence in which all of the isolates are clonally derived. To test this hypothesis, the TCS analysis allowed for the examination of individual loci to determine which alleles are likely ancestral, intermediate, or recently derived. Of the sixteen loci examined, eight were consistent with VGIIc possessing the ancestral allele, six of the alleles were distal nodes at the terminal end of the respective haplotype networks, and two loci were of intermediate allele positions.

Alleles with ancestral genotypes are less informative because these alleles may not have diversified over time in the VGIIc lineage for various reasons, including selection pressures and overall lack of diversity at the allele. When only non-ancestral alleles were examined, 75% lay at the distal ends of their haplotype maps. Intriguingly, the three VGIIc alleles unique to the genotype (*SXI1*α, *HOG1*, and *CRG1*) all have distal placements (Figure 5A–C). Additionally, the most recent ancestor to VGIIc in all three cases can be shown to derive from isolates that are from South America and Australia, indicating that VGIIc may have emerged out of one of these regions (Figure 5). While other regions including Europe and North America can be seen, no other regions are observed for all three of these alleles. These distal placements are consistent with a recent divergence of the unique VGIIc lineage. The haplotype analysis, in combination with the lack of any underlying diversity within the nine VGIIc isolates analyzed, indicates a recent emergence of this novel virulent genotype in Oregon.

To examine the role that recombination may have played in the population structure of the VGII molecular type, we conducted paired allele analysis for 25 representative global isolates (Figure 6, Figure S4). The discovery of all four possible allele combinations between two unlinked loci (AB, ab, Ab, aB) serves as evidence for likely recombination [49]. From this analysis, we show that isolates collected from South America, Africa, and Australia appear to be involved in recombination events. Representative VGIIa/major, VGIIb/minor, and VGIIc/novel isolates were found among groups of recombinant isolates. A group of ten isolates, all α, from South America and Africa (Figure S4) appeared most commonly as recombinant partners, although several **a** mating type isolates were also less frequently involved. In further support, when we examined the number of genotypes present by region and compared this data to the total number of genotypes represented (Figure S1), it is clear that South America and Africa populations are more diverse when compared with isolates from North America, which are more clonal. Additionally, while the observed diversity in Australia was lower than South America and Africa, this may be attributable to sampling bias of clonal regions as prior studies have shown that this continent is a region with high levels of recombination due to both same-sex and opposite-sex mating events [50]. In addition to the paired allele analysis, allele diagrams were constructed to observe possible recombination within individual MLST loci (Figure S5). The most parsimonious explanation for allelic diversity in 11 of the MLST loci analyzed is as a result of consecutive and/or independent mutations within the population. Within the four remaining loci, there exists at least one hybrid allele that may be the result of a recombination event between two hypothesized parental alleles in the global VGII population (Table 3, Figure S5). Phenotypic mating results were conducted and illustrate that the VGIIa/major (α), VGIIc/novel (α), VGII mating type **a** genotypes, as well as several of the proposed parental contributors from the allelic and genotypic recombination analysis show fertility with the production of spores when mated with fertile VGIII isolates (Table S5). Taken together, this suggests that both α-α and **a**-α mating events may be contributing to the formation of recombinant genotypes as well as the production of infectious spores. There were no examples of alleles introgressed into VGII from VGI, VGIII, or VGIV, in accord with findings that the four VG molecular types likely represent cryptic species [6], [29]. In summary, these results suggest that recombination events may be critical driving forces in the evolution of *C. gattii* VGII diversity, which may in part contribute to the generation of genotypes displaying increased virulence.

**Table 3:** Proposed recombinant alleles and hypothesized parental contributors.

Hypothesized recombinant alleles	Isolate/genotype	Hypothesized Parental alleles	Hypothesized parental isolates/genotypes*
IGS1- 4	*VGIIa*	IGS1-15	*VGIIc*, WA861, ICB184
		IGS1-16	ICB179, WM178
IGS1- 30	ICB183	IGS1-22	*2004/335*
		IGS1-26	*CBS8684*, 2003/125, 98/1037-2
HOG1-2	*NT-8*	HOG1-1	*VGIIa*, VGIIb, *99/473-1*, ***La499***, **La567**, ***La584*** ** ***CBS1930***, ICB179, WM178
		HOG1-3	*VGIIc*
		HOG1-7	*96/1120-1*, 2001/571
		HOG1-4	ICB184, 2003/125, 98/1037-2
HOG1-11	ICB97	HOG1-1	*VGIIa*, VGIIb, *99/473-1*, ***La499***, **La567**, ***La584*** ** ***CBS1930***, ICB179, WM178
		HOG1-3	*VGIIc*
		HOG1-7	96/1120-1, 2001/571
		HOG1-9	97/170
CRG1-5	WA861	CRG1-1	*VGIIa*, VGIIb, *99/473-1*, ***La499***, **La567**, ***La584*** ** ***CBS1930***, *2004/335*, ICB183, ICB182, *CBS8684*, 2003/125, 98/1037-2, *96/1120-1*, WM178
		CRG1-6	*93/980*
		CRG1-8	ICB179
		CRG1-9	2001/571
CAP59-5	2001/571	CAP59-3	WA861, *NT-8*
		CAP59-6	ICB179
CAP59-9	97/170	CAP59-2	VGIIb, *99/473-1*, ***La55***, ***La499***, **La567**, ***La584***, ***CBS1930***, ***CBS10090***, ICB184, 2003/125, 98/1037-2
		CAP59-7	*CBS8684*
		CAP59-3	WA861, *NT-8*

* Bold indicates MAT**a**, Italics indicates fertile representative.

### VGIIc/novel and VGIIa/major Outbreak Isolates Are Hypervirulent

**Figure 7:**
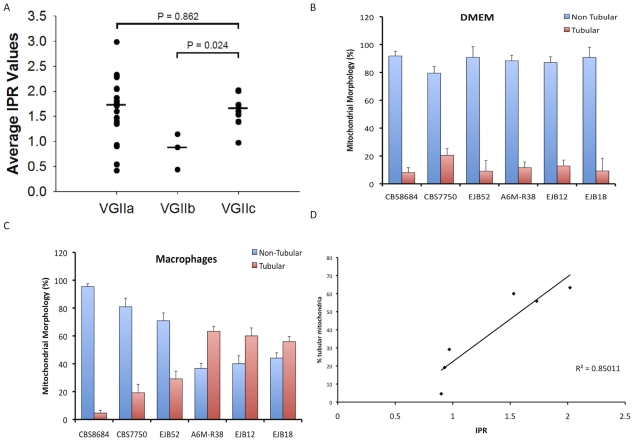
In vitro analyses of intracellular proliferation and mitochondrial morphology provide evidence the VGIIc genotype is hypervirulent. A) IPR rates of VGIIc isolates are similar to those from the VGIIa/major genotype and higher than those seen in the less-virulent VGIIb/minor genotype. Eight VGIIc isolates were tested individually, with the overall averages for the three primary outbreak genotypes presented. B) Percentage of cells with tubular mitochondrial morphology in DMEM. C) Percentage of cells with tubular mitochondrial morphology in macrophages. D) Linear correlation of IPR and percentage of tubular mitochondria after macrophage exposure.

It has recently been shown that intracellular proliferation rate (IPR) values for cryptococcal cells within macrophages are positively correlated with virulence in the murine model for cryptococcosis [31]. To further elucidate the potential virulence of outbreak isolates collected from the United States, proliferation rates of selected isolates were tested and compared to other isolates for which proliferation data had been previously obtained. In total, IPR values for eight of the nine VGIIc isolates were measured (Figure 7A). In addition, the type strains for VGIIa/major (R265) and VGIIb/minor (R272) were included as controls, and previously published data for other VGIIa and VGIIb isolates were included for comparisons [31]. On the basis of individual strains, seven of the eight VGIIc/novel isolates showed high IPR levels, with only a single outlier (EJB52) that had a low IPR value (0.97). Taken together, the median IPR value for VGIIc is significantly closer to that of VGIIa/major than to VGIIb/minor (Figure 7A). These results indicate that the VGIIc genotype has a similar intracellular phenotype, and thus virulence profile to the VGIIa/major genotype. This is noteworthy because previous analysis showed that the VGIIa/major genotype isolates from the outbreak had unusually high IPR values, and the VGIIc isolates from the same outbreak are here shown to have similarly high IPR values.

Another unique feature of the outbreak VGIIa/major isolates is the ability to form highly tubular mitochondria after intracellular parasitism, a characteristic that correlates with both IPR and murine virulence [31]. To explore the morphology of VGIIc isolates, we examined selected isolates in DMEM media and after exposure to macrophages. This analysis included two VGII environmental isolates (CBS8684, CBS7750) and four of the VGIIc/novel isolates. As expected, the vast majority of the mitochondria for all six isolates were non-tubular after exposure to DMEM media alone (Figure 7B). However, after exposure to macrophages, three of the four VGIIc isolates tested showed significantly higher percentages of tubular morphology (Figure 7C). The lone VGIIc isolate that did not exhibit this morphology (EJB52) was the same isolate that also had a low IPR value, and is thus an overall outlier for the VGIIc genotype.

When the results of IPR versus percentage of cells exhibiting tubular morphology were plotted, the graph showed a statistically significant correlation of the two measures with an R^2^ value of 0.85 (Figure 7D). These results further indicate that the VGIIc genotype is phenotypically similar to the Vancouver Island VGIIa/major outbreak strains. Our results also support evidence for similar mechanisms regulating the increased virulence seen in the novel VGIIc genotype. The exact roles that the mitochondrial tubular morphology might play in virulence are not yet known. However, the distinct phenotype is clearly unique to the outbreak isolates and is correlated with an increased ability to grow and divide within host innate immune cells.

**Figure 8:**
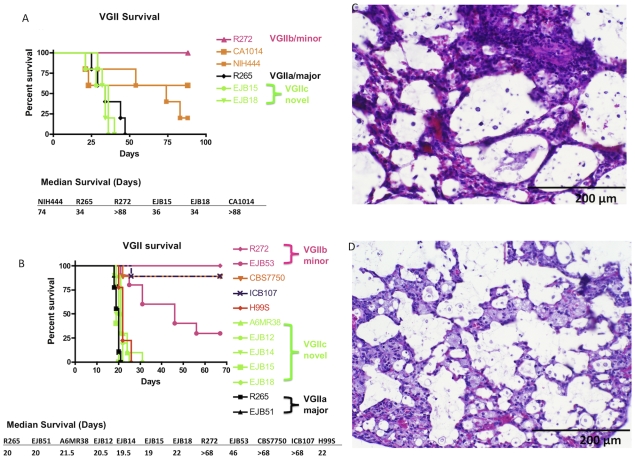
Isolates from the United States outbreak are hypervirulent. A) Groups of five animals were each infected with an infectious inoculum of 1.0×10^5^ cells of VGIIa isolates R265, CA1014, or NIH444, VGIIb isolate R272, or VGIIc isolates EJB15 or EJB18. B) Groups of nine or ten animals were each infected with an infectious inoculum of 5.0×10^4^ cells of VGIIa isolates R265, EJB51, CBS7750, or ICB107, VGIIb isolates R272 or EJB53, VGIIc isolates A6MR38, EJB12, EJB14, EJB15, or EJB18, or *C. neoformans* var. *grubii* isolate H99. C–D) Representative H&E stained histopathology slides from lung sections of severely morbid sacrificed animals from the VGIIa/major (R265) (C) and VGIIc (EJB18) (D) genotypes (sections from animals in panel B of this Figure).

The VGIIc isolates were found to be highly virulent in the murine inhalation model of infection. Two studies were conducted to examine virulence. In the first murine experiment a total of six isolates (n = 5 animals/isolate), were examined including two VGIIc isolates (Figure 8A). The VGIIa/major isolate R265 served as a positive control for high virulence, based on prior studies [6], and the VGIIc isolates EJB15 and EJB18 showed similar virulence with this well characterized virulent isolate. Additionally, two VGIIa isolates that are not hypothesized to be from the current Vancouver Island outbreak, including NIH444, which is fully identical across 34 markers, and isolate CA1014, which differs from R265 at VNTR34, show a significant reduction in virulence compared to the high virulence isolates (P<0.05). Finally, in accordance with previous studies, the VGIIb/minor type strain R272 from Vancouver Island was avirulent in this model.

The analysis of virulence within the VGII genotype was extended in a second experiment, in which 12 isolates (n = 9–10 animals/isolate) were examined. This study included two VGIIa/major isolates from the outbreak zone, two VGIIb/minor isolates from the outbreak zone, five of the novel VGIIc isolates, two VGIIa-related isolates that are not part of the outbreak, and the *C. neoformans* var. *grubii* type strain, H99. The H99 isolate used (H99S) has been shown to be highly virulent in the murine model of infection [44], [51].

As expected, all five of the VGIIc isolates from Oregon as well as the VGIIa/major isolates from Vancouver Island and Oregon, and the highly virulent H99 isolate exhibited a high level of virulence (median survival = 20.6 days). The VGIIb/minor isolates tested were significantly decreased in virulence compared to the more virulent VGIIa and VGIIc genotypes (P<0.005). The VGIIb isolate R272 was avirulent whereas the VGIIb isolate EJB53 from Oregon exhibited significantly less virulence compared to the VGIIa/major and VGIIc isolates (P<0.005, median survival = 46 days). Similar to the first animal study, two VGIIa isolates that differ at one or more molecular markers from the major VGIIa outbreak genotypes were also tested. The environmental isolate CBS7750 and a clinical isolate from South America ICB107 were significantly attenuated (P<0.005) (Figure 8B). These results provide further evidence that these are related to but distinguishable from isolates that are specific to the Vancouver Island outbreak, and subsequent United States expansion, and are decreased in ability to mount fatal infections in a mouse intranasal instillation model of infection.

The cause of infection was further evaluated by histopathological analysis of lung sections recovered from two infected animals per isolate at sacrifice. Harvested organs were processed and sectioned for slides with H&E staining. The lungs from the virulent isolates showed significant inflammation and numerous cryptococcal cells dispersed throughout the alveoli, in accordance with severe pulmonary infection. Our findings show that there are no major clinical differences between pulmonary infections with the infectious genotypes VGIIa/major (Figure 8C), and the novel VGIIc genotype (Figure 8D). These results further support similar disease progression caused by these two highly virulent outbreak genotypes.

## Discussion

The findings presented here document that the outbreak of *C. gattii* in Western North America is continuing to expand throughout this temperate region, and that the outbreak isolates in the United States of both the VGIIa/major genotype and the novel VGIIc genotype are clonally derived and highly virulent in host models of infection. These conclusions are based on an extensive molecular analysis of isolates collected from the United States (Table 2) and a comprehensive global collection of VGII isolates of diverse geographic origin (Figure S1), examining both conserved and divergent regions of the genome. The virulence analysis is based on assays in both murine derived macrophages and mice. These findings demonstrate that this emerging and fatal outbreak is continuing to expand, and that the virulence of these isolates is unusually high when compared to isolates of closely related but distinguishable genotypes found in other non-outbreak regions.

The continued expansion of *C. gattii* in the United States is ongoing, and the diversity of hosts increasing. Cases have been observed in urban and rural areas, and have occurred in a range of mammals [16], [52]. On Vancouver Island and the mainland of British Columbia, cases have been documented in marine and terrestrial mammals including cats, dogs, porpoises, ferrets, and llamas [15], [52], [53]. This trend has continued in the United States, with several cases in agrarian, domestic, and wild terrestrial mammals, as well as marine mammals, adding elk, alpacas, and sheep to the aforementioned list (Table S1) [13], [14], [17]. The co-expansion of the outbreak among mammals and humans is significant for several reasons. Non-migratory mammals serve as sentinels for disease expansion, particularly given that isolation of *C. gattii* from the environment is difficult, and not yet successful at all in Oregon. Additionally, the threat to agricultural and domestic animals is significant and thus the need for cooperation among health officials is critical. Finally, the widespread spectrum of disease illustrates that the organism is likely to be pervasive in the environment, and that physicians and veterinarians should be well informed of symptoms to facilitate early diagnoses, and successful isolate collection and tracking.

A major question in the study of this outbreak is whether sexual recombination, either within or between mating types, is occurring or has occurred in the region. The possibility of meiosis is important for two reasons. The first is that sexual recombination is postulated to be a driving force for the increased virulence of the VGIIa/major genotype, supported by the discovery of a diploid VGIIa/major isolate, an intermediate in unisexual mating (all nine VGIIc/novel isolates are haploid) [6], [36]. *C. gattii* has also been shown to undergo opposite sex mating in the laboratory, although this has not yet been observed to occur between two isolates of the VGII molecular type [36], [54], [55]. Studies in *C. neoformans* have shown that this related pathogen completes a full **a**-α sexual cycle in association with plants [56]. Additionally, a recent study of environmentally sampled Australian VGI isolates demonstrated evidence for recombination via both opposite and same-sex mating [50]. Taken together, available evidence indicates that both opposite and same-sex mating are naturally occurring in populations. This evidence lends support to the hypothesis that meiosis might be a factor in the forces that are driving high virulence in the outbreak region.

The second major event that results from sexual processes in the pathogenic *Cryptococcus* species is the formation of spores. Small spores ranging from 1–2 µm in diameter have been observed to be produced in large numbers as the result of opposite sex mating in both *C. neoformans* and *C. gattii* [57], [58]. Studies by Lin and colleagues showed that sexual spores can be produced as the result of a meiotic process occurring between cells of the same mating type, a process referred to as unisexual or same-sex mating [59]. Several studies have shown spores to be pathogenic in animal models of infection. Two previous studies both showed evidence for virulence of *Cryptococcus* spores, and in one case provided evidence for enhanced virulence compared to yeast cells [60], [61]. More recently, studies have shown that *Cryptococcus neoformans* spores are indeed virulent in the murine intranasal instillation model of infection [44], [62], providing evidence that spores should be considered as infectious propagules in models examining infections, expansion, and emergence of both *C. neoformans* and *C. gattii*. Given that all of the Pacific NW isolates are α mating type, and particles small enough to be spores are present in the air [26], [63], the most parsimonious model is that if these are spores, they are produced via α-α unisexual reproduction.

Our findings further indicate that mitochondria may play a significant role in the increased virulence seen in the outbreak isolates [31]. Tubular morphology and the increased ability to proliferate within immune cells indicate that the ability to proliferate and survive within host cells is fundamental to virulence. The possible role of mitochondrial involvement is intriguing and also increasingly relevant based on studies that have shown mitochondrial inheritance and recombination may impact *C. gattii* evolution, with the inheritance of the mitochondrial genome from the **a** mating type parent in opposite-sex mating [64], [65]. Future studies in this area should address the roles that mitochondrial genes, or nuclear genes that regulate mitochondria may play in the hypervirulence observed in the outbreak isolates. Furthermore, it may be that cell-cell fusion events via mating and mitochondrial exchange without meiosis or nuclear genetic exchange have played roles in recombination and virulence acquisition in naturally occurring *C. gattii* populations [64], [65].

A central question in the field lies in the possible origins of the virulent genotypes. For the VGIIa and VGIIc lineages, it is clear that those are unique to the Pacific NW, and either arose there locally, or were transferred from an under-sampled region (Australia, South America, Africa). Isolates that are related to, but distinct at one or more molecular marker from VGIIa have been identified in San Francisco (CBS7750), southern California (CA1014), and South America (ICB107). However, in each of these cases, the isolates are not identical with the VGIIa/major isolates from the Pacific NW. Whether the outbreak isolates are derived from these isolates, or alternatively that these isolates are derived from the outbreak lineage is at present unclear. In the VGIIb/minor outbreak lineage, isolates from Australia are identical at all 30 MLST loci and four VNTRs analyzed, and the most parsimonious model is that the two are directly related. While it is conceivable that both the Australian and the Vancouver Island VGIIb/minor genotype isolates were dispersed independently from another geographic locale, until isolates are identified conclusively from another locale the most parsimonious model is transfer from Australia to the Pacific NW. We note that a single isolate with a related but distinct genotype (isolate 99/473) from the Caribbean has been identified; and other isolates have been reported to share the VGIIb genotype but have been analyzed at a limited number of MLST markers (n = 7) which is insufficient to establish how closely related these isolates are to the outbreak VGIIb/minor genotype strains [29]. The origins of VGIIc are unclear, with the genotype possibly arriving in the Pacific NW from South America, Africa, Europe, or Australia. Alternatively, this novel unique genotype may have arisen locally.

As for the geographic origins of VGII diversity, this also remains to be established and may involve populations in Australia, South America, and Africa. It is clear that there is considerable diversity among isolates from South America. As we originally proposed as an alternative model [6], and has been independently presented by other investigators (W. Meyer, T. Boekhout, JP Xu, pers. comm.), South America may represent a source of diversity and ongoing generation of novel isolates. Analysis of 8 MLST loci in this study indicates that in South America and the Caribbean there are 14 genotypes seen in 21 isolates, while in North America only 3 genotypes have been observed through the analysis of 64 isolates (Figure S1). Additionally, there is accumulating evidence that fertile isolates of both **a** and α mating type are present in South America [29], and thus ongoing **a**-α opposite sex mating may be occurring there. It is also clear that a unique set of VGII isolates are circulating in Australia, and there is evidence for ongoing recombination in α only and **a**-α populations, suggesting that mating contributes to the generation of diversity in Australia [36], [49], [54], [55], [66], [67]. Finally, the analysis of global VGII isolates reveals genetic diversity in Africa, and given the recent findings that *C. neoformans* likely originated in sub-Saharan Africa (A. Litvintseva and T. Mitchell, pers. Comm.), further analysis of African *C. gattii* isolates is clearly warranted.

It remains possible that South America, Africa, or both represent the ancestral populations of *C. gattii*, and that more recent dispersal events from other established populations (for example, from Australia to the Pacific Northwest) have occurred to contribute to the outbreak. As yet, all of the isolates found in the Pacific Northwest are α mating type. Thus, if sexual reproduction is occurring in the Pacific Northwest, it would appear to involve same-sex mating occurring under environmental conditions. Recent studies have documented that *C. neoformans* and *C. gattii* are stimulated to undergo opposite-sex mating in laboratory conditions that simulate environmental niches (pigeon guano medium, co-culture with plants) and thus similar conditions may be necessary in nature [56], [68]. Overall, both the VGIIa/major and the VGIIc/novel genotypes contain a number of MLST loci that are thus far restricted to these lineages, and their origins remain to be identified.

Independently of the variables leading up to and influencing this outbreak, the major concern is and continues to be the inexorable expansion throughout the region. From 1999 through 2003, the cases were largely restricted to Vancouver Island. Between 2003 and 2006, the outbreak expanded into neighboring mainland British Columbia and then into Washington and Oregon from 2005 to 2009. Based on this historical trajectory of expansion, the outbreak may continue to expand into the neighboring region of Northern California, and possibly further.

The rising incidence of cryptococcosis cases in humans and animals highlights the need for enhanced awareness in the region, and those regions that may potentially become involved. While rare, little is currently known about how or why specific humans and animals become infected. Increased vigilance may decrease the time from infection to diagnosis, and thus lead to more effective treatment and a reduction in mortality rates. The potential dangers of travel-associated risks should be noted, as a growing number of cases attributable to travel within the Pacific NW region have been documented [69], [70]. Northern California has similar temperate climates to endemic regions within Oregon, leading to the hypothesis that the emergence may expand there, while expansion eastward may be limited by winters with average temperatures often below freezing [17].

The expansion of the outbreak into California is plausible based on several studies documenting the presence of *C. gattii* throughout the state and in Mexico. *C. gattii* molecular type VGII was environmentally isolated in the San Francisco area in 1990 (isolate CBS7750) [48], and there have also been two confirmed and one travel-associated case of *C. gattii* molecular type VGI in California. Of the VGI cases, one occurred in a male Atlantic bottlenose dolphin in San Diego, one was isolated from a liver transplant recipient in San Francisco, and the other from an otherwise healthy patient in North Carolina with travel history to the San Francisco region [71], [72], [73]. In addition *C. gattii* has been reported in southern California among a cohort of HIV/AIDS patients [74]. Recently, studies of clinical isolates from Mexico revealed all four molecular types of *C. gattii* to be present [75]. Taken together, the hypothesis that the virulent isolates from the Pacific NW will expand into California must be considered by both physicians and public health officials.

During the coming years, monitoring and researching the outbreak expansion as a multidisciplinary effort will be critical. The ability to bring diverse groups of professionals interested in *C. gattii* expansion has been greatly facilitated through the formation of the *Cryptococcus gattii* working group of the Pacific Northwest [17]. From a research standpoint, further examination of the molecular mechanisms underlying the increased virulence in both VGIIa/major and VGIIc/novel will be useful for the development of aggressive treatments that may be needed. Furthermore, increased efforts to determine the ecology and population dynamics of *C. gattii* in the region, and elucidating the evolutionary history of the VGIIc genotype will be critical to gain further insights into the origins of this unprecedented and frequently fatal fungal outbreak.

## Supporting Information

Figure S1 MLST of all VGII isolates used in the study and the four out-group isolates used in the phylogenetic analysis.
(0.07 MB PDF)

Figure S2 MLST analysis of Vancouver Island isolates at 2 loci. These were chosen to determine if any of the isolates might have belonged to the VGIIc group.
(0.02 MB PDF)

Figure S3 TCS haplotype networks for the thirteen alleles not represented in Figure 5 of the main text.
(0.15 MB PDF)

Figure S4 All paired allele graphs from VGII global isolates generated during the analysis. Isolates of both mating type **a** and α were included. In addition, a group of ten isolates, all α, from South America and Africa appeared most commonly as recombinant partners and are illustrated.
(0.12 MB PDF)

Figure S5 Allelic recombination analysis for 15 loci indicates that 11 are likely derived from consecutive and/or independent mutations within the population. The four other loci show at least one hybrid allele that may be the result of a recombination event between two proposed parental alleles in the global VGII population. Squared alleles represent likely recombinants, while circled alleles indicate proposed parental contributors. Each of the possible contributors is indicated by a respective color.
(1.34 MB PDF)

Table S1 Primers used in the study.
(0.03 MB XLS)

Table S2 GenBank accession numbers for all of the MLST and VNTR alleles represented in the text and figures.
(0.05 MB XLS)

Table S3 Detailed sequence type information from Figure 3.
(0.03 MB XLS)

Table S4 Detailed sequence type information from Figure 4B and Figure 4C.
(0.02 MB XLS)

Table S5 Mating properties of selected VGII isolates.
(0.02 MB XLS)
