# Supplementary material for: Emergence and Pathogenicity of Highly Virulent Cryptococcus gattii Genotypes in the Northwest United States
Source: PLoS Pathog. 2010 Apr 22;6(4):e1000850. doi: 10.1371/journal.ppat.1000850 (PMC2858702; doi:10.1371/journal.ppat.1000850)
Supplement: Figure S1 — MLST of all VGII isolates used in the study and the four out-group isolates used in the phylogenetic analysis. (0.07 MB PDF) [file ppat.1000850.s001.pdf]

Name	*SXI1*α allele 1,354 bp	*SXI2*a allele 2,529 bp	*IGS* allele 740 bp	*TEF1* allele 700 bp	*GPD1* allele 547 bp	*LAC1* allele 554 bp	*CAP10* allele 568 bp	*PLB1* allele 600 bp	*MPD1* allele 677 bp	Strain origin
NT-10	3	-	3	4	5	11	4	5	3	Northern Territory, Australia clinical
WM276	7	-	3	4	5	5	4	5	3	South Australia, Australia environmental
NIH312	16	-	18	10	9	2	6	23	8	clinical
MMRL2651	12	-	8	2	4	1	7	3	2	Botswana clinical
ICB107	18	-	4	7	1	24	1	1	5	Brazil clinical
R265	18	-	4	7	1	4	1	1	5	Vancouver Island clinical
R268	18	-	4	7	1	4	1	1	5	Vancouver Island clinical
CBS7750	18	-	4	7	1	4	1	1	5	San Francisco, USA environmental
F3179	18	-	4	7	1	4	1	1	5	Vancouver Island, Canada clinical
F2932	18	-	4	7	1	4	1	1	5	Vancouver Island, Canada clinical
R269	18	-	4	7	1	4	1	1	5	Vancouver Island clinical
R271	18	-	4	7	1	4	1	1	5	Vancouver Island clinical
152	18	-	4	7	1	4	1	1	5	Vancouver Island environmental
RB1	18	-	4	7	1	4	1	1	5	Vancouver Island environmental
RB2	18	-	4	7	1	4	1	1	5	Vancouver Island environmental
RB4	18	-	4	7	1	4	1	1	5	Vancouver Island environmental
NIH444	18	-	4	7	1	4	1	1	5	Seattle, USA clinical
R376	18	-	4	7	1	4	1	1	5	Vancouver Island, Canada clinical
AOR432	18	-	4	7	1	4	1	1	5	Vancouver Island, Canada clinical
R498	18	-	4	7	1	4	1	1	5	Vancouver Island, Canada veterinary
R540	18	-	4	7	1	4	1	1	5	Vancouver Island, Canada clinical
F2596	18	-	4	7	1	4	1	1	5	Vancouver Island, Canada veterinary
F3016	18	-	4	7	1	4	1	1	5	Vancouver Island, Canada veterinary
125	18	-	4	7	1	4	1	1	5	Vancouver Island, Canada environmental
152A-3	18	-	4	7	1	4	1	1	5	Vancouver Island, Canada environmental
RB39	18	-	4	7	1	4	1	1	5	Vancouver Island, Canada environmental
RB45	18	-	4	7	1	4	1	1	5	Vancouver Island, Canada environmental
RB59	18	-	4	7	1	4	1	1	5	Vancouver Island, Canada environmental
T67707	18	-	4	7	1	4	1	1	5	**San Juan Island, WA, clinical**
EJB3	18	-	4	7	1	4	1	1	5	**OR State clinical**
EJB19	18	-	4	7	1	4	1	1	5	**OR State clinical**
EJB4	18	-	4	7	1	4	1	1	5	**WA State clinical**
EJB5	18	-	4	7	1	4	1	1	5	**WA State clinical**
EJB6	18	-	4	7	1	4	1	1	5	**WA State clinical**
EJB7	18	-	4	7	1	4	1	1	5	**WA State clinical**
EJB8	18	-	4	7	1	4	1	1	5	**WA State clinical**
EJB9	18	-	4	7	1	4	1	1	5	**WA State clinical**
EJB13	18	-	4	7	1	4	1	1	5	**WA State clinical**
W15209	18	-	4	7	1	4	1	1	5	**WA State clinical**
EJB16	18	-	4	7	1	4	1	1	5	**OR State alpaca**
EJB17A	18	-	4	7	1	4	1	1	5	**OR State canine**
EJB17B	18	-	4	7	1	4	1	1	5	**OR State canine**
3700 (1)	18	-	4	7	1	4	1	1	5	**WA State porpoise**
3700 (2)	18	-	4	7	1	4	1	1	5	**WA State porpoise**
3635	18	-	4	7	1	4	1	1	5	**WA State porpoise**
3059	18	-	4	7	1	4	1	1	5	**WA State porpoise**
EJB21	18	-	4	7	1	4	1	1	5	**OR State porpoise**
MMC08-1042	18	-	4	7	1	4	1	1	5	**OR State clinical**
KB11632	18	-	4	7	1	4	1	1	5	**OR State clinical**
EJB22	18	-	4	7	1	4	1	1	5	**OR State canine**
EJB51	18	-	4	7	1	4	1	1	5	**OR State alpaca**
EJB54	18	-	4	7	1	4	1	1	5	**OR State feline**
EJB77	18	-	4	7	1	4	1	1	5	**OR State canine**
CA1014	18	-	4	7	1	4	1	1	5	**CA clinical**
EJB10	19	-	10	5	6	4	1	2	5	**OR State clinical**
MMC08-896	19	-	10	5	6	4	1	2	5	**OR State canine**
EJB53	19	-	10	5	6	4	1	2	5	**OR state Elk**
R272	19	-	10	5	6	4	1	2	5	Vancouver Island, Canada clinical
Arn1	19	-	10	5	6	4	1	2	5	Australia environmental
RB31	19	-	10	5	6	4	1	2	5	Vancouver Island, Canada environmental
Ram2	19	-	10	5	6	4	1	2	5	Australia environmental
Ram5	19	-	10	5	6	4	1	2	5	Australia environmental
RDH-6	19	-	10	5	6	4	1	2	5	Northern Territory, Australia clinical
V5/571_063	19	-	10	5	6	4	1	2	5	Sydney, Australia veterinary
V26/571_147	19	-	10	5	6	4	1	2	5	Sydney, Australia veterinary
V21/571_118	19	-	10	5	6	4	1	2	5	Sydney, Australia veterinary
V9/571_073	19	-	10	5	6	4	1	2	5	Sydney, Australia veterinary
V4/571_058	19	-	10	5	6	4	1	2	5	Sydney, Australia veterinary
NT-6	19	-	10	5	6	4	1	2	5	Northern Territory, Australia clinical
NT-13	19	-	10	5	6	4	1	2	5	Northern Territory, Australia clinical
V20/571_116	19	-	10	5	6	4	1	2	5	Sydney, Australia veterinary
NT-12	19	-	10	5	6	4	1	2	5	Northern Territory, Australia clinical
Ram15	19	-	10	5	6	4	1	2	5	Australia environmental
NT-7	19	-	10	5	6	4	1	2	5	Northern Territory, Australia clinical
EJB76	19	-	10	5	6	4	1	2	5	**OR state feline**
99/473	19	-	10	5	6	4	1	2	5	Caribbean Islands clinical
EJB12	35	-	15	7	6	4	1	1	5	**OR State clinical**
EJB18	35	-	15	7	6	4	1	1	5	**OR State clinical**
EJB14	35	-	15	7	6	4	1	1	5	**OR State feline**
EJB15	35	-	15	7	6	4	1	1	5	**OR State alpaca**
A6MR38	35	-	15	7	6	4	1	1	5	**OR State clinical**
EJB52	35	-	15	7	6	4	1	1	5	**OR State feline**
EJB55	35	-	15	7	6	4	1	1	5	**OR State Ovine**
EJB74	35	-	15	7	6	4	1	1	5	**OR State feline**
EJB75	35	-	15	7	6	4	1	1	5	**OR State canine**
LA499	-	5	25	7	6	4	1	1	5	**Colombia clinical**
LA584	-	5	25	7	6	4	1	18	5	**Colombia clinical**
CBS1930	-	5	25	7	6	4	1	18	5	Aruba veterinary
LA567	-	6	34	7	6	4	1	18	5	**Colombia clinical**
LA55	-	5	31	7	27	4	1	1	5	**Brazil clinical**
CBS10090	-	5	31	7	27	4	1	1	5	**Greece clinical**
ICB183	23	-	30	7	21	4	14	18	5	Brazil environmental
WA 861	23	-	15	7	16	4	10	9	5	Western Australia, Australia veterinary

ICB184	23	-	15	19	6	4	1	2	5	Brazil environmental
2004/335	23	-	22	7	6	4	1	2	5	French Guyana clinical
2004/606	23	-	22	7	6	4	1	2	5	French Guyana clinical
2004/681	23	-	22	7	6	4	1	2	5	French Guyana clinical
98/1132	23	-	22	7	6	4	1	2	5	Caribbean Islands clinical
2006/00194	23	-	22	7	6	4	1	2	5	**French Guyana clinical**
ICB182	23	-	21	20	6	21	1	2	5	Brazil clinical
BR189	23	-	21	20	6	21	1	2	5	**Brazil environmental**
97/170	23	-	28	5	21	21	1	2	5	French Guyana clinical
93/980	23	-	27	5	22	21	1	19	5	France clinical
CBS8684	27	-	26	5	6	21	1	22	5	Uruguay environmental
2003/125	19	-	26	7	25	21	1	9	5	France clinical (Africa)
98/1037-2	28	-	26	7	25	21	1	9	5	France clinical (N. Africa)
98/1037-1	28	-	26	7	25	21	1	9	5	France clinical (N. Africa)
ICB97	28	-	25	7	24	4	10	24	5	Brazil clinical
ICB179	28	-	16	7	6	4	10	16	5	Brazil environmental
ICB180	28	-	16	7	6	4	10	16	5	Brazil environmental
99/901-1	26	-	21	19	21	4	10	16	5	France clinical (Ivory Coast)
99/901-2	26	-	21	19	21	4	10	16	5	France clinical (Ivory Coast)
2000/87	26	-	21	19	21	4	10	16	5	France clinical (Ivory Coast)
2001/935-1	26	-	21	19	21	4	10	16	5	Senegal clinical
96/1120-1	26	-	21	19	21	4	10	16	5	France clinical (Central Africa)
96/1120-2	26	-	21	19	21	4	10	16	5	France clinical (Central Africa)
2001/571	25	-	29	7	6	4	10	14	5	France clinical (Central Africa)
WM178	22	-	16	5	17	16	1	14	5	Sydney, Australia clinical
RDH-2	24	-	6	6	2	7	5	1	5	Northern Territory, Australia clinical
MMRL2647	24	-	6	6	2	7	5	1	5	Sydney, Australia clinical
NT-8	24	-	6	6	2	7	5	1	5	Northern Territory, Australia clinical
RDH-9	24	-	6	6	2	7	5	1	5	Northern Territory, Australia clinical
RDH-7	24	-	6	6	2	7	5	1	5	Northern Territory, Australia clinical
NT-3	24	-	6	6	2	7	5	1	5	Northern Territory, Australia clinical
MMRL1340	24	-	6	6	2	7	5	1	5	Australia clinical
