# Supplementary material for: Emergence and Pathogenicity of Highly Virulent Cryptococcus gattii Genotypes in the Northwest United States
Source: PLoS Pathog. 2010 Apr 22;6(4):e1000850. doi: 10.1371/journal.ppat.1000850 (PMC2858702; doi:10.1371/journal.ppat.1000850)
Supplement: Figure S2 — MLST analysis of Vancouver Island isolates at 2 loci. These were chosen to determine if any of the isolates might have belonged to the VGIIc group. (0.02 MB PDF) [file ppat.1000850.s002.pdf]

Isolate	IGS	GPD1	Strain Origin
VGIIa	4	1	R265 VGIIa/major control
VGIIb	10	6	R272 VGII/minor control
VGIIc	15	6	EJB18 VGIIc/novel control
123	4	1	Vancouver Island, Canada Environmental
124	4	1	Vancouver Island, Canada Environmental
130	4	1	Vancouver Island, Canada Environmental
131	4	1	Vancouver Island, Canada Environmental
153	4	1	Vancouver Island, Canada Environmental
113A-5	4	1	Vancouver Island, Canada Environmental
129A-1	4	1	Vancouver Island, Canada Environmental
152-4	4	1	Vancouver Island, Canada Environmental
152-6	4	1	Vancouver Island, Canada Environmental
152A-1	4	1	Vancouver Island, Canada Environmental
152A-2	4	1	Vancouver Island, Canada Environmental
152A-4	4	1	Vancouver Island, Canada Environmental
E113	4	1	Vancouver Island, Canada Environmental
F1851	4	1	Vancouver Island, Canada Environmental
F2310	4	1	Vancouver Island, Canada Environmental
F3197	4	1	Vancouver Island, Canada Environmental
MAC9	4	1	Vancouver Island, Canada Environmental
99MR10	4	1	Vancouver Island, Canada Environmental
R273	4	1	Vancouver Island, Canada Environmental
R322	4	1	Vancouver Island, Canada Environmental
R369	4	1	Vancouver Island, Canada Environmental
R406	4	1	Vancouver Island, Canada Environmental
R409	4	1	Vancouver Island, Canada Environmental
R507	4	1	Vancouver Island, Canada Environmental
RB3	4	1	Vancouver Island, Canada Environmental
RB5	4	1	Vancouver Island, Canada Environmental
RB17	4	1	Vancouver Island, Canada Environmental
RB50	4	1	Vancouver Island, Canada Environmental
152A-5	4	1	Vancouver Island, Canada Environmental
RB7	4	1	Vancouver Island, Canada Environmental
RB11	4	1	Vancouver Island, Canada Environmental
RB13	4	1	Vancouver Island, Canada Environmental
RB14	4	1	Vancouver Island, Canada Environmental
RB15	4	1	Vancouver Island, Canada Environmental
RB18	4	1	Vancouver Island, Canada Environmental
RB20	4	1	Vancouver Island, Canada Environmental
RB22	4	1	Vancouver Island, Canada Environmental
RB26	4	1	Vancouver Island, Canada Environmental
RB29	4	1	Vancouver Island, Canada Environmental
RB30	4	1	Vancouver Island, Canada Environmental
RB33	4	1	Vancouver Island, Canada Environmental
RB34	4	1	Vancouver Island, Canada Environmental
RB35	4	1	Vancouver Island, Canada Environmental
RB37	4	1	Vancouver Island, Canada Environmental
RB40	4	1	Vancouver Island, Canada Environmental

RB42	4	1	Vancouver Island, Canada Environmental
RB46	4	1	Vancouver Island, Canada Environmental
RB48	4	1	Vancouver Island, Canada Environmental
RB54	4	1	Vancouver Island, Canada Environmental
RB56	4	1	Vancouver Island, Canada Environmental
RB58	4	1	Vancouver Island, Canada Environmental
129	10	6	Vancouver Island, Canada Environmental
133	10	6	Vancouver Island, Canada Environmental
152A-6	10	6	Vancouver Island, Canada Environmental
RB52	10	6	Vancouver Island, Canada Environmental
RB57	10	6	Vancouver Island, Canada Environmental
