# Supplementary figures and images for: Emergence and Pathogenicity of Highly Virulent Cryptococcus gattii Genotypes in the Northwest United States
Source: PLoS Pathog. 2010 Apr 22;6(4):e1000850. doi: 10.1371/journal.ppat.1000850 (PMC2858702; doi:10.1371/journal.ppat.1000850)

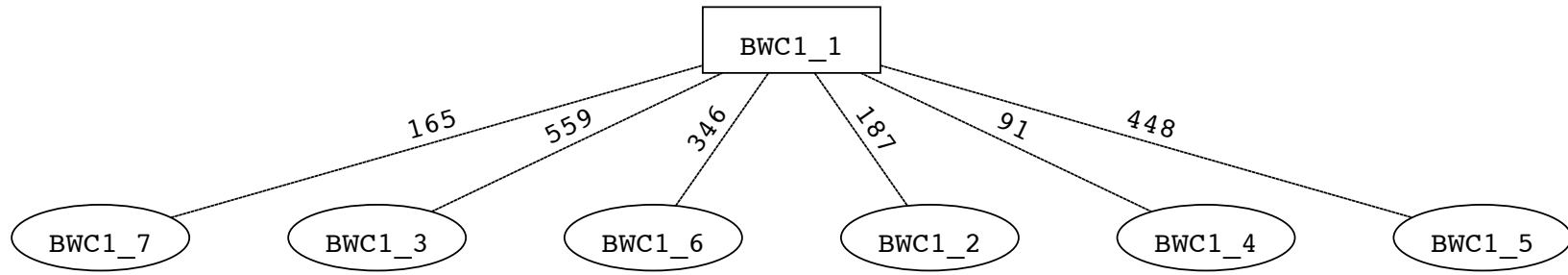

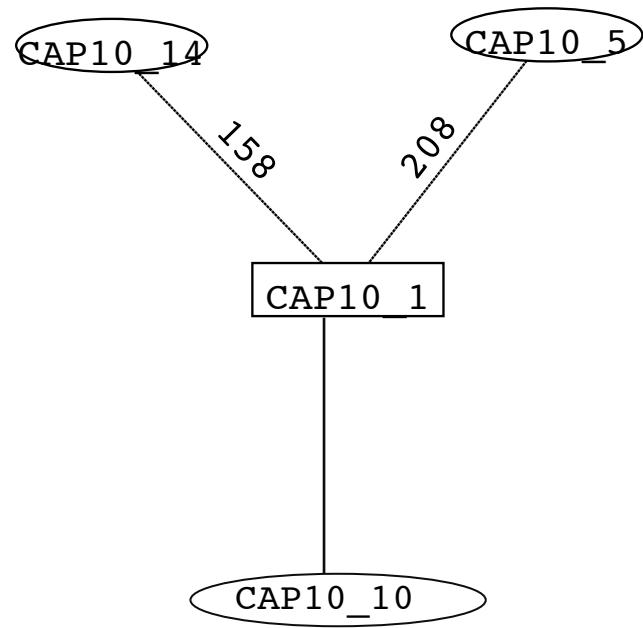

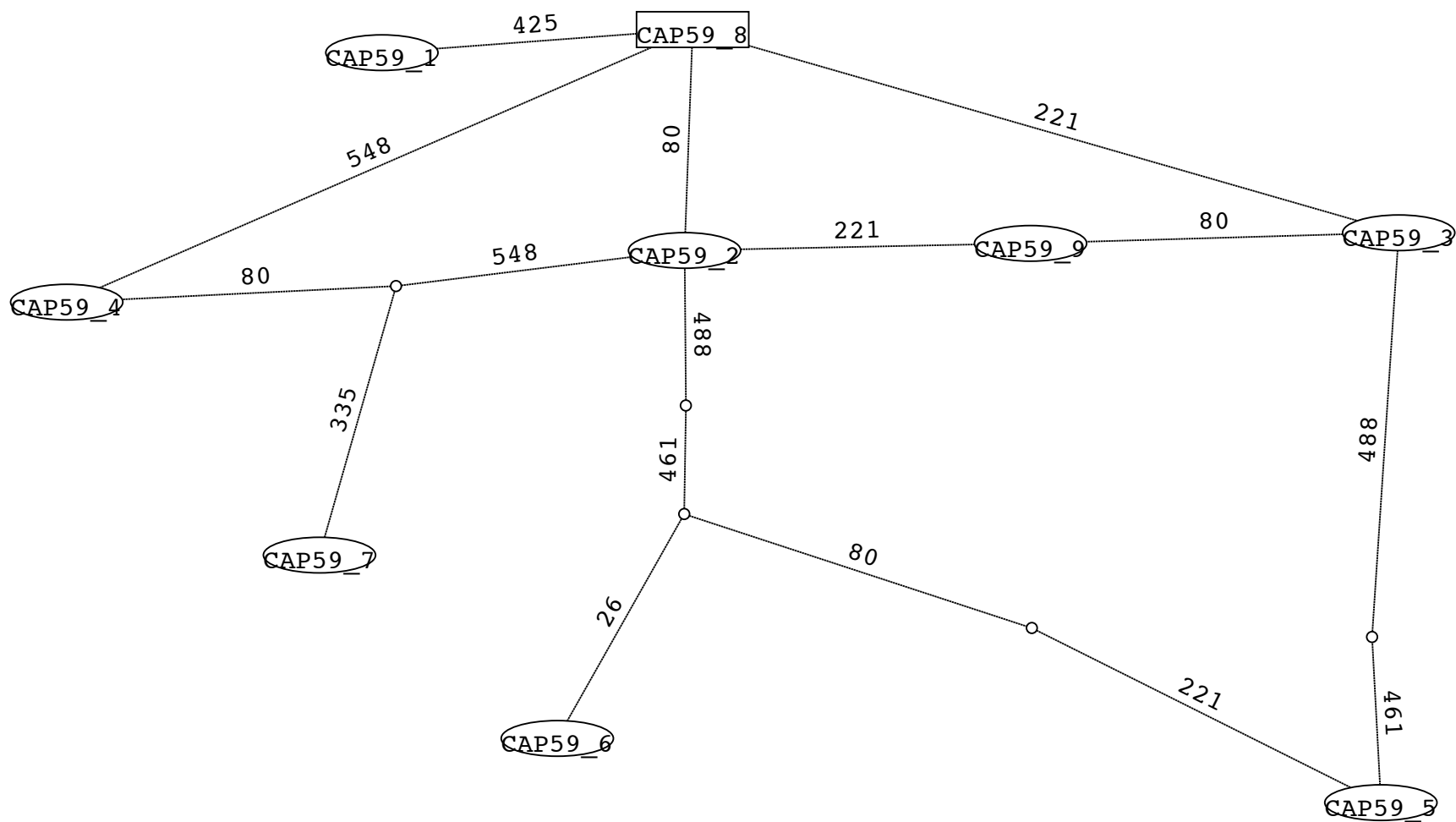

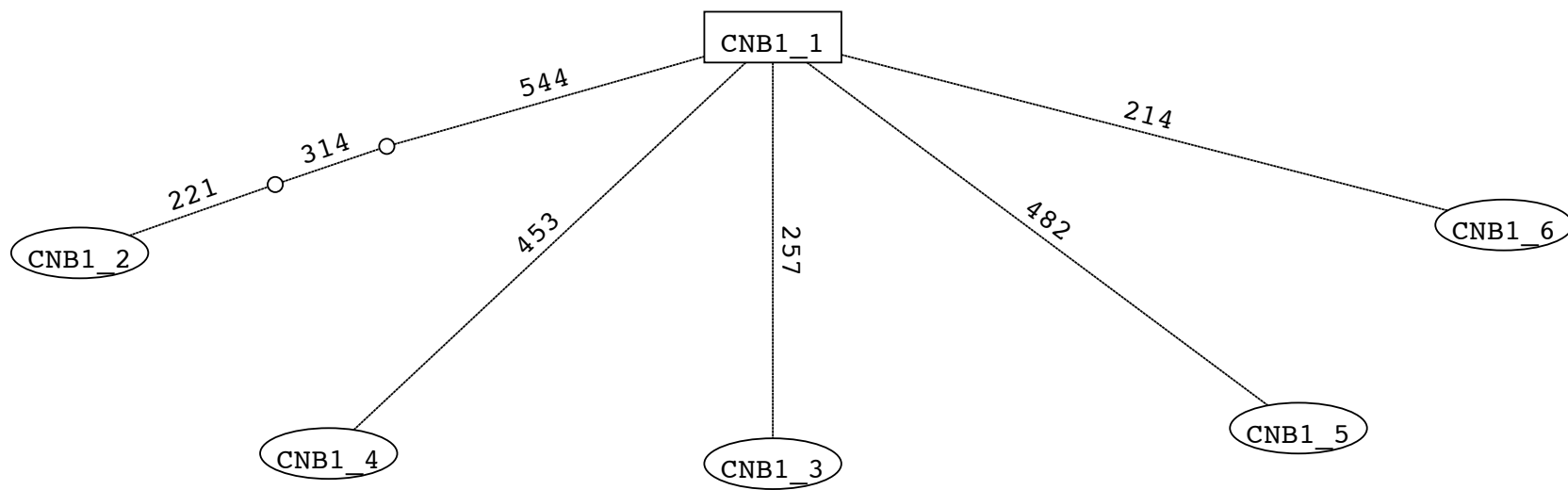

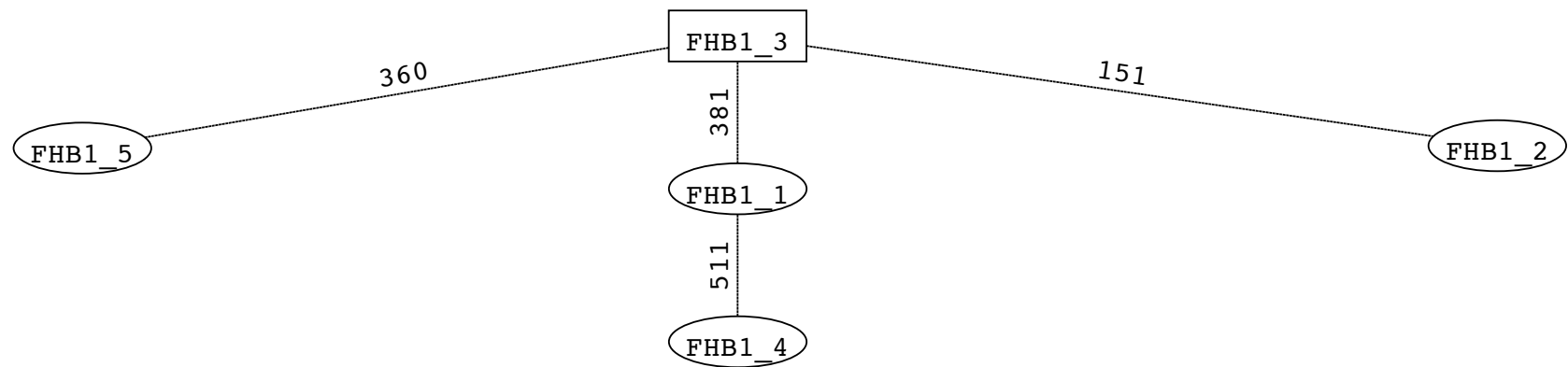

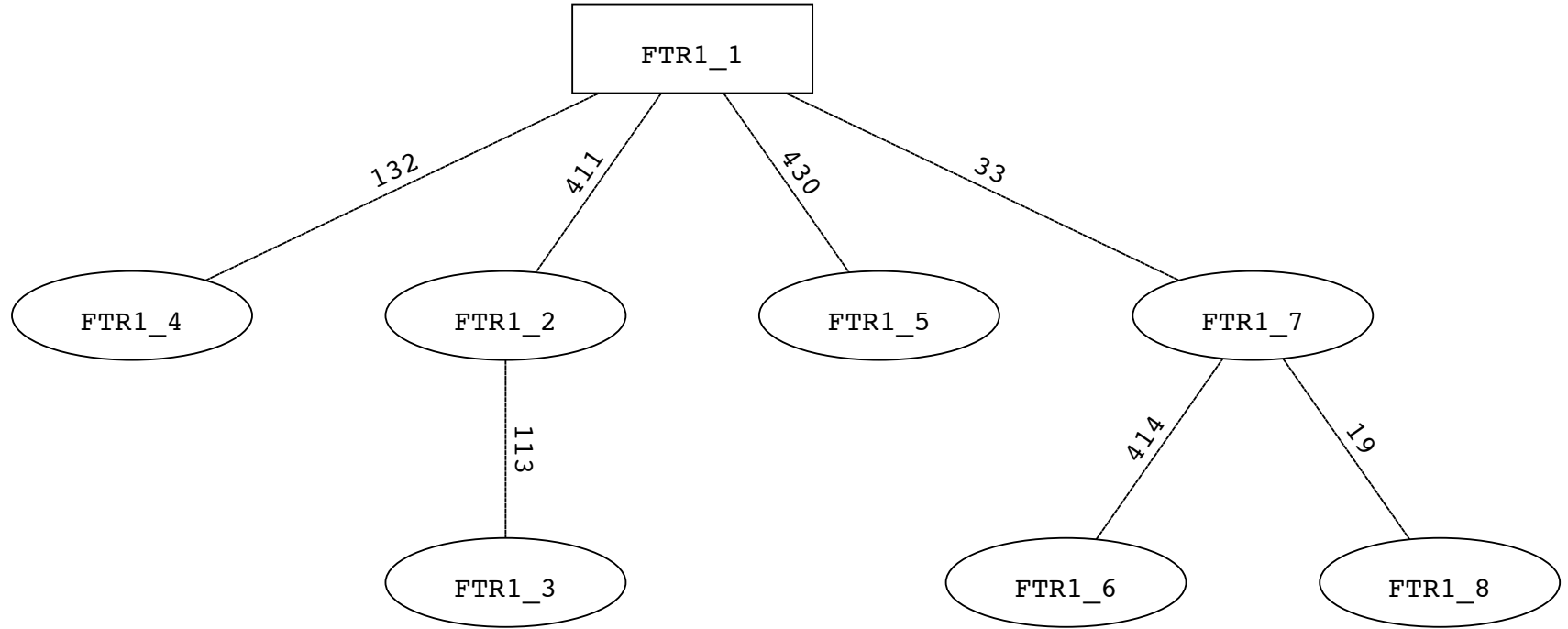

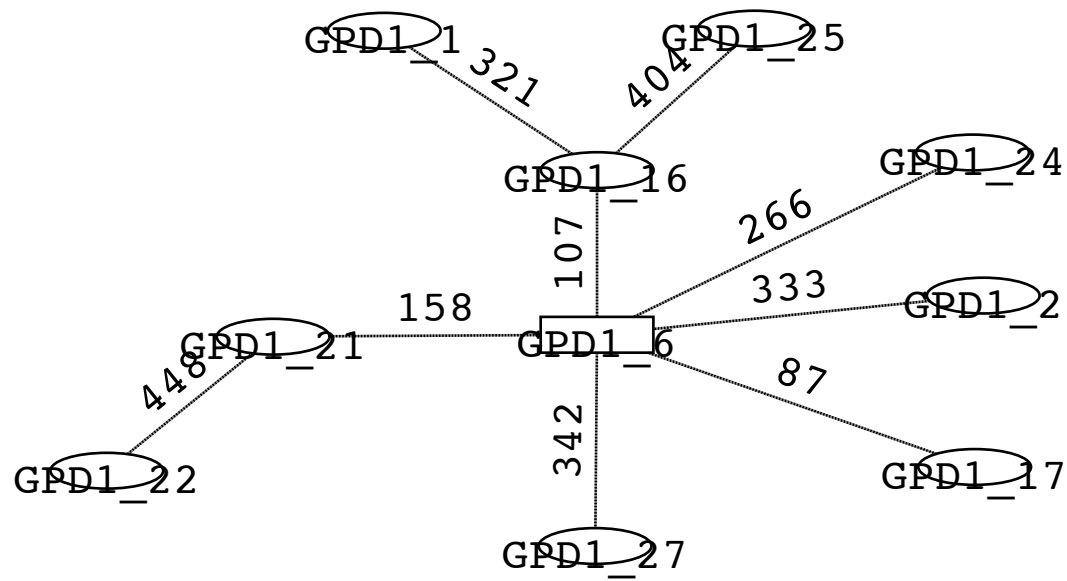

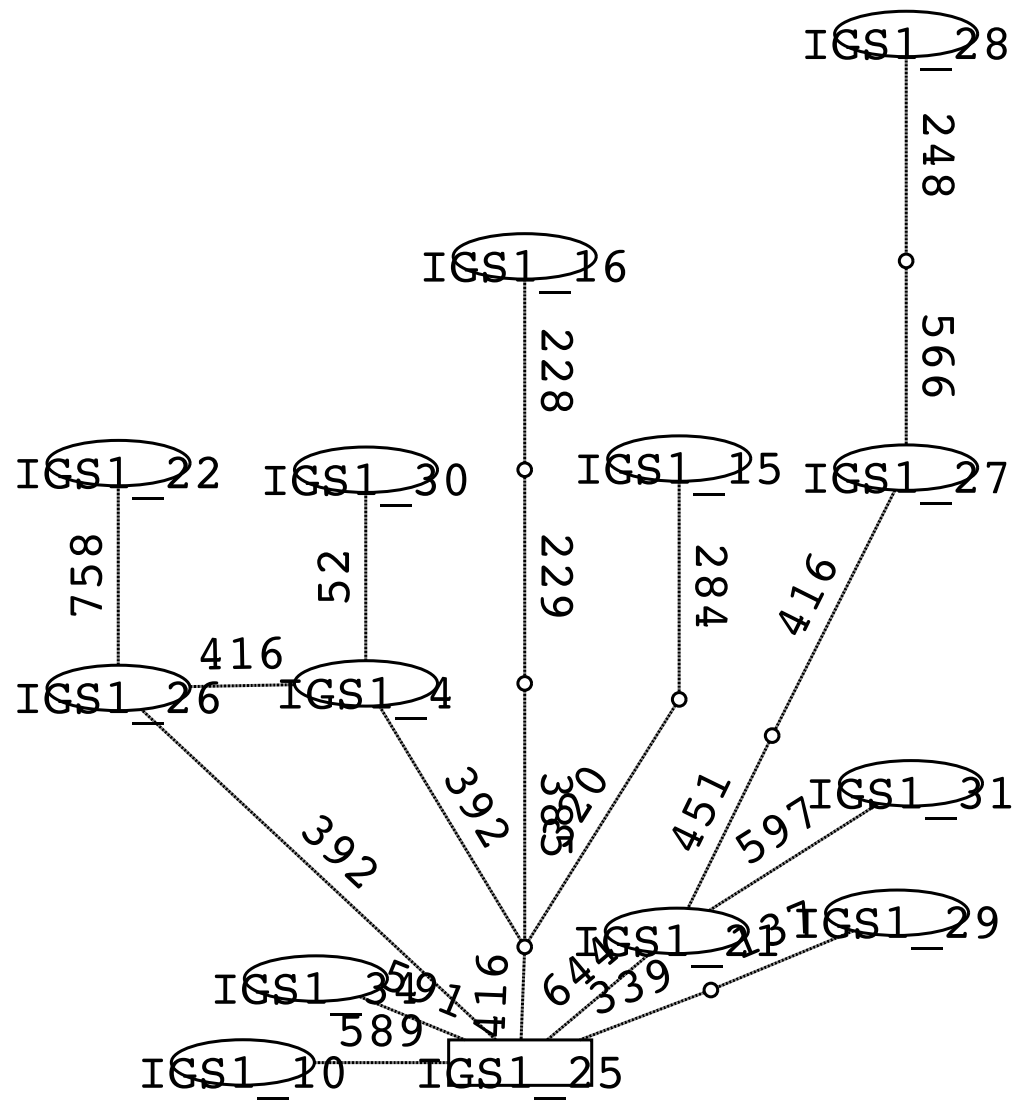

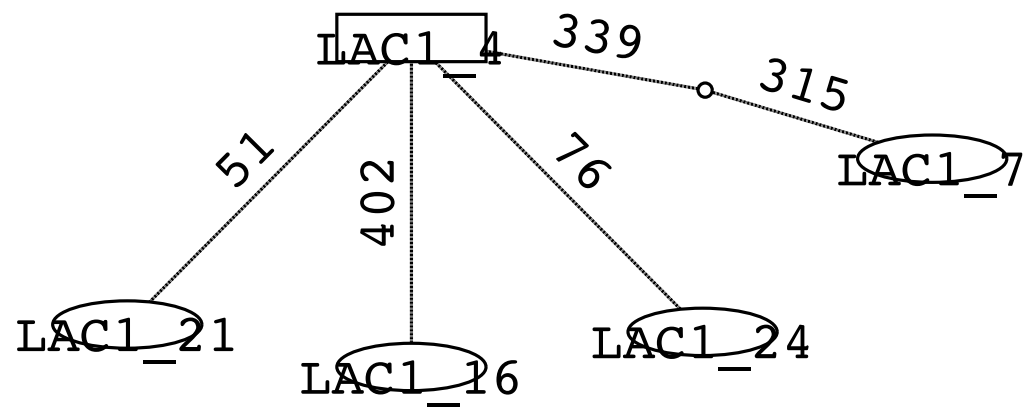

MPD1\_5

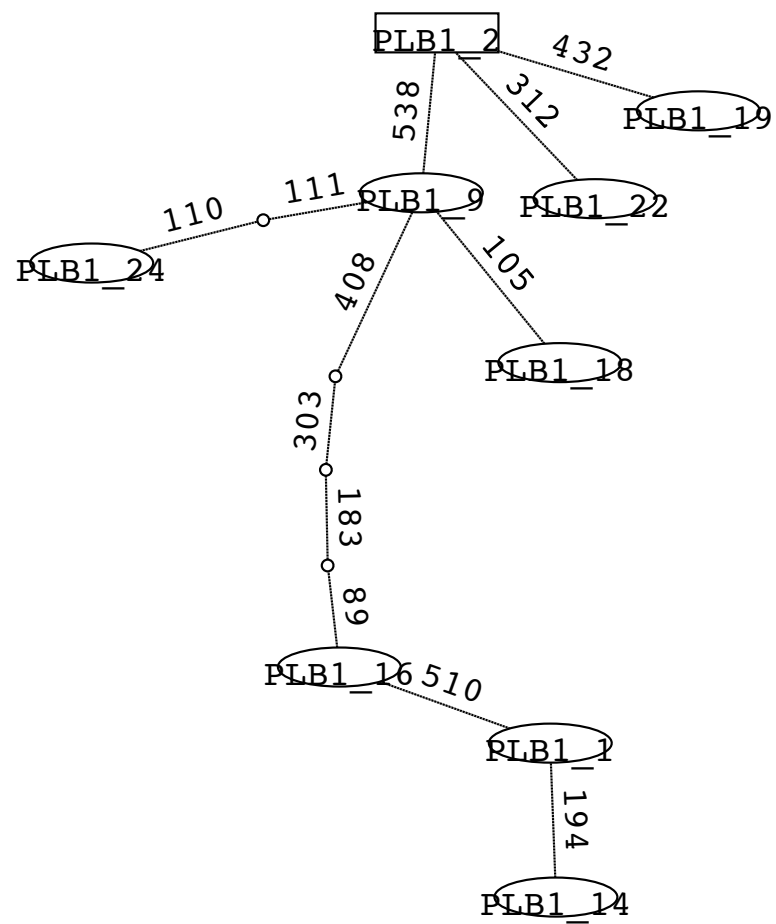

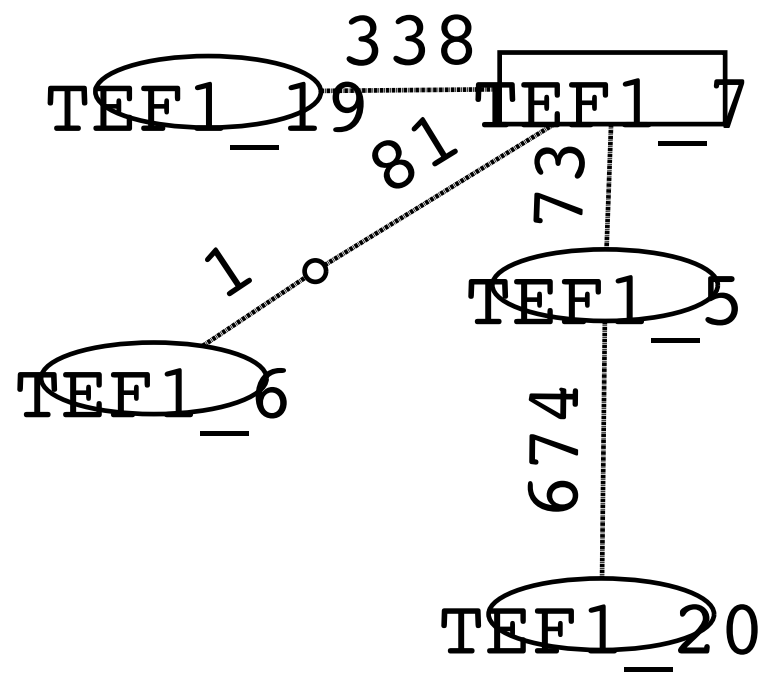

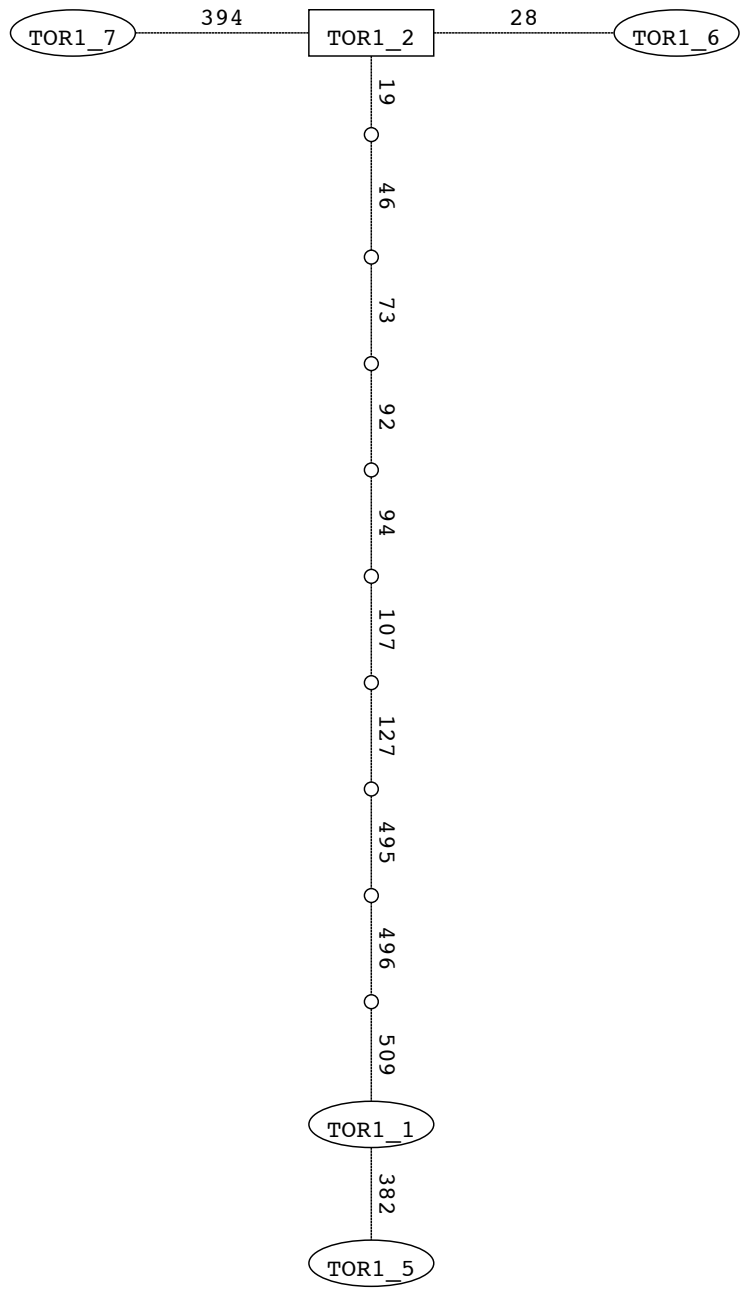

Supplement: Figure S3 — TCS haplotype networks for the thirteen alleles not represented in Figure 5 of the main text. (0.15 MB PDF) [file ppat.1000850.s003.pdf]

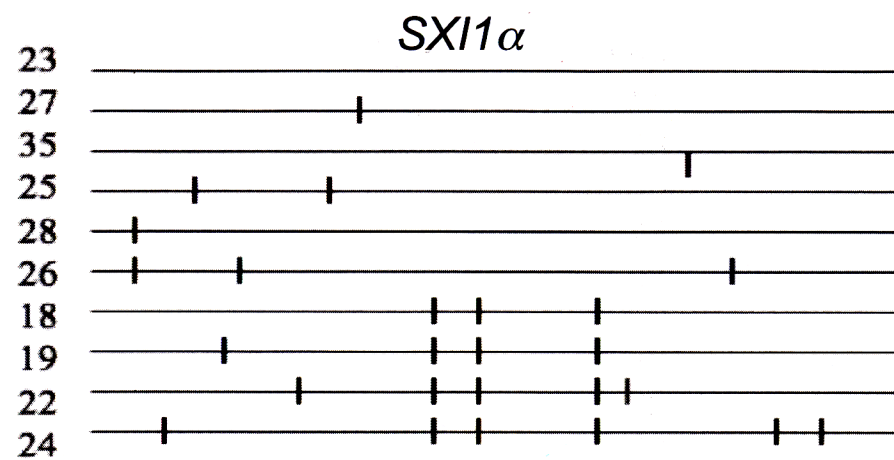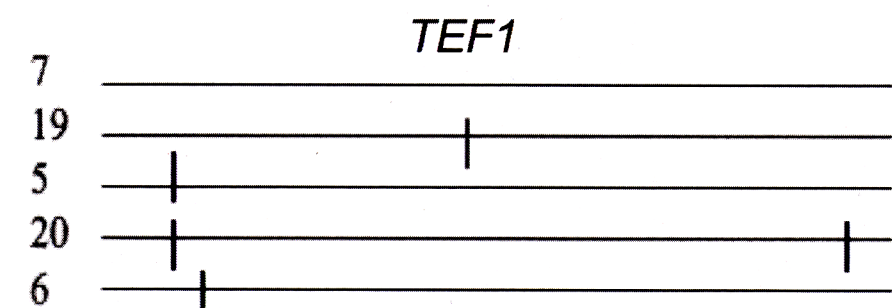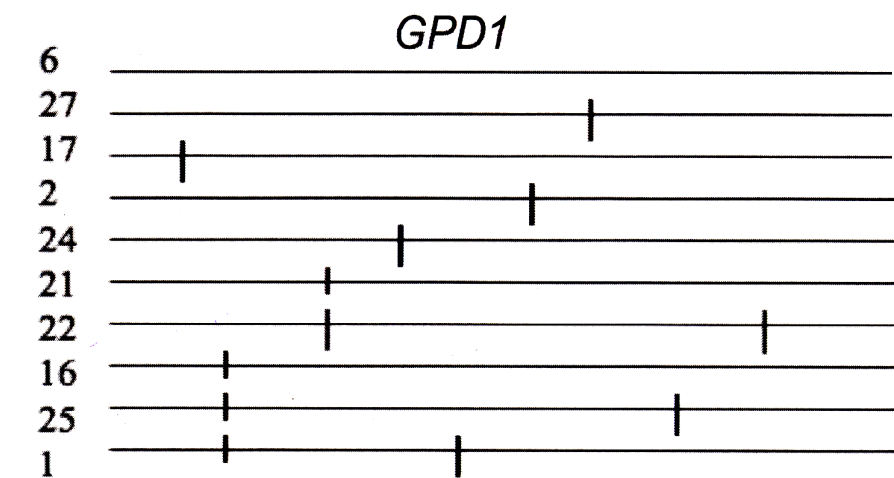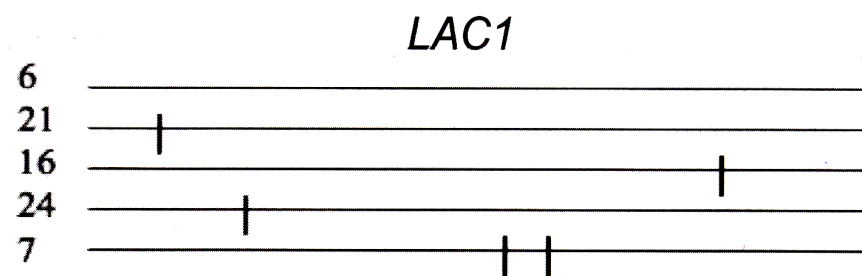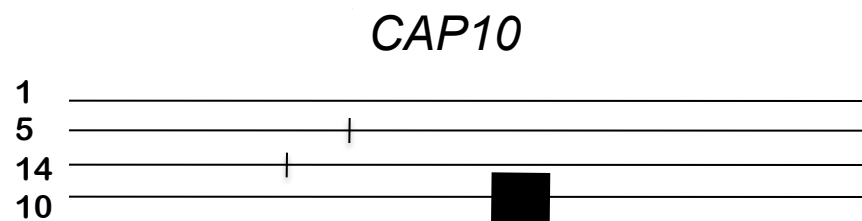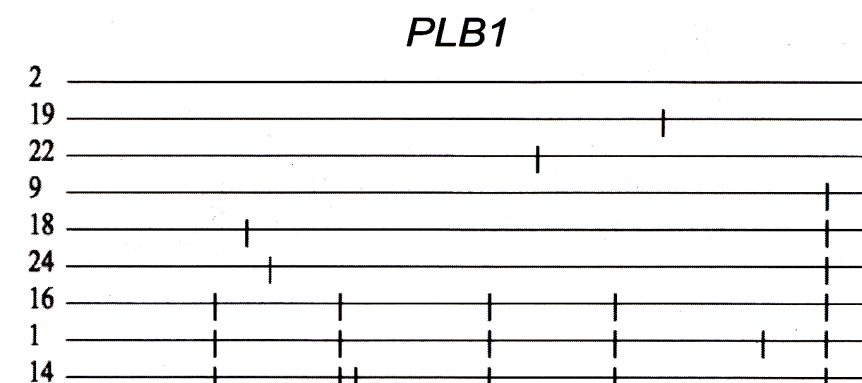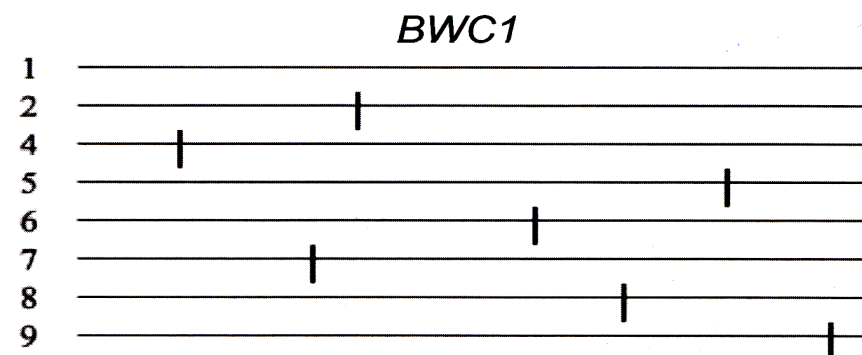

# *CNB1*

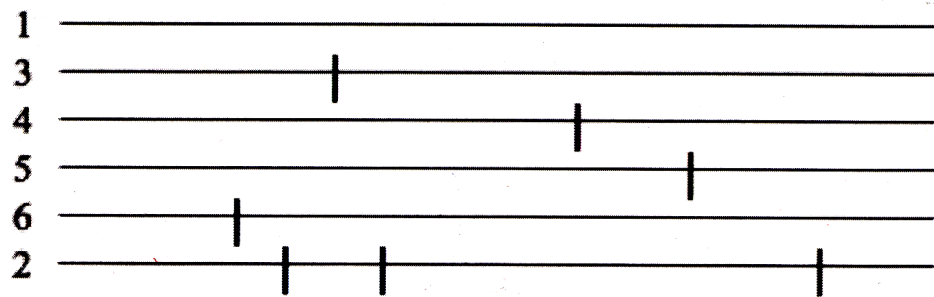

# *FHB1*

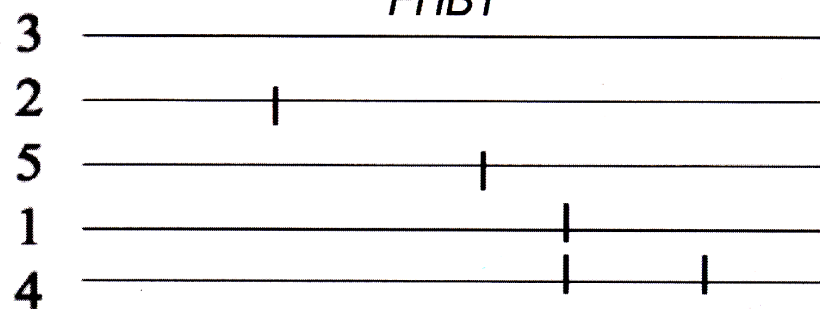

# *TOR1*

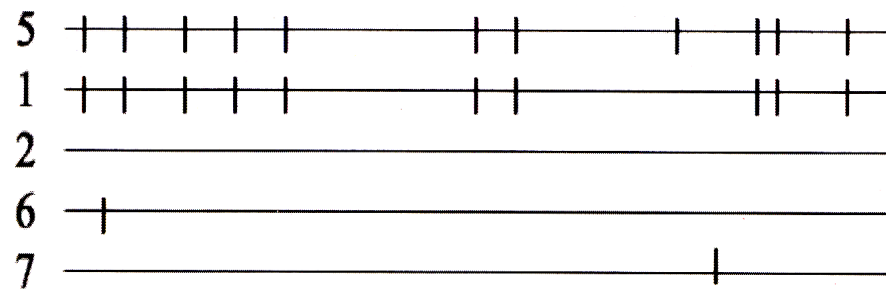

# *FTR1*

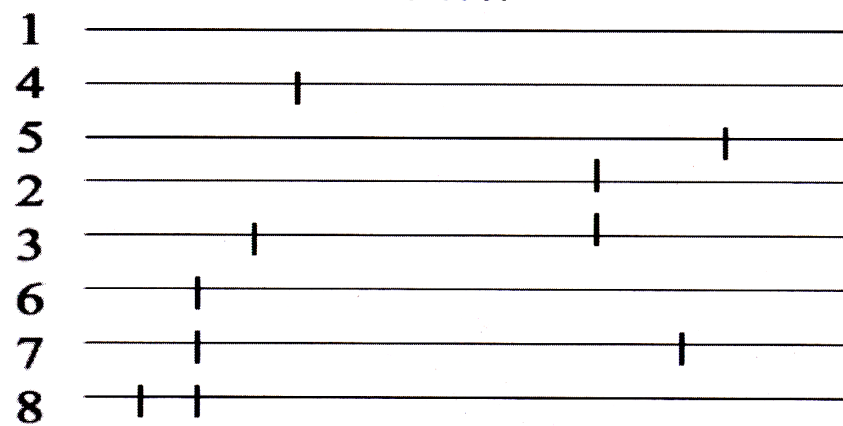

# IGS1

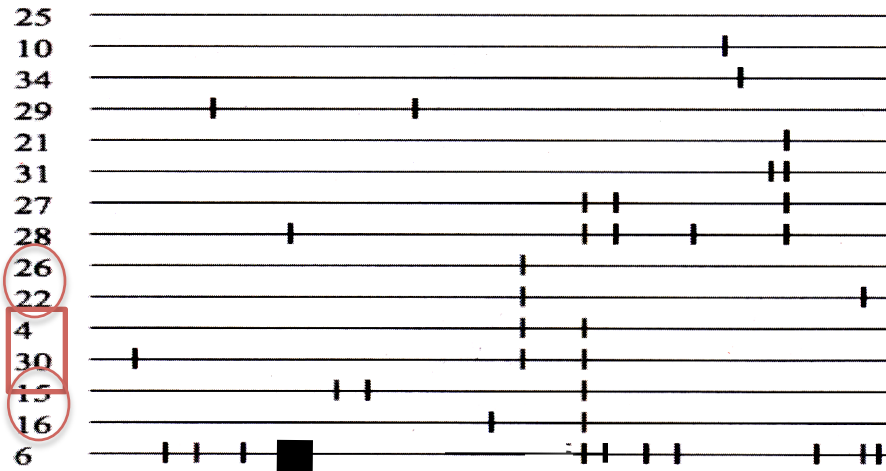

# HOG1

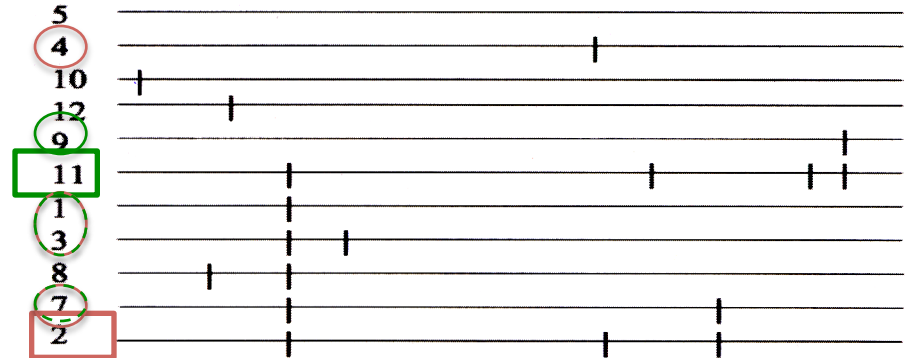

# CRG1

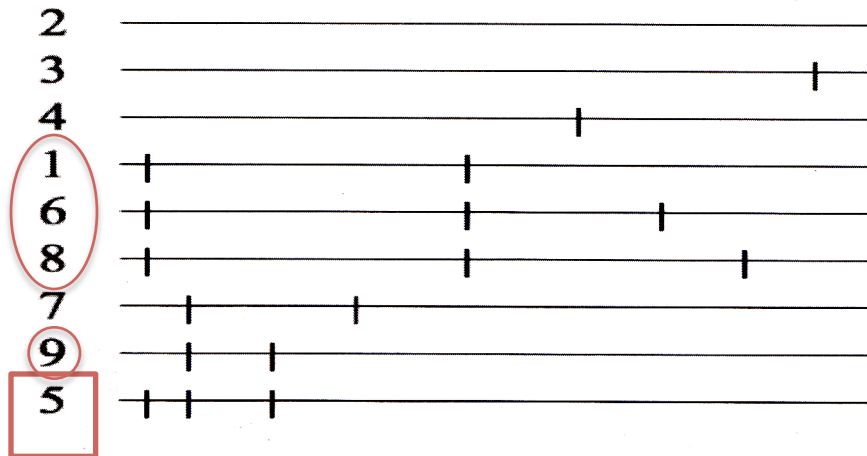

# CAP59

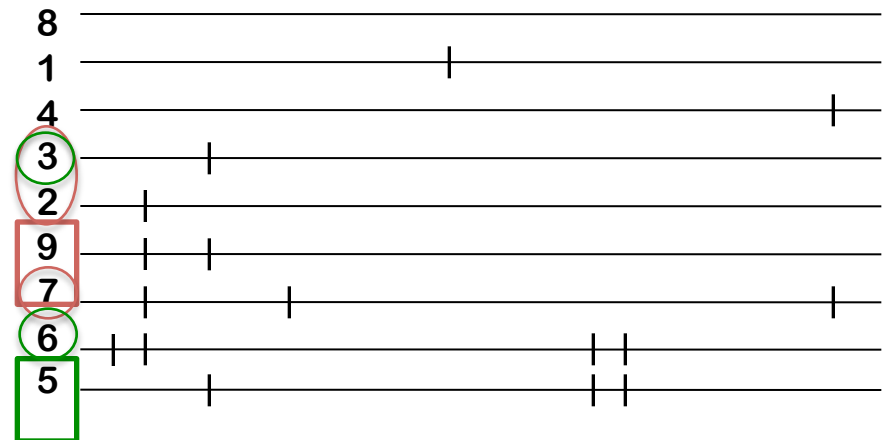

= recombinant alleles

= possible parents

Supplement: Figure S5 — Allelic recombination analysis for 15 loci indicates that 11 are likely derived from consecutive and/or independent mutations within the population. The four other loci show at least one hybrid allele that may be the result of a recombination event between two proposed parental alleles in the global VGII population. Squared alleles represent likely recombinants, while circled alleles indicate proposed parental contributors. Each of the possible contributors is indicated by a respective color. (1.34 MB PDF) [file ppat.1000850.s005.pdf]
