# Supplementary material for: Emergence and Pathogenicity of Highly Virulent Cryptococcus gattii Genotypes in the Northwest United States
Source: PLoS Pathog. 2010 Apr 22;6(4):e1000850. doi: 10.1371/journal.ppat.1000850 (PMC2858702; doi:10.1371/journal.ppat.1000850)
Supplement: Figure S4 — All paired allele graphs from VGII global isolates generated during the analysis. Isolates of both mating type a and α were included. In addition, a group of ten isolates, all α, from South America and Africa appeared most commonly as recombinant partners and are illustrated. (0.12 MB PDF) [file ppat.1000850.s004.pdf]

# *SXI v. TEF1*

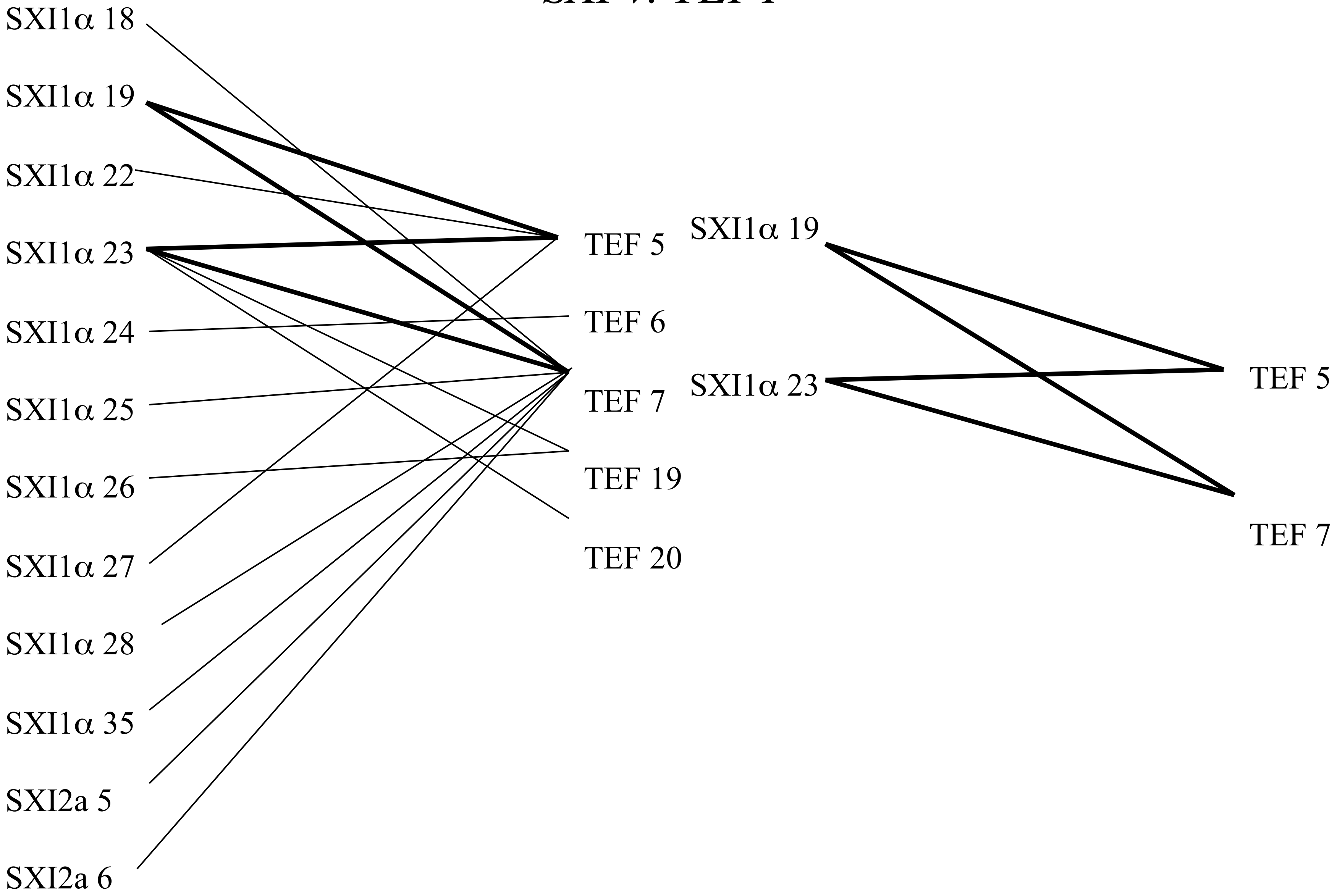
SXI1α 18
SXI1α 19
SXI1α 22
SXI1α 23
SXI1α 24
SXI1α 25
SXI1α 26
SXI1α 27
SXI1α 28
SXI1α 35
SXI2a 5
SXI2a 6
TEF 5
TEF 6
TEF 7
TEF 19
TEF 20
SXI1α 19
SXI1α 23
TEF 5
TEF 7

# *SXI v. GPD1*

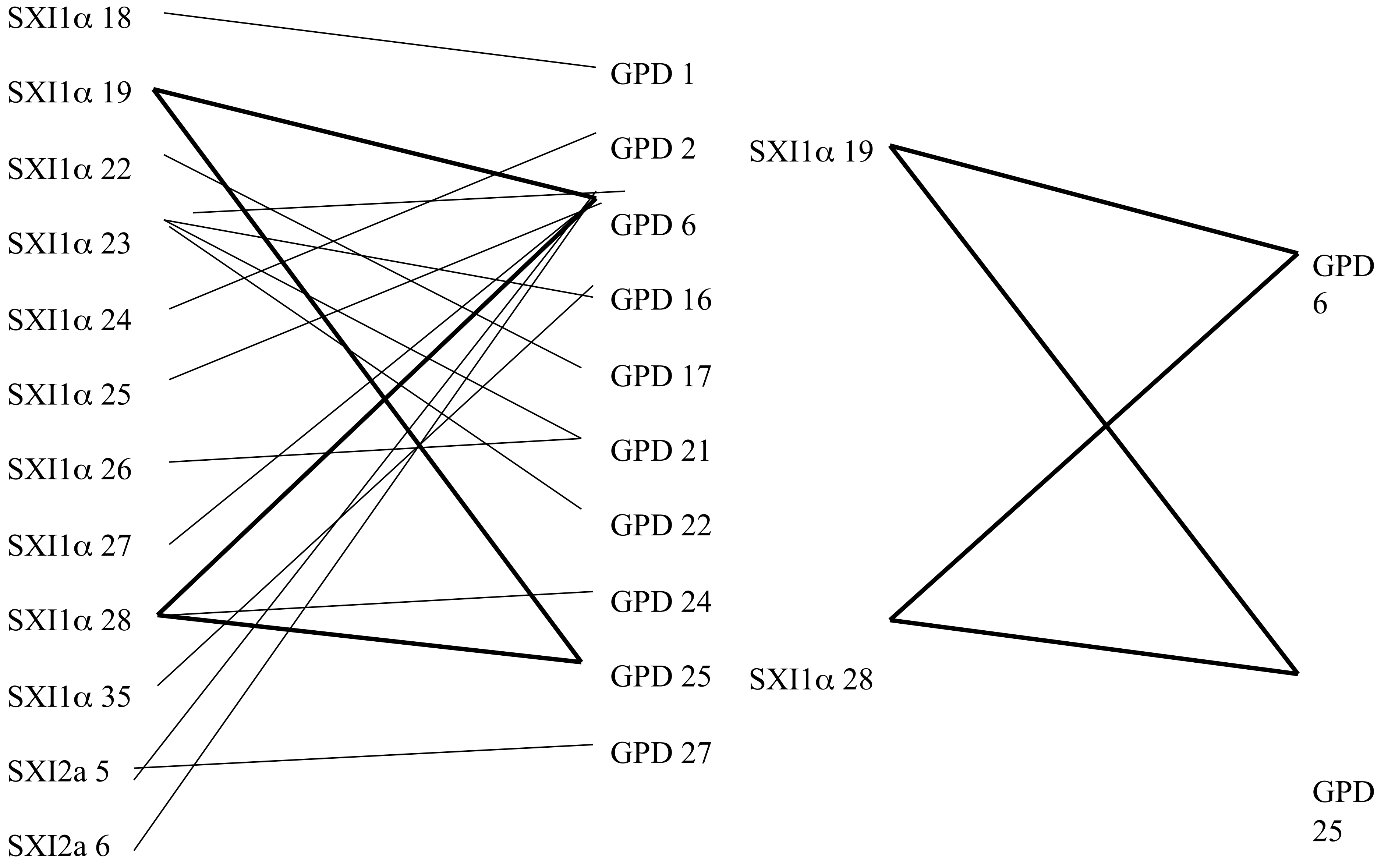
SXI1α 18
SXI1α 19
SXI1α 22
SXI1α 23
SXI1α 24
SXI1α 25
SXI1α 26
SXI1α 27
SXI1α 28
SXI1α 35
SXI2a 5
SXI2a 6
GPD 1
GPD 2
GPD 6
GPD 16
GPD 17
GPD 21
GPD 22
GPD 24
GPD 25
GPD 27
SXI1α 19
SXI1α 28
GPD 6
GPD 25

# *SXI v. LAC1*

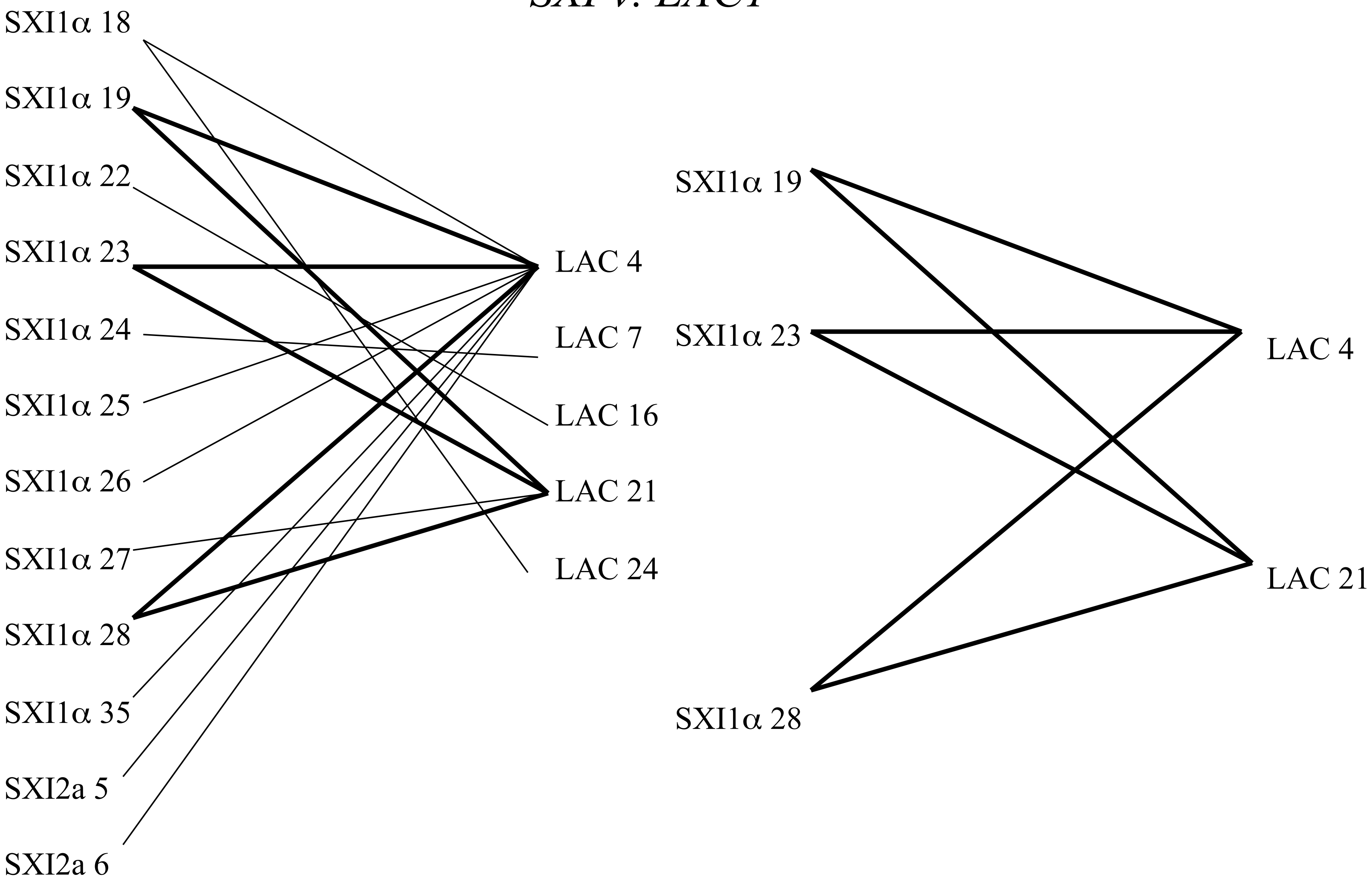
SXI1α 18
SXI1α 19
SXI1α 22
SXI1α 23
SXI1α 24
SXI1α 25
SXI1α 26
SXI1α 27
SXI1α 28
SXI1α 35
SXI2a 5
SXI2a 6
LAC 4
LAC 7
LAC 16
LAC 21
LAC 24
SXI1α 19
SXI1α 23
SXI1α 28
LAC 4
LAC 21

*SXI v. CAP10*

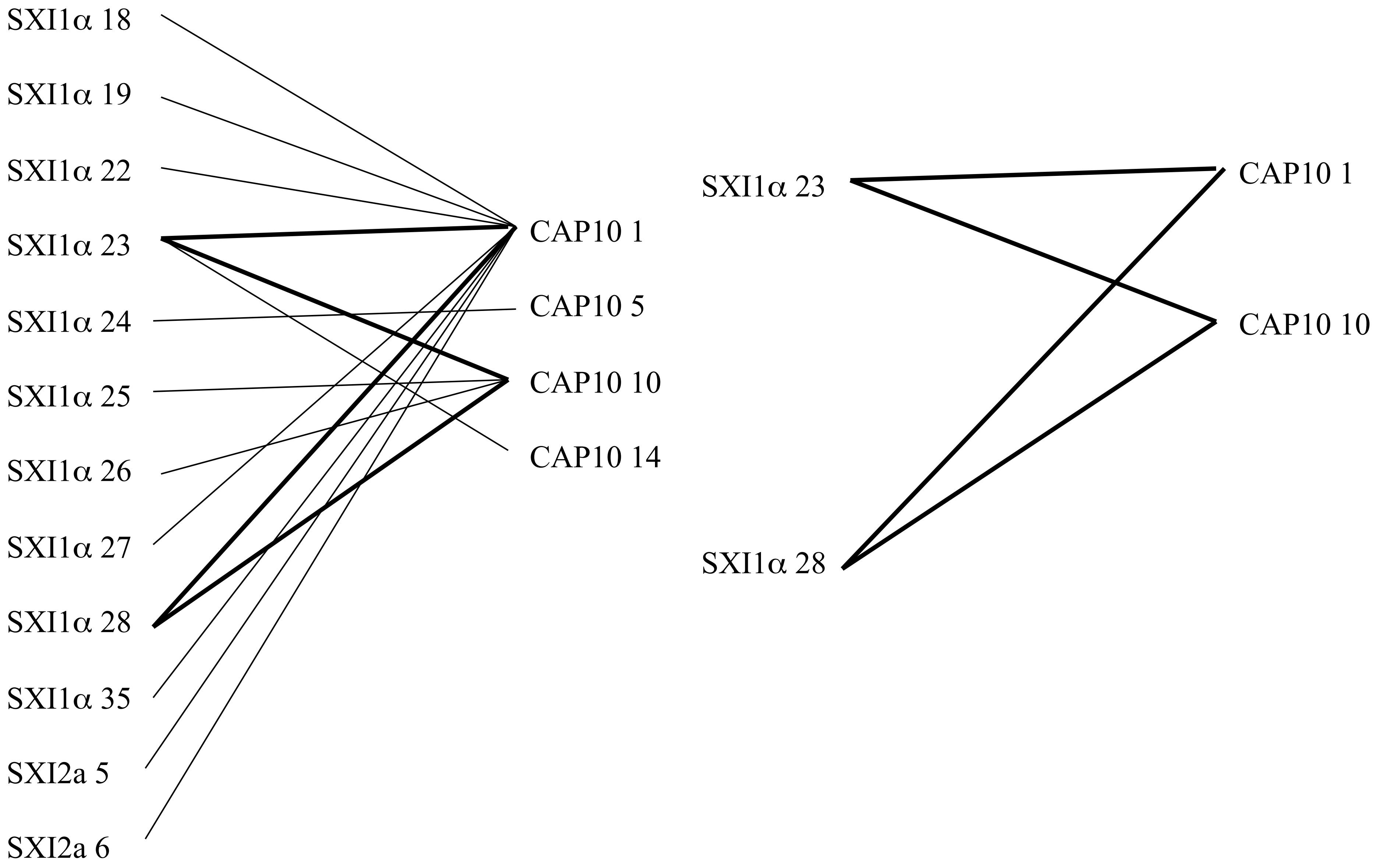
SXI1α 18
SXI1α 19
SXI1α 22
SXI1α 23
SXI1α 24
SXI1α 25
SXI1α 26
SXI1α 27
SXI1α 28
SXI1α 35
SXI2a 5
SXI2a 6
CAP10 1
CAP10 5
CAP10 10
CAP10 14
SXI1α 23
SXI1α 28
CAP10 1
CAP10 10

*SXI v. PLB1*

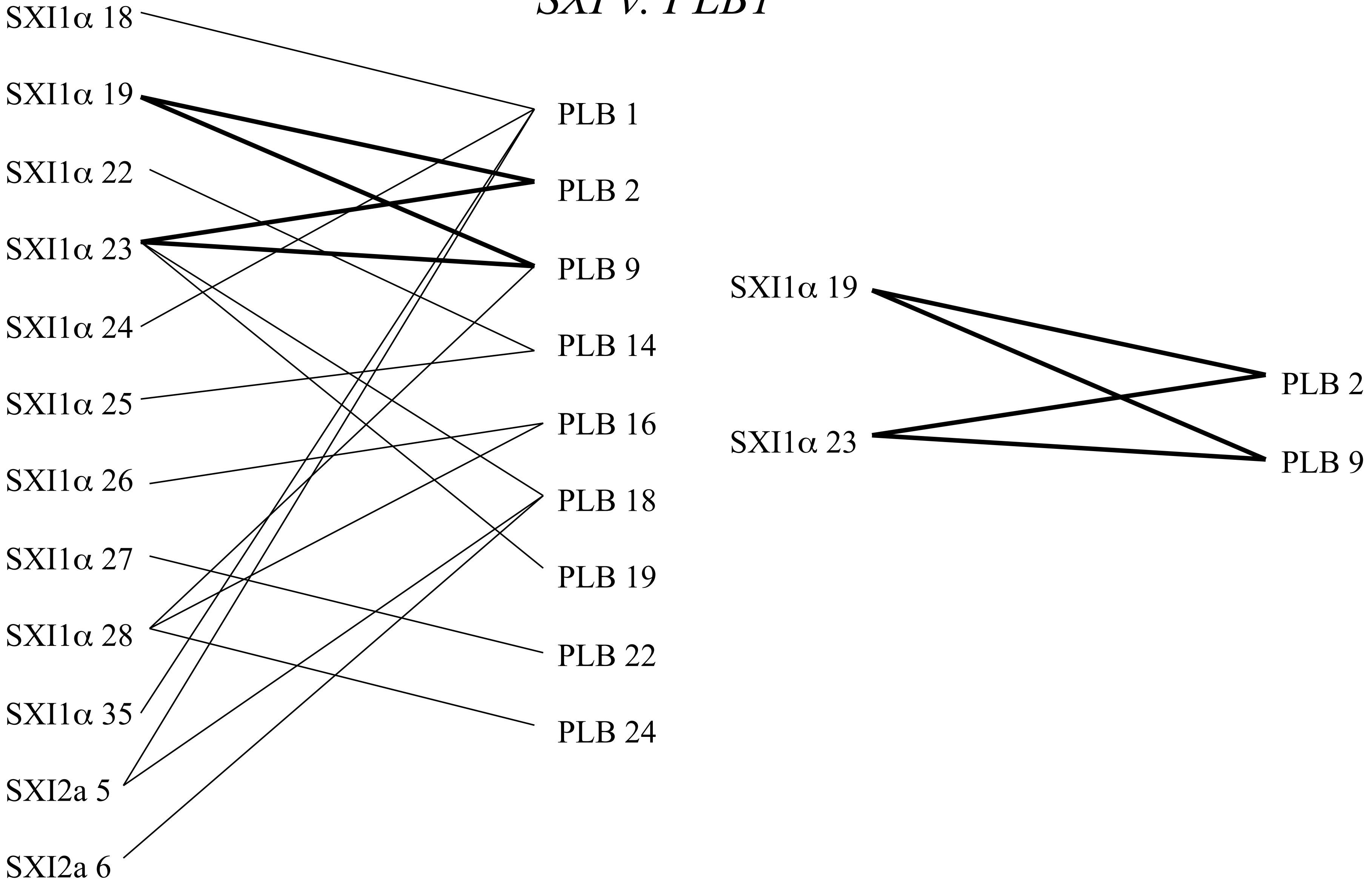
SXI1α 18
SXI1α 19
SXI1α 22
SXI1α 23
SXI1α 24
SXI1α 25
SXI1α 26
SXI1α 27
SXI1α 28
SXI1α 35
SXI2a 5
SXI2a 6
PLB 1
PLB 2
PLB 9
PLB 14
PLB 16
PLB 18
PLB 19
PLB 22
PLB 24
SXI1α 19
SXI1α 23
PLB 2
PLB 9

# *SXI v. HOG1*

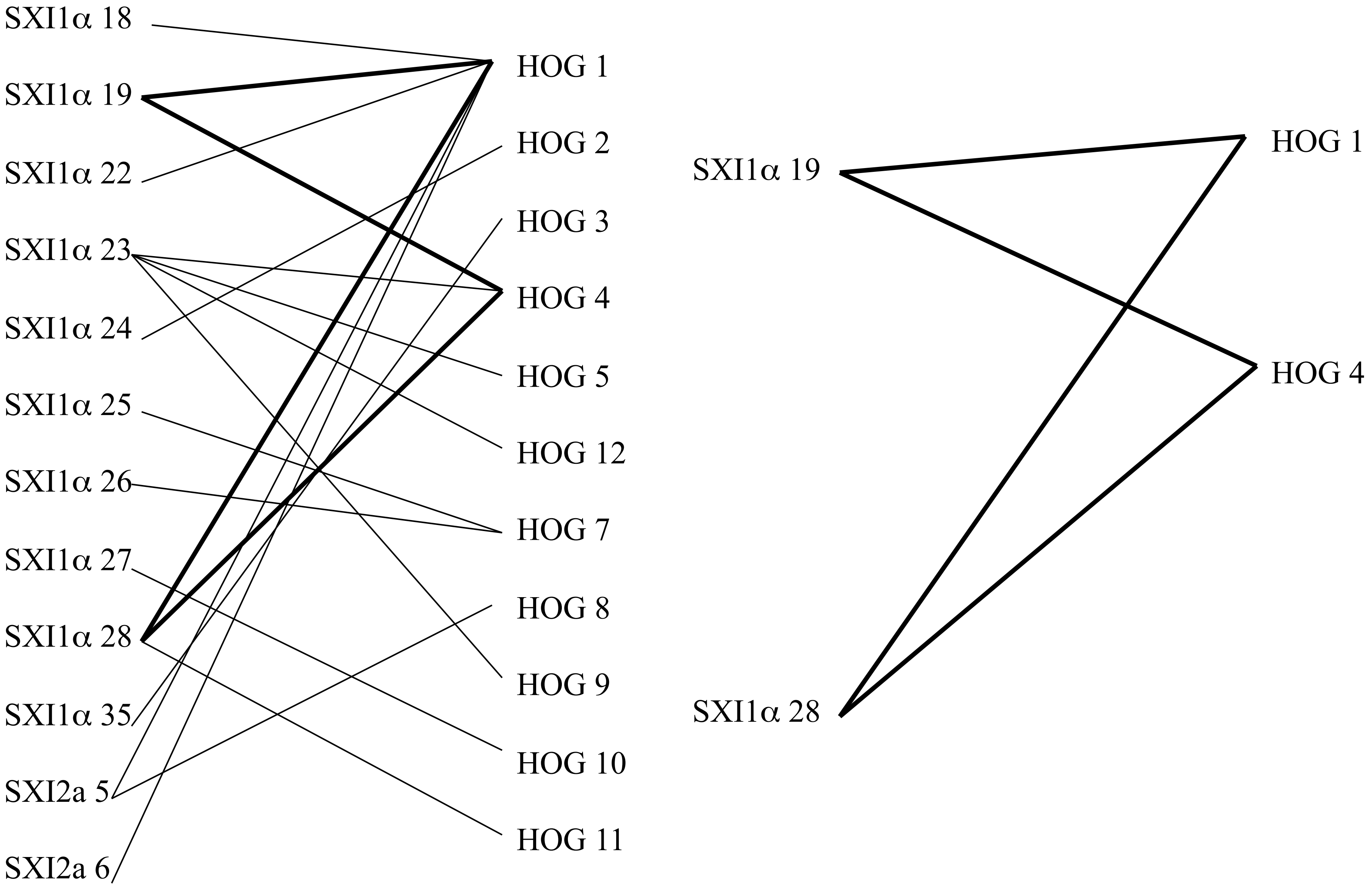

SXI1α 18
SXI1α 19
SXI1α 22
SXI1α 23
SXI1α 24
SXI1α 25
SXI1α 26
SXI1α 27
SXI1α 28
SXI1α 35
SXI2a 5
SXI2a 6
HOG 1
HOG 2
HOG 3
HOG 4
HOG 5
HOG 12
HOG 7
HOG 8
HOG 9
HOG 10
HOG 11
SXI1α 19
SXI1α 28
HOG 1
HOG 4

# *SXI v. FHB1*

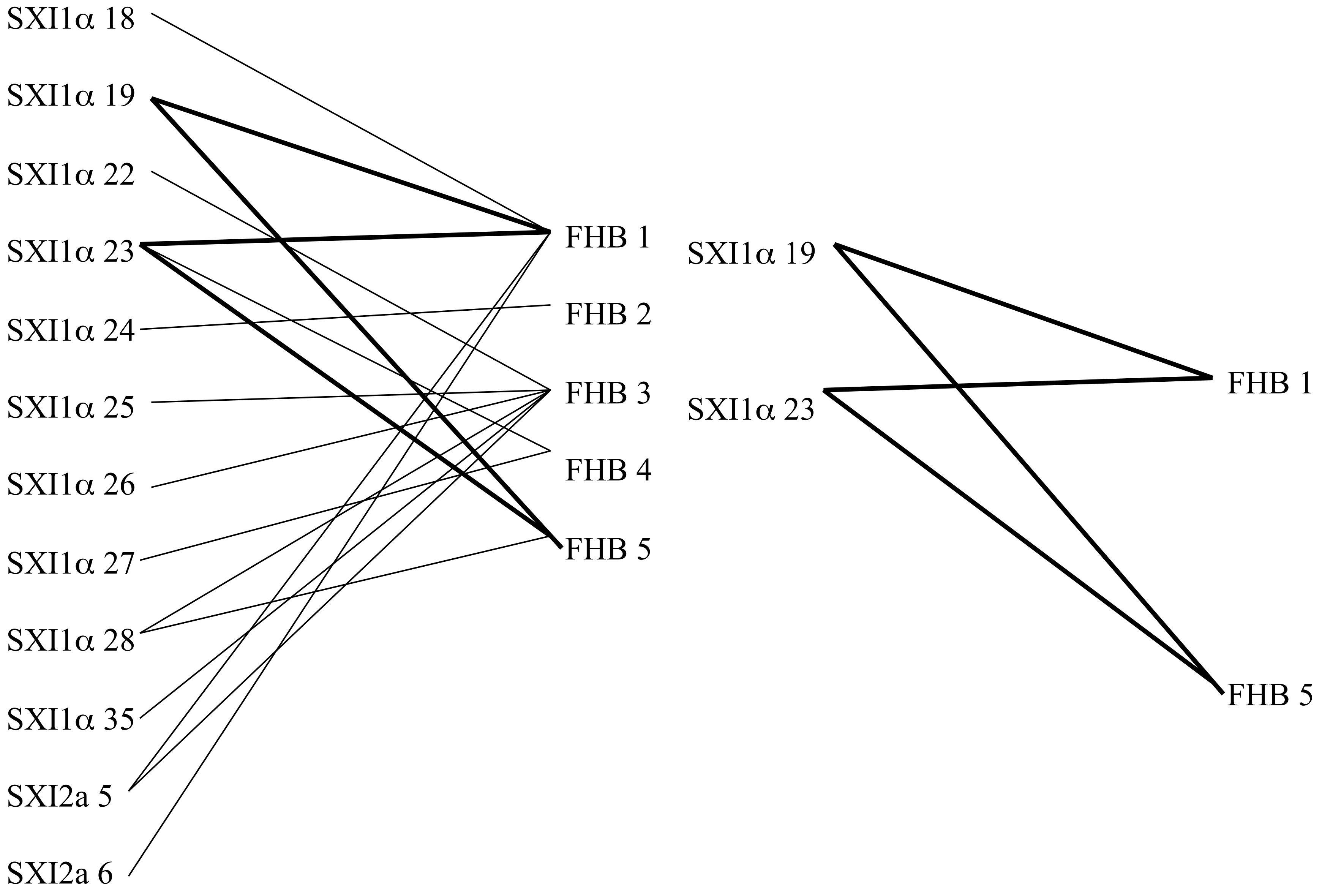
SXI1α 18
SXI1α 19
SXI1α 22
SXI1α 23
SXI1α 24
SXI1α 25
SXI1α 26
SXI1α 27
SXI1α 28
SXI1α 35
SXI2a 5
SXI2a 6
FHB 1
FHB 2
FHB 3
FHB 4
FHB 5
SXI1α 19
SXI1α 23
FHB 1
FHB 5

*IGS1 v. FHB1*

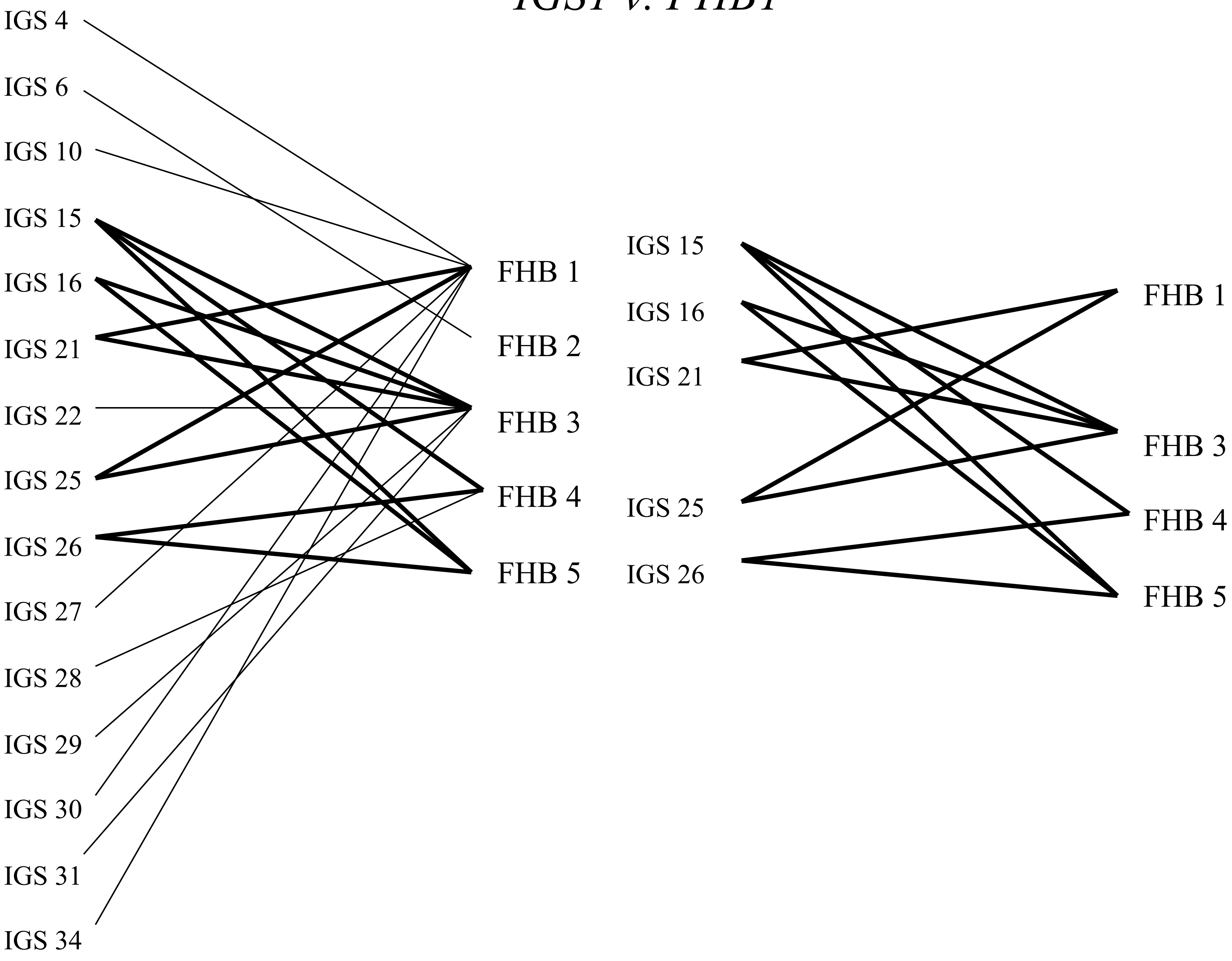

IGS 4
IGS 6
IGS 10
IGS 15
IGS 16
IGS 21
IGS 22
IGS 25
IGS 26
IGS 27
IGS 28
IGS 29
IGS 30
IGS 31
IGS 34
FHB 1
FHB 2
FHB 3
FHB 4
FHB 5
IGS 15
IGS 16
IGS 21
IGS 25
IGS 26
FHB 1
FHB 3
FHB 4
FHB 5

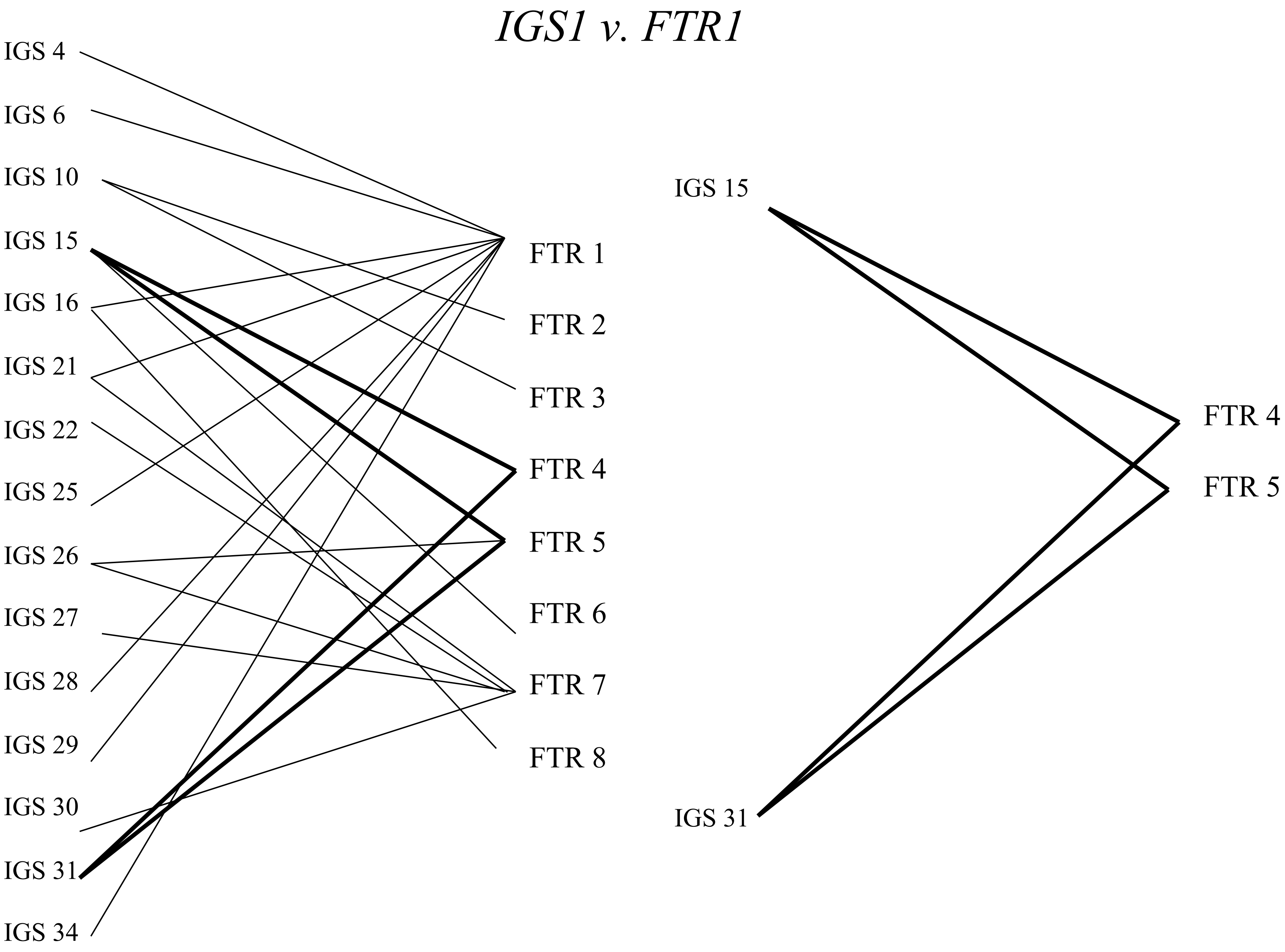
IGS1 v. FTR1
IGS 4
IGS 6
IGS 10
IGS 15
IGS 16
IGS 21
IGS 22
IGS 25
IGS 26
IGS 27
IGS 28
IGS 29
IGS 30
IGS 31
IGS 34
FTR 1
FTR 2
FTR 3
FTR 4
FTR 5
FTR 6
FTR 7
FTR 8
IGS 15
FTR 4
FTR 5
IGS 31

*TEF1 v. GPD1*

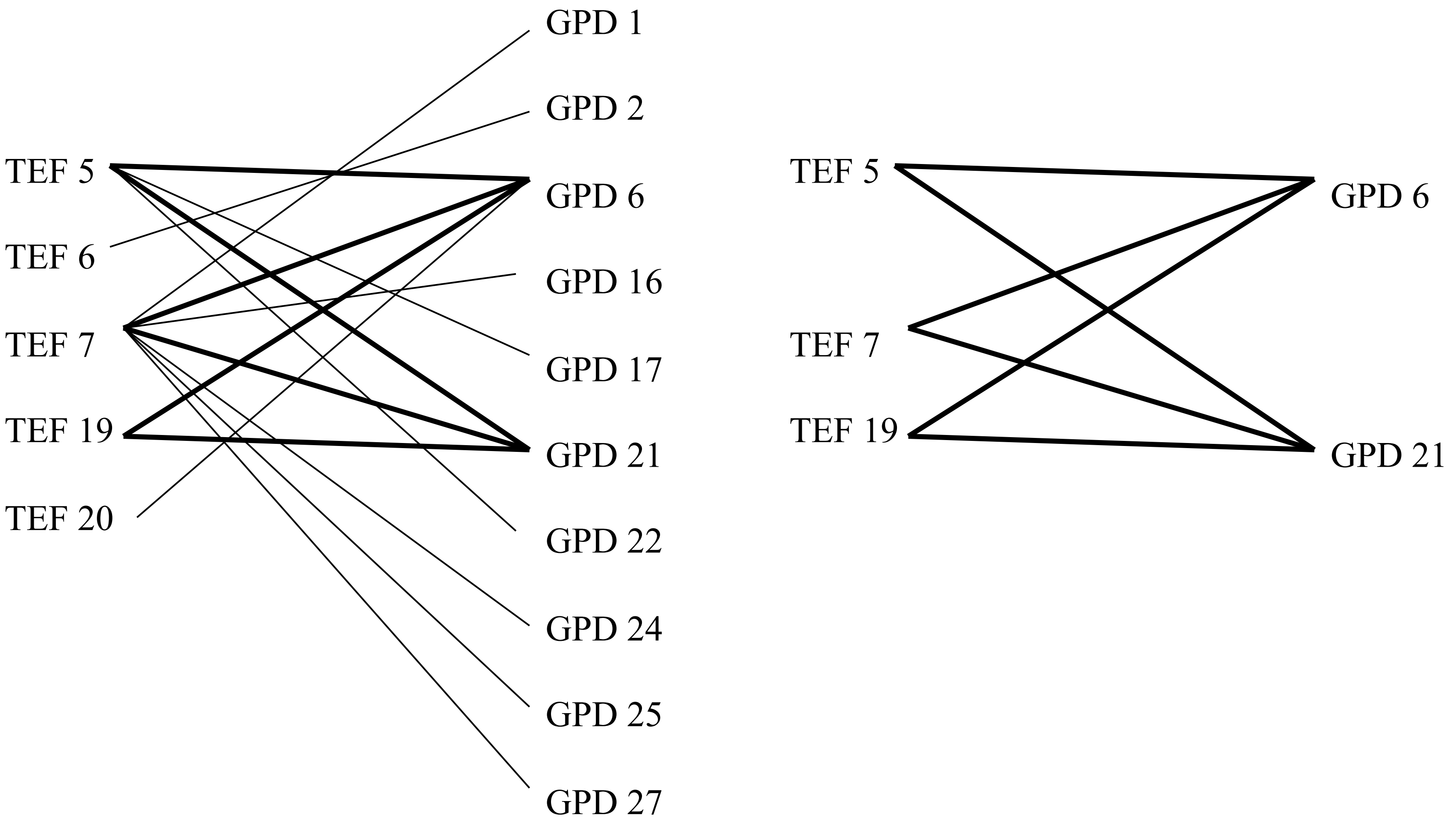

GPD 1
GPD 2
TEF 5
GPD 6
TEF 6
GPD 16
TEF 7
GPD 17
TEF 19
GPD 21
TEF 20
GPD 22
GPD 24
GPD 25
GPD 27
TEF 5
GPD 6
TEF 7
TEF 19
GPD 21

# *TEF1 v. CAP10*

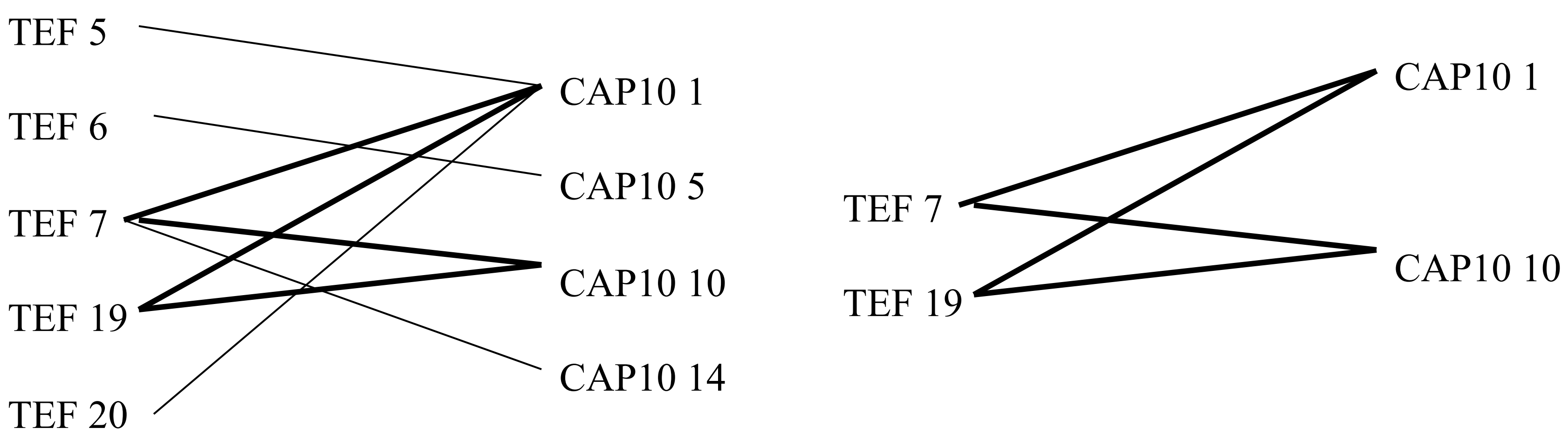

TEF 5
TEF 6
TEF 7
TEF 19
TEF 20
CAP10 1
CAP10 5
CAP10 10
CAP10 14
TEF 7
TEF 19
CAP10 1
CAP10 10

*TEF1 v. PLB1*

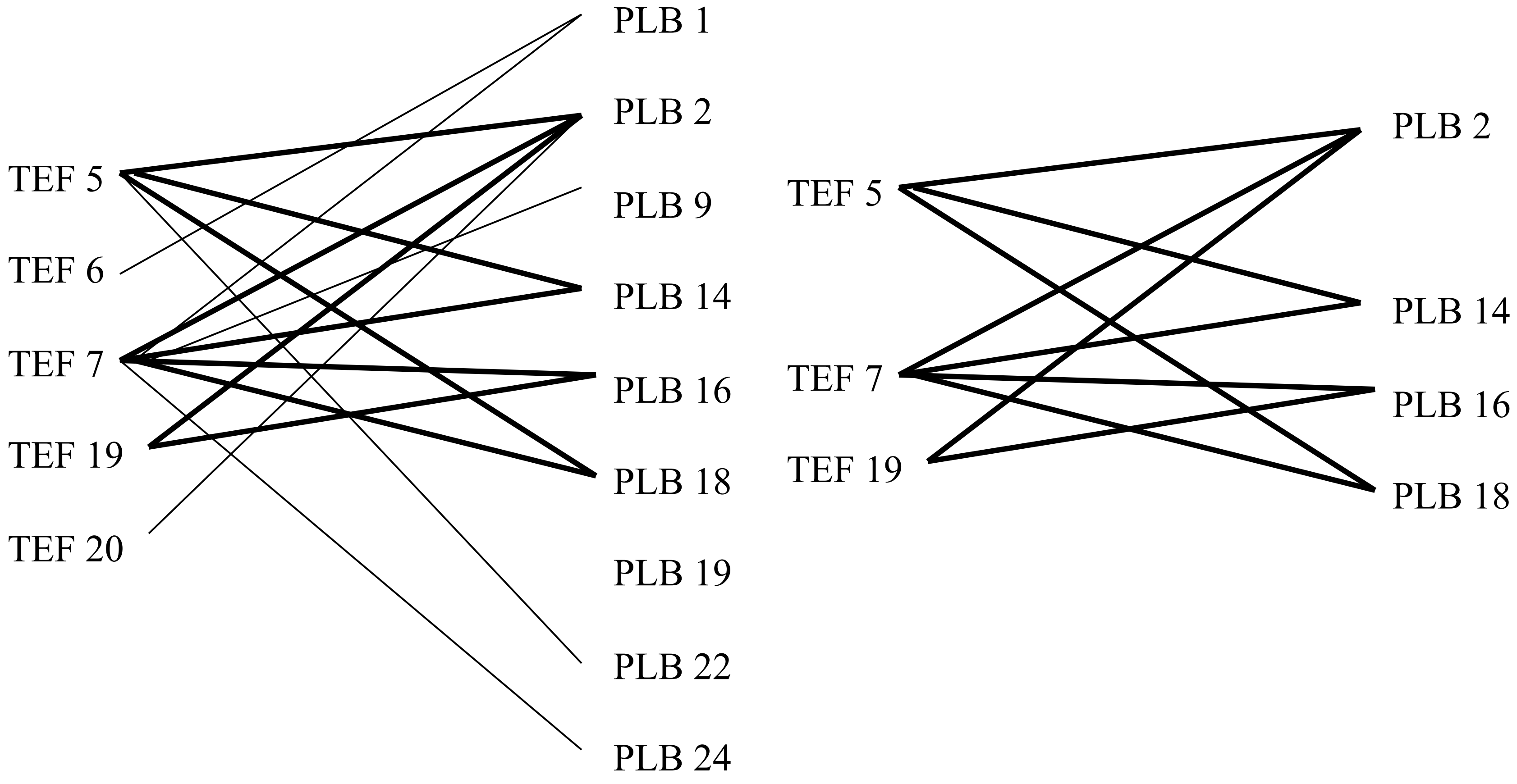
PLB 1
PLB 2
TEF 5
PLB 9
TEF 6
PLB 14
TEF 7
PLB 16
TEF 19
PLB 18
TEF 20
PLB 19
PLB 22
PLB 24
PLB 2
TEF 5
PLB 14
TEF 7
PLB 16
TEF 19
PLB 18

# *TEF1 v. HOG1*

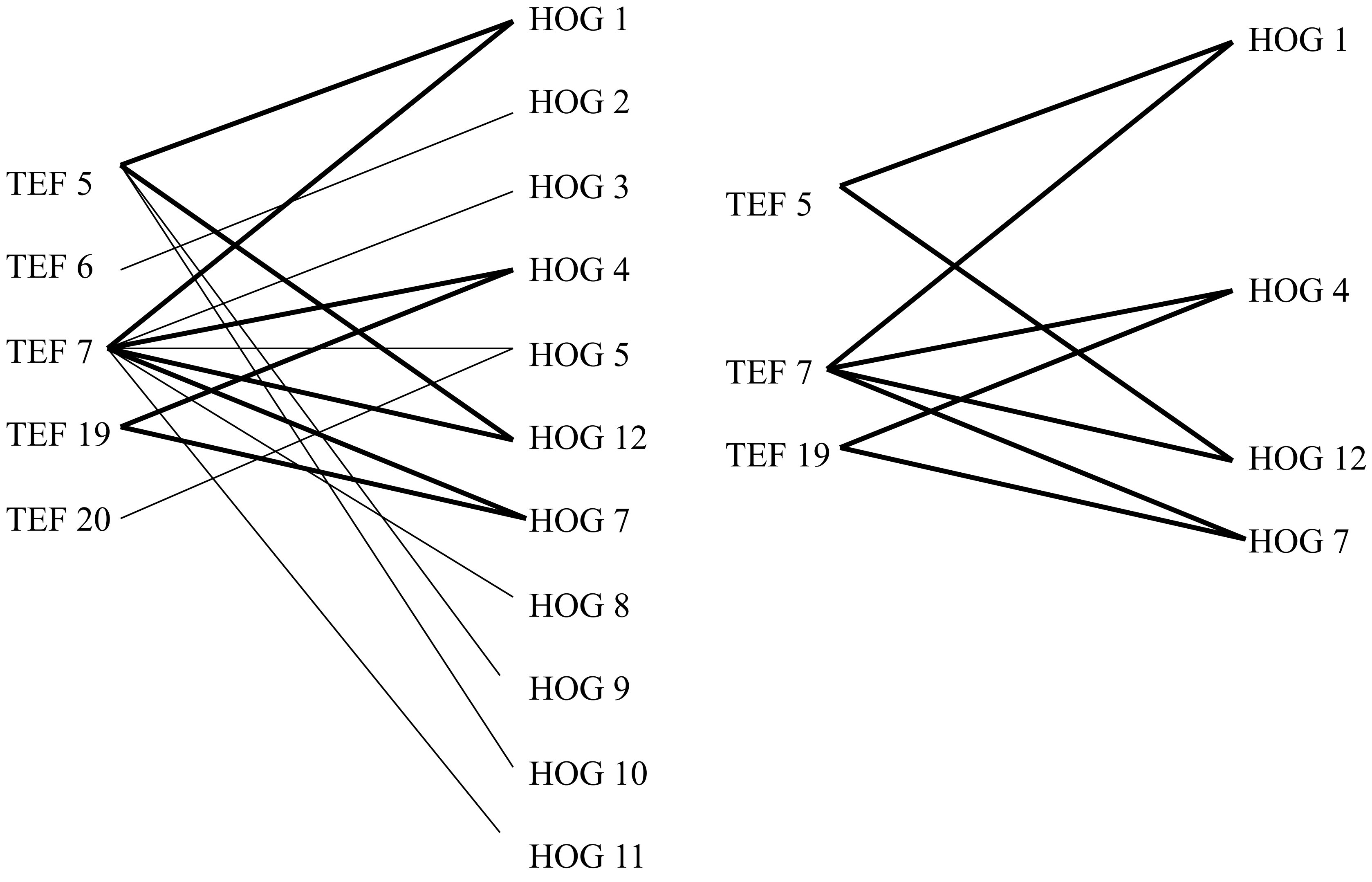
TEF 5
TEF 6
TEF 7
TEF 19
TEF 20
HOG 1
HOG 2
HOG 3
HOG 4
HOG 5
HOG 12
HOG 7
HOG 8
HOG 9
HOG 10
HOG 11
TEF 5
TEF 7
TEF 19
HOG 1
HOG 4
HOG 12
HOG 7

*TEF1 v. CNB1*

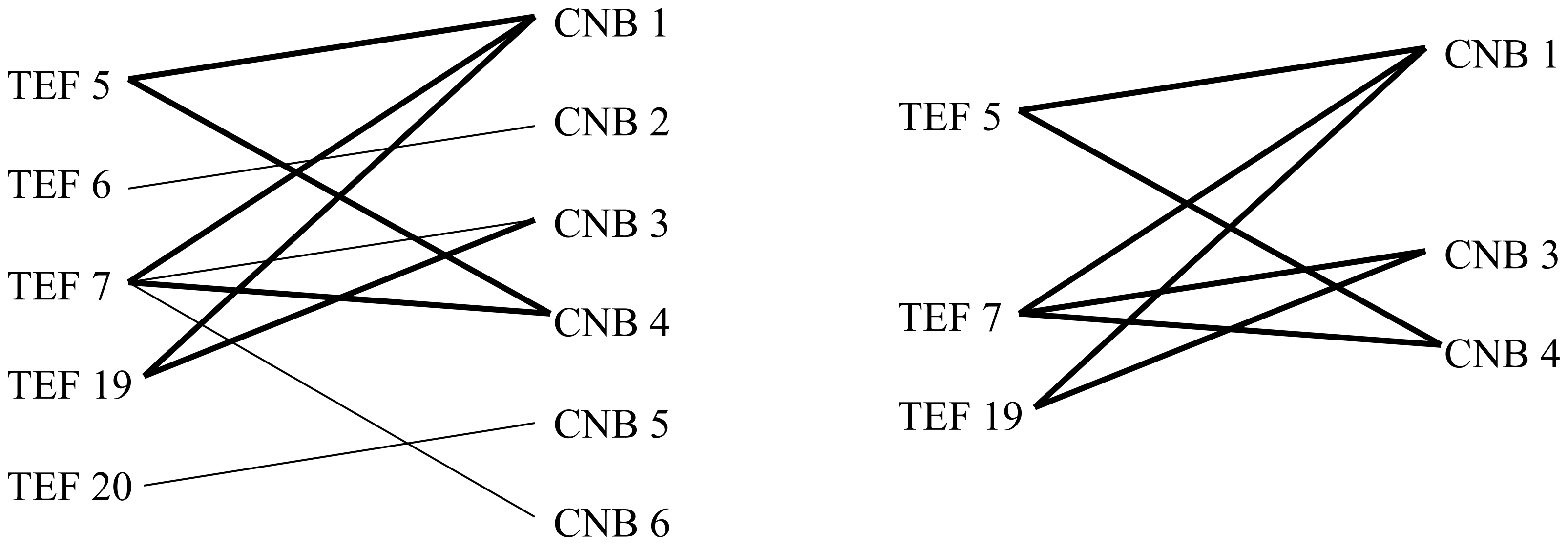
CNB 1
TEF 5
CNB 2
TEF 6
CNB 3
TEF 7
CNB 4
TEF 19
CNB 5
TEF 20
CNB 6
CNB 1
TEF 5
CNB 3
TEF 7
CNB 4
TEF 19

# *TEF1 v. TOR1*

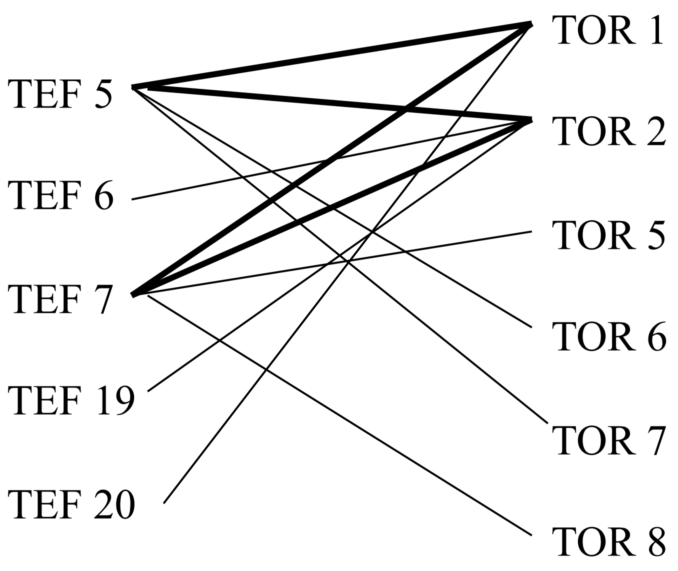

TEF 5
TEF 6
TEF 7
TEF 19
TEF 20
TOR 1
TOR 2
TOR 5
TOR 6
TOR 7
TOR 8


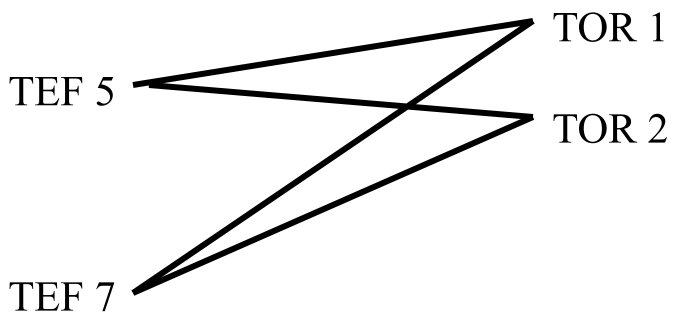

TEF 5
TEF 7
TOR 1
TOR 2

*TEF1 v. CRG1*

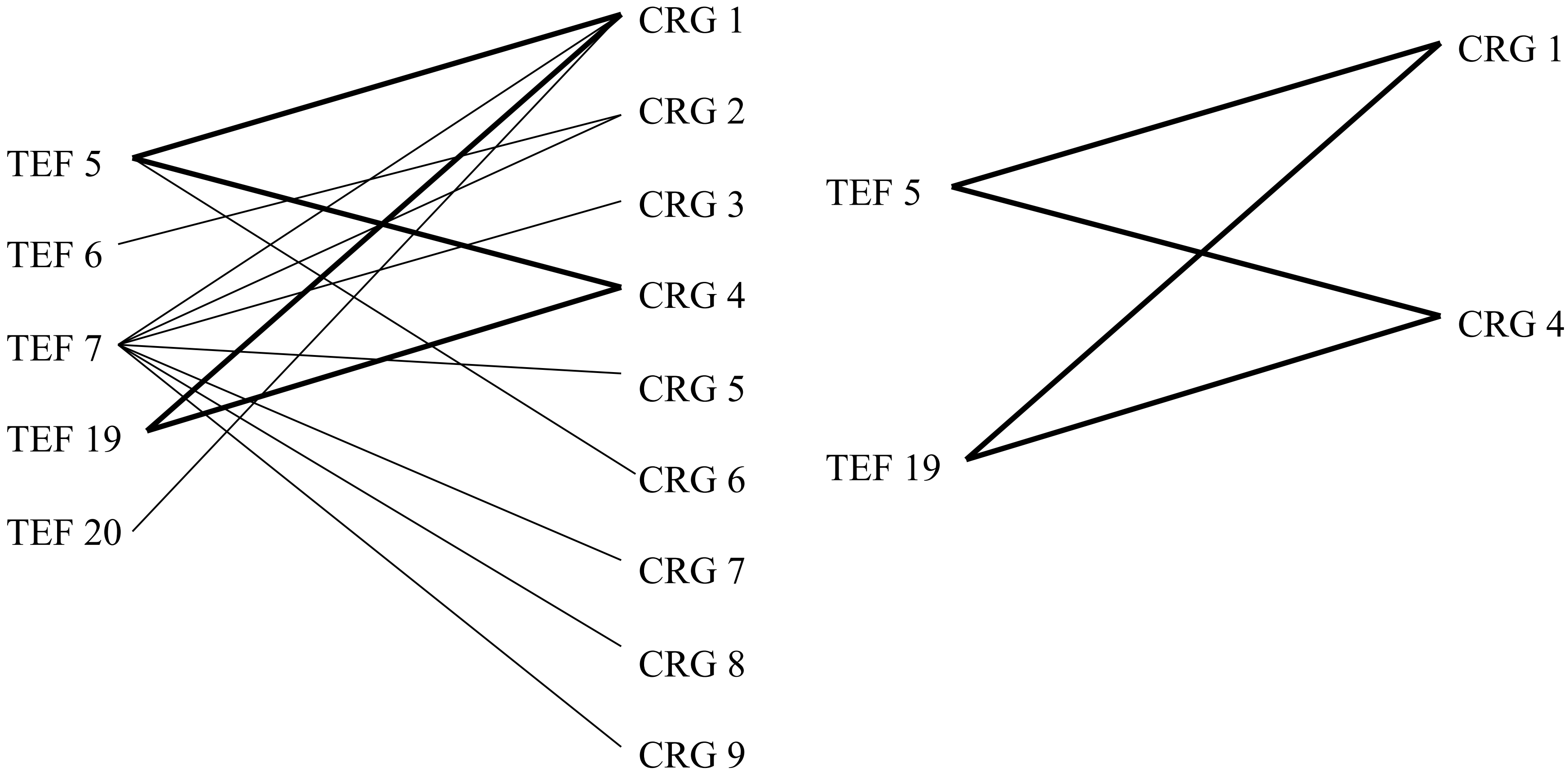
CRG 1
CRG 2
CRG 3
CRG 4
CRG 5
CRG 6
CRG 7
CRG 8
CRG 9
TEF 5
TEF 6
TEF 7
TEF 19
TEF 20
CRG 1
CRG 4
TEF 5
TEF 19

*TEF1 v. FHB1*

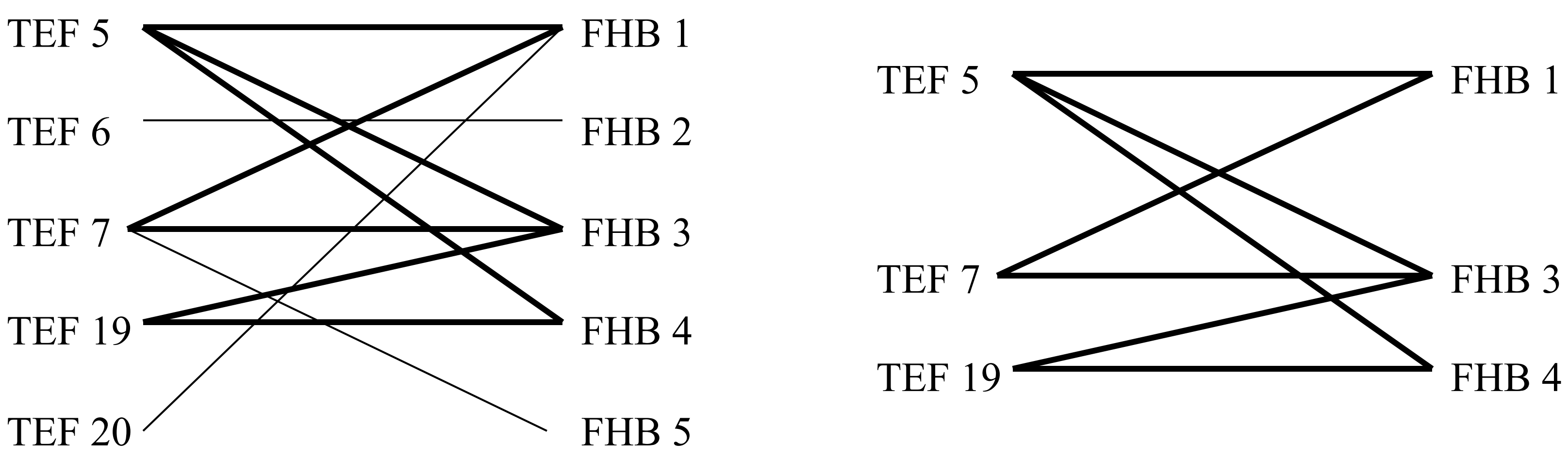
TEF 5
TEF 6
TEF 7
TEF 19
TEF 20
FHB 1
FHB 2
FHB 3
FHB 4
FHB 5
TEF 5
TEF 7
TEF 19
FHB 1
FHB 3
FHB 4

*TEF1 v. FTR1*

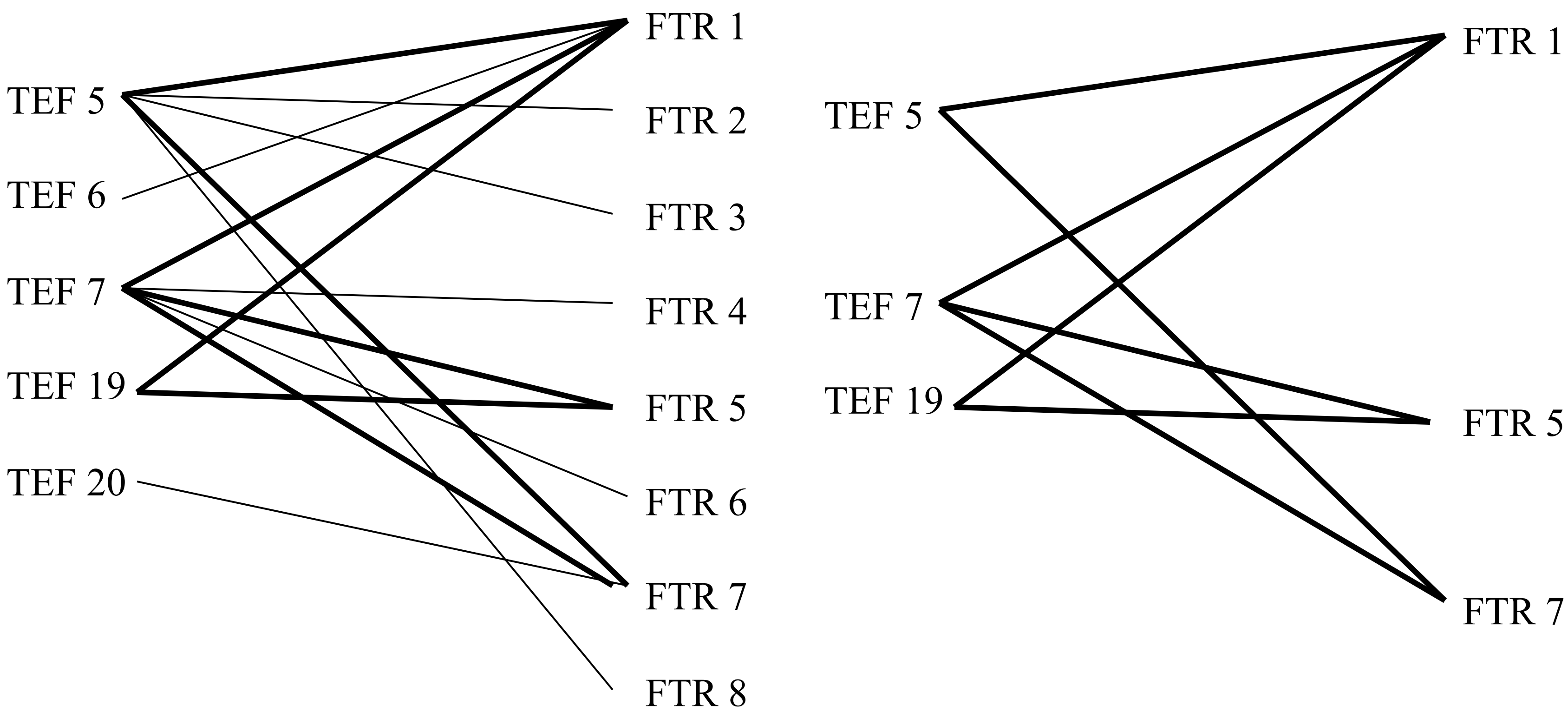
TEF 5
TEF 6
TEF 7
TEF 19
TEF 20
FTR 1
FTR 2
FTR 3
FTR 4
FTR 5
FTR 6
FTR 7
FTR 8
TEF 5
TEF 7
TEF 19
FTR 1
FTR 5
FTR 7

# *TEF1 v. CAP59*

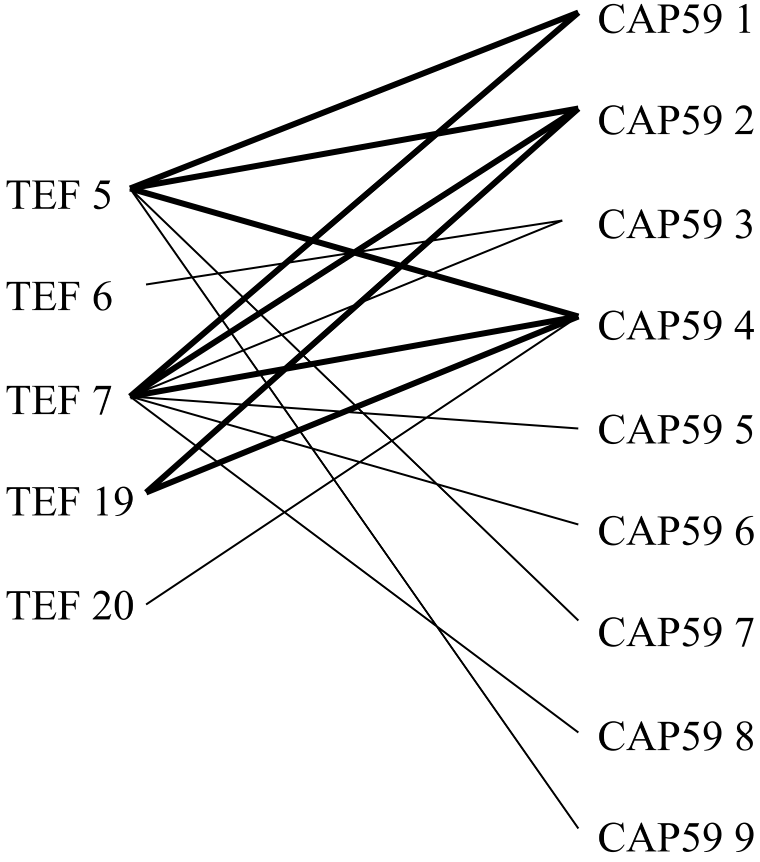
TEF 5
TEF 6
TEF 7
TEF 19
TEF 20
CAP59 1
CAP59 2
CAP59 3
CAP59 4
CAP59 5
CAP59 6
CAP59 7
CAP59 8
CAP59 9

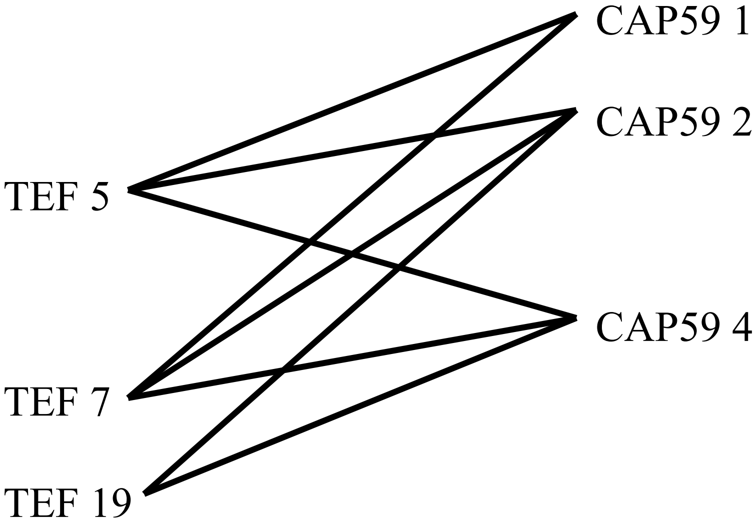
TEF 5
TEF 7
TEF 19
CAP59 1
CAP59 2
CAP59 4

# *GPD1 v. LAC1*

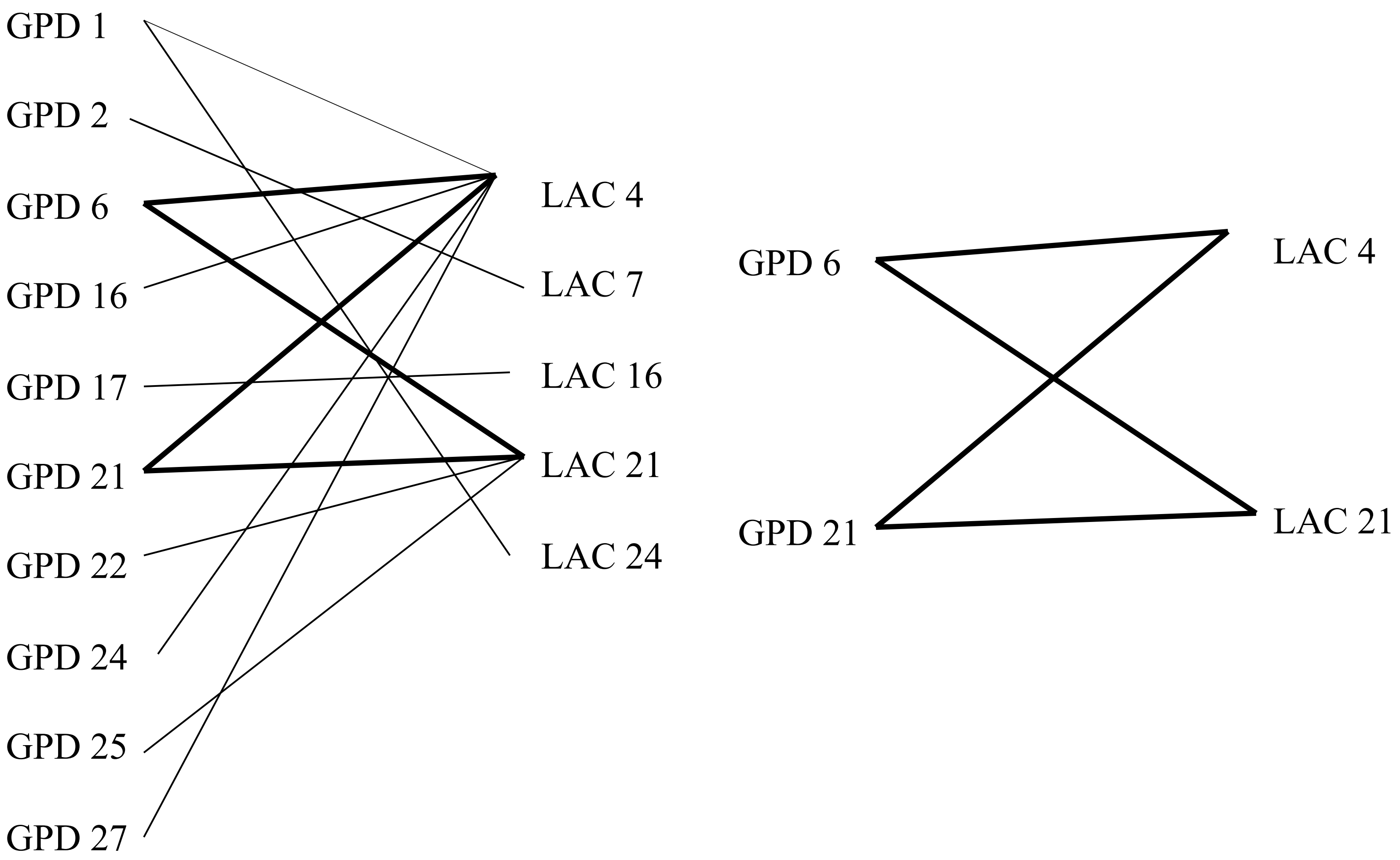
GPD 1
GPD 2
GPD 6
GPD 16
GPD 17
GPD 21
GPD 22
GPD 24
GPD 25
GPD 27
LAC 4
LAC 7
LAC 16
LAC 21
LAC 24
GPD 6
GPD 21
LAC 4
LAC 21

# *GPD1 v. CAP10*

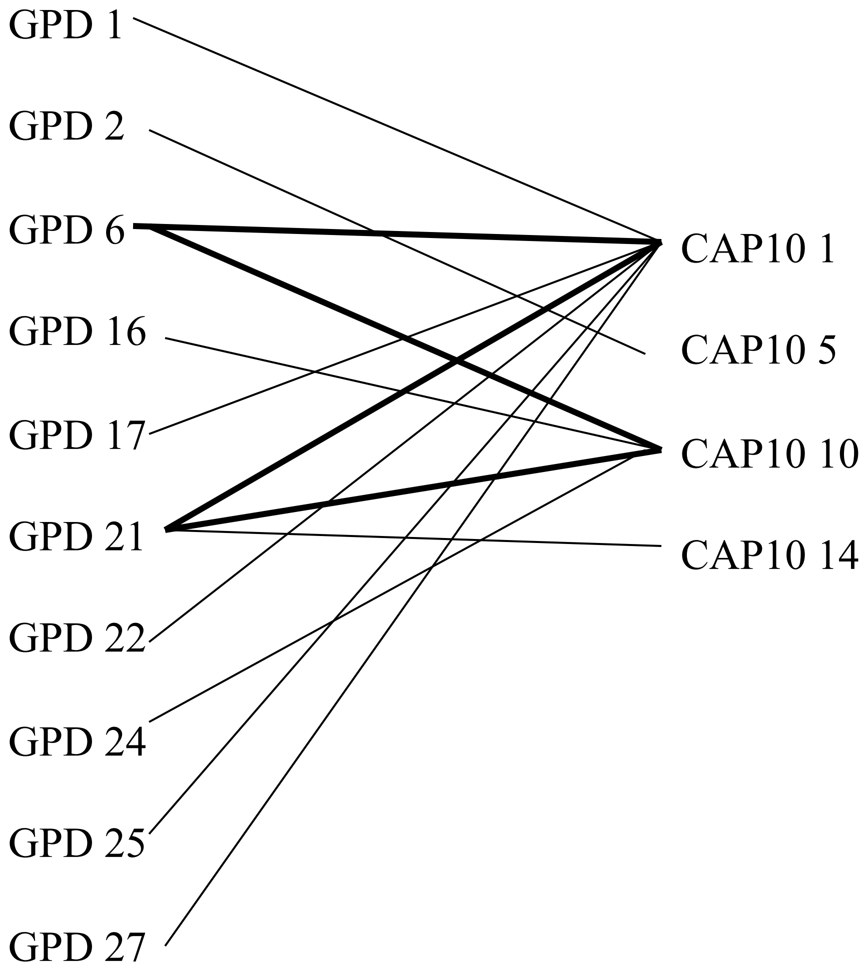
GPD 1
GPD 2
GPD 6
GPD 16
GPD 17
GPD 21
GPD 22
GPD 24
GPD 25
GPD 27
CAP10 1
CAP10 5
CAP10 10
CAP10 14

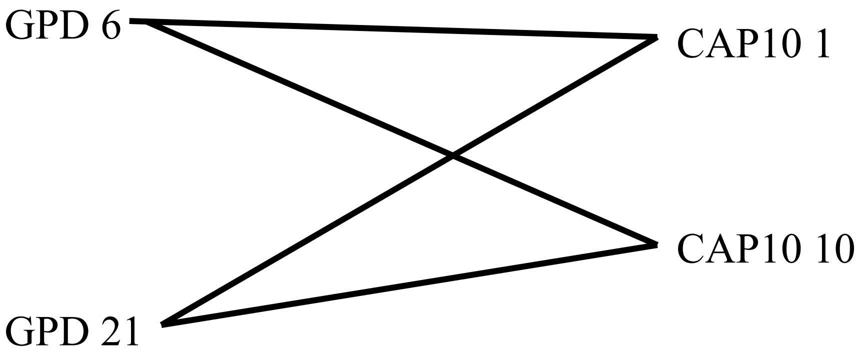
GPD 6
GPD 21
CAP10 1
CAP10 10

# *GPD1 v. PLB1*

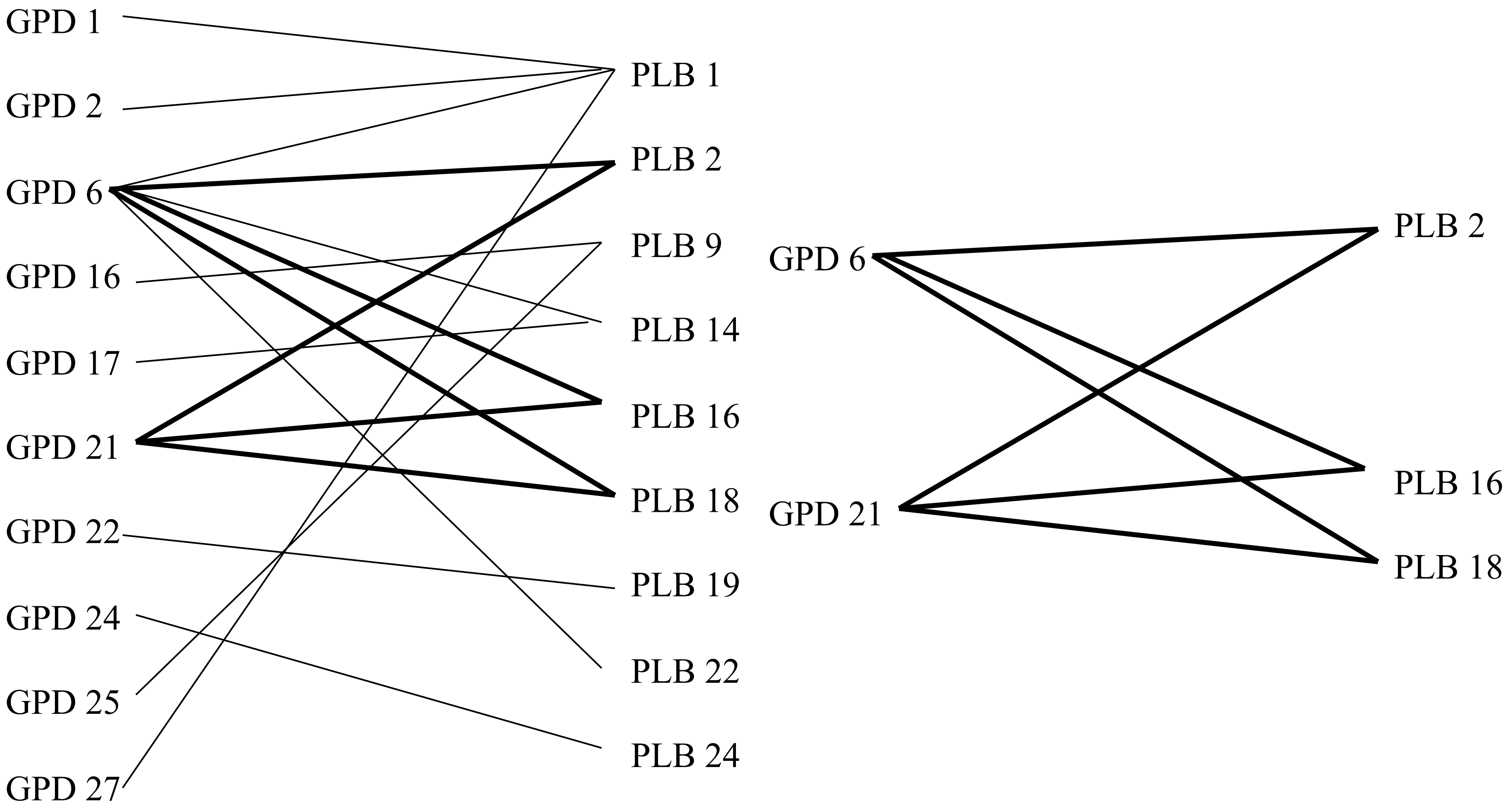
GPD 1
GPD 2
GPD 6
GPD 16
GPD 17
GPD 21
GPD 22
GPD 24
GPD 25
GPD 27
PLB 1
PLB 2
PLB 9
PLB 14
PLB 16
PLB 18
PLB 19
PLB 22
PLB 24
GPD 6
GPD 21
PLB 2
PLB 16
PLB 18

# *GPD1 v. TOR1*

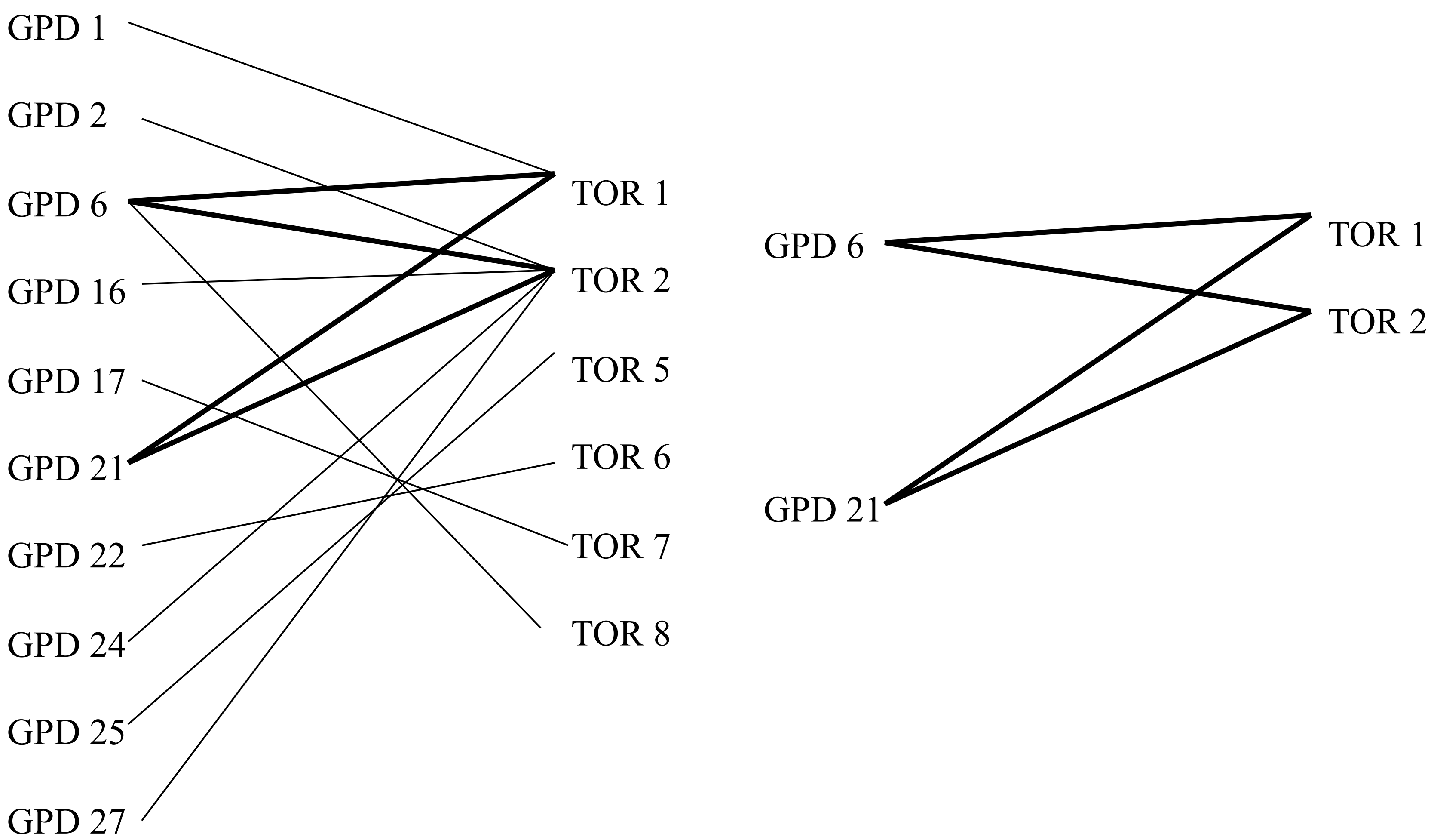

GPD 1
GPD 2
GPD 6
GPD 16
GPD 17
GPD 21
GPD 22
GPD 24
GPD 25
GPD 27
TOR 1
TOR 2
TOR 5
TOR 6
TOR 7
TOR 8
GPD 6
GPD 21
TOR 1
TOR 2

# *GPD1 v. CRG1*

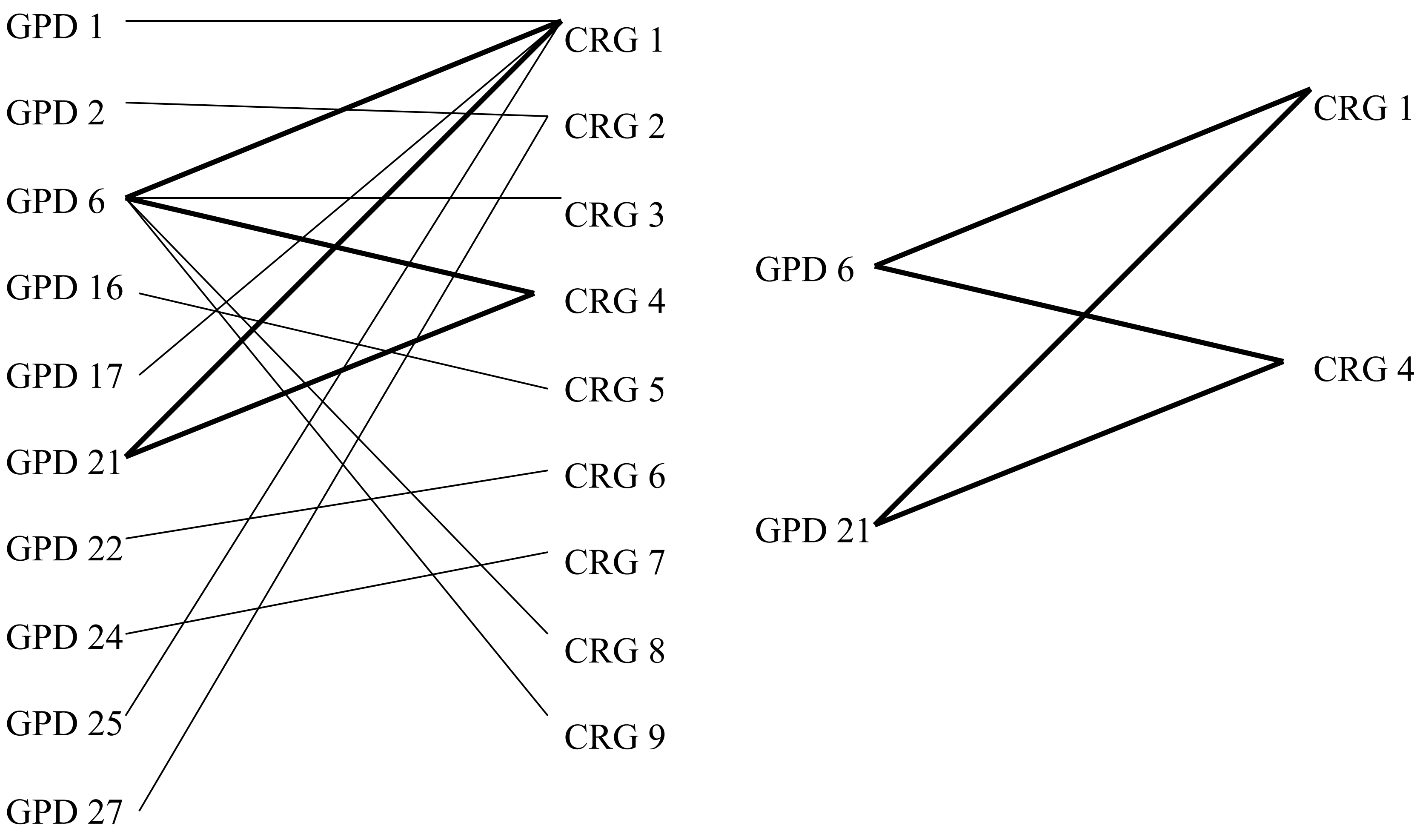
GPD 1
GPD 2
GPD 6
GPD 16
GPD 17
GPD 21
GPD 22
GPD 24
GPD 25
GPD 27
CRG 1
CRG 2
CRG 3
CRG 4
CRG 5
CRG 6
CRG 7
CRG 8
CRG 9
GPD 6
GPD 21
CRG 1
CRG 4

# *GPD1 v. FHB1*

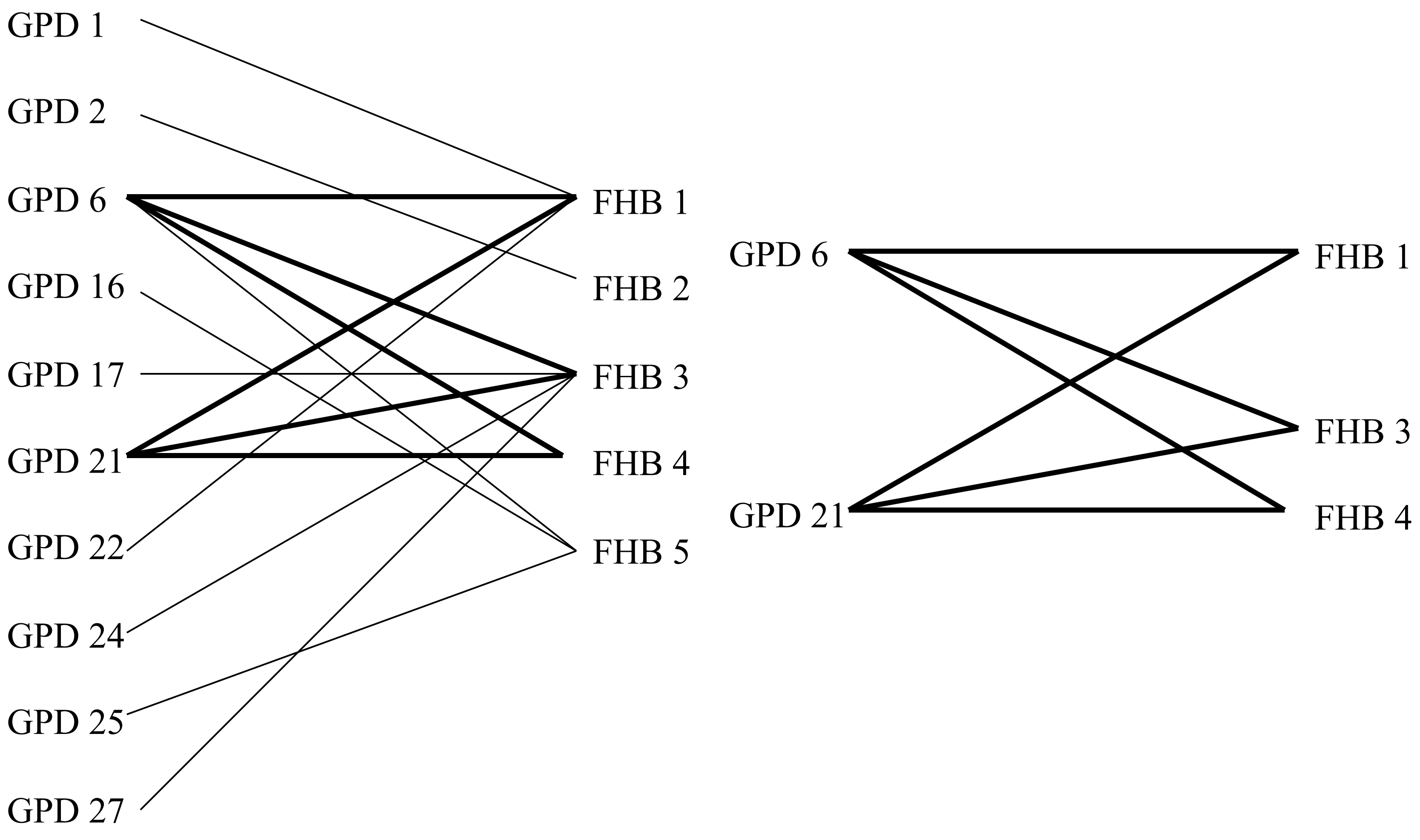
GPD 1
GPD 2
GPD 6
GPD 16
GPD 17
GPD 21
GPD 22
GPD 24
GPD 25
GPD 27
FHB 1
FHB 2
FHB 3
FHB 4
FHB 5
GPD 6
GPD 21
FHB 1
FHB 3
FHB 4

# *GPD1 v. FTR1*

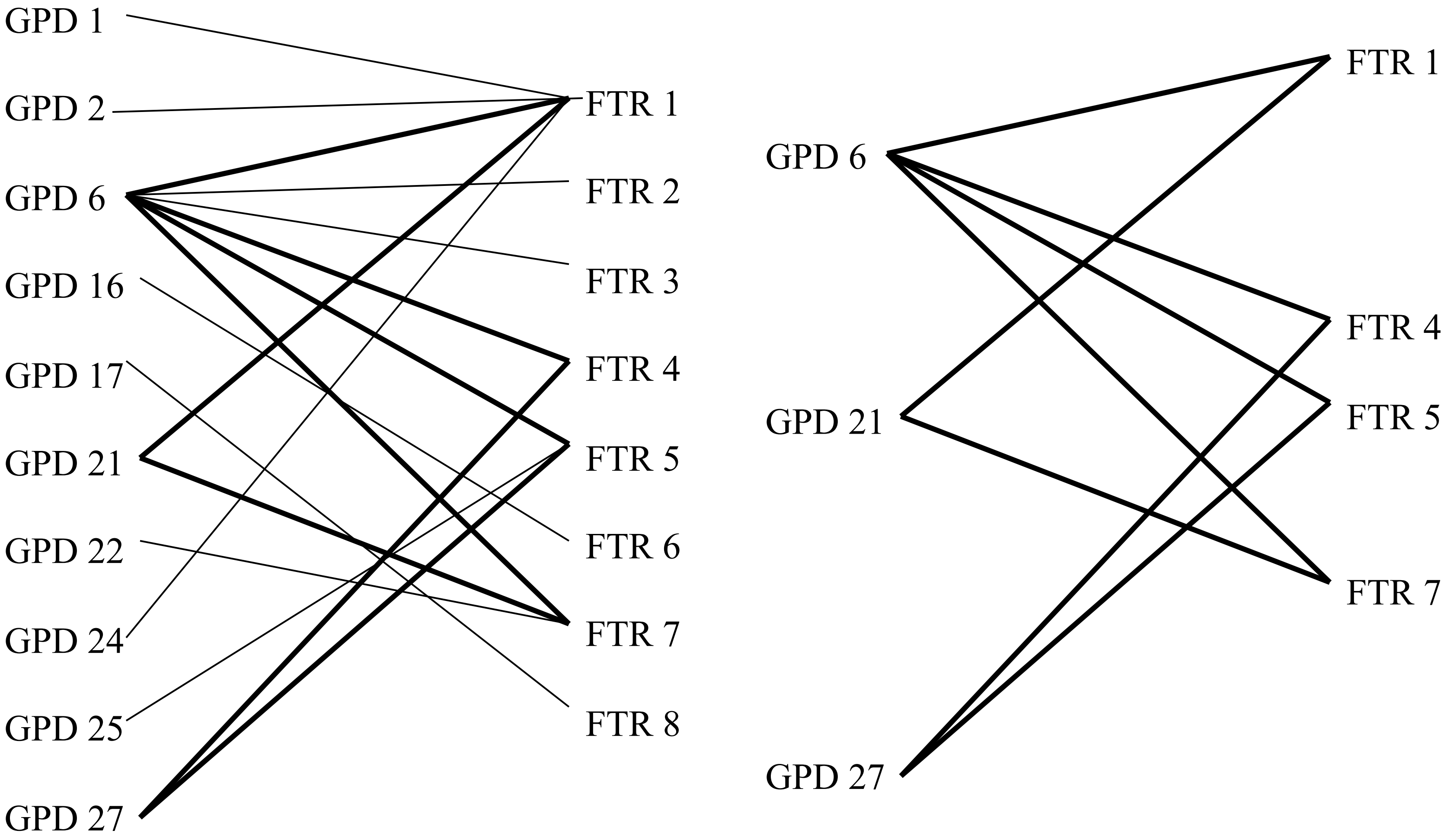
GPD 1
GPD 2
GPD 6
GPD 16
GPD 17
GPD 21
GPD 22
GPD 24
GPD 25
GPD 27
FTR 1
FTR 2
FTR 3
FTR 4
FTR 5
FTR 6
FTR 7
FTR 8
GPD 6
GPD 21
GPD 27
FTR 1
FTR 4
FTR 5
FTR 7

*LAC1 v. PLB1*

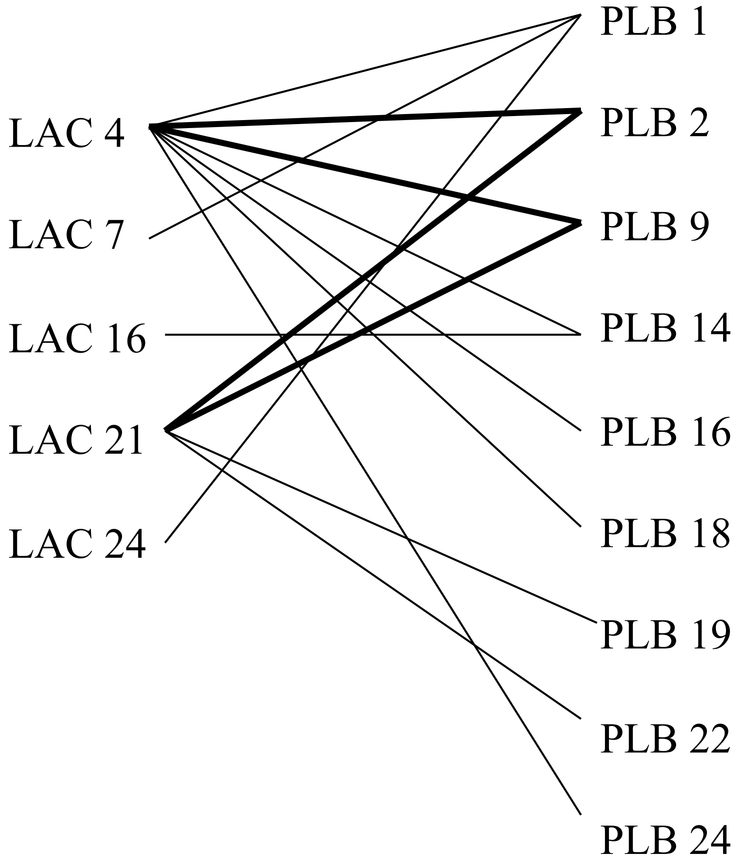
PLB 1
LAC 4
PLB 2
LAC 7
PLB 9
LAC 16
PLB 14
LAC 21
PLB 16
LAC 24
PLB 18
PLB 19
PLB 22
PLB 24

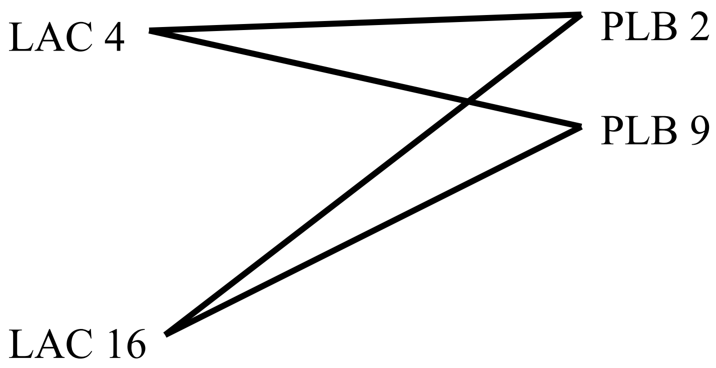
LAC 4
PLB 2
PLB 9
LAC 16

# *LAC1 v. HOG1*

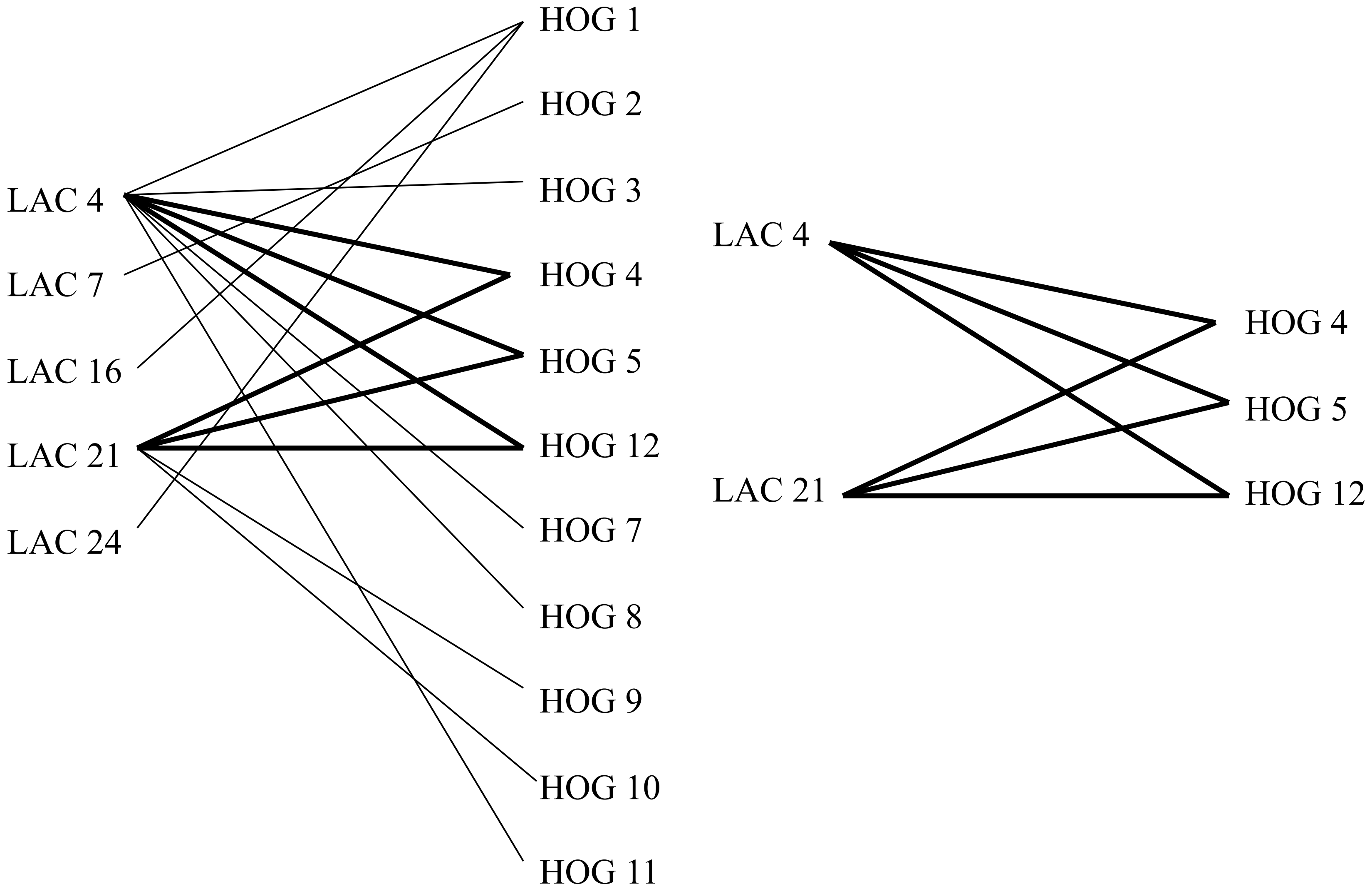
LAC 4
LAC 7
LAC 16
LAC 21
LAC 24
HOG 1
HOG 2
HOG 3
HOG 4
HOG 5
HOG 12
HOG 7
HOG 8
HOG 9
HOG 10
HOG 11
LAC 4
LAC 21
HOG 4
HOG 5
HOG 12

*LAC1 v. TOR1*

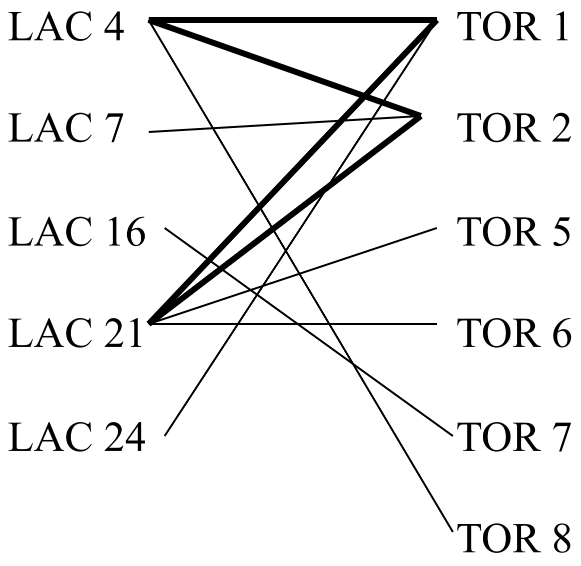
LAC 4
LAC 7
LAC 16
LAC 21
LAC 24
TOR 1
TOR 2
TOR 5
TOR 6
TOR 7
TOR 8

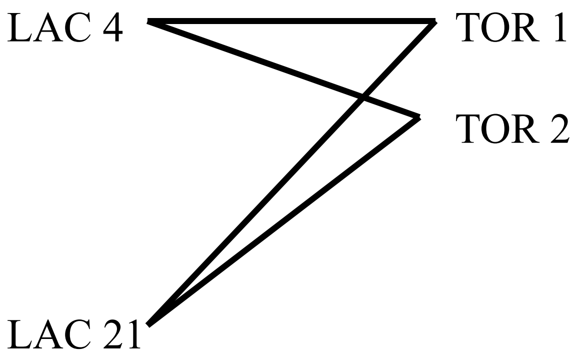
LAC 4
LAC 21
TOR 1
TOR 2

*LAC1 v. CRG1*

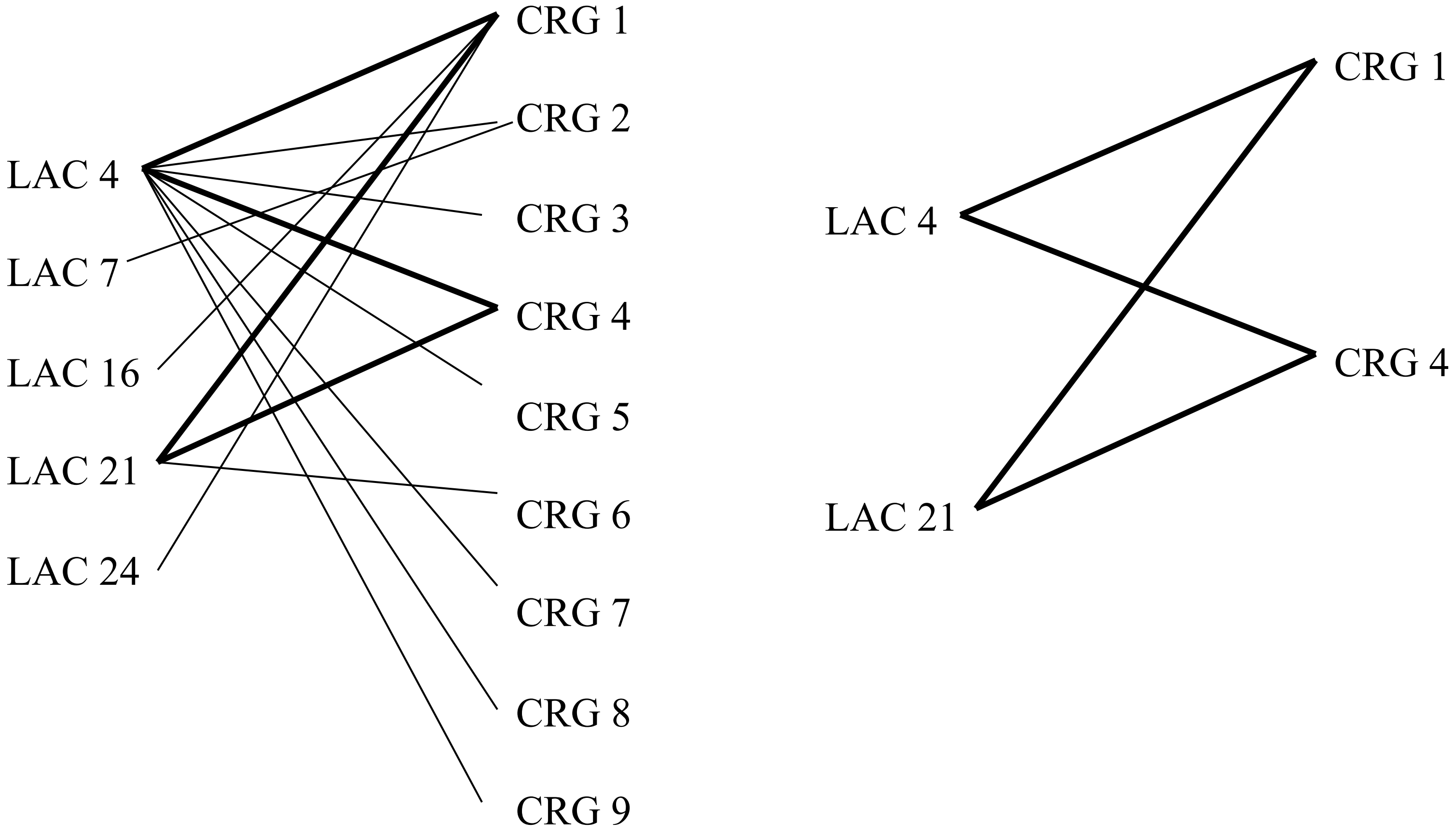
LAC 4
LAC 7
LAC 16
LAC 21
LAC 24
CRG 1
CRG 2
CRG 3
CRG 4
CRG 5
CRG 6
CRG 7
CRG 8
CRG 9
LAC 4
LAC 21
CRG 1
CRG 4

# *LAC1 v. FHB1*

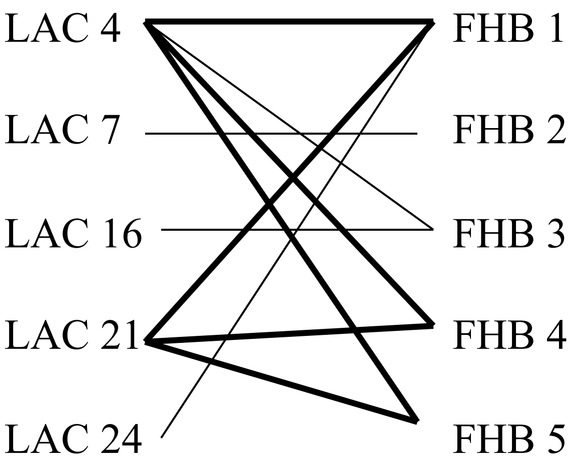
LAC 4
LAC 7
LAC 16
LAC 21
LAC 24
FHB 1
FHB 2
FHB 3
FHB 4
FHB 5

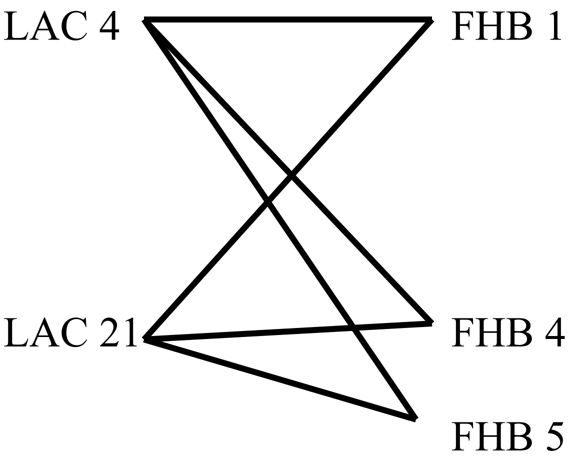
LAC 4
LAC 21
FHB 1
FHB 4
FHB 5

# *LAC1 v. FTR1*

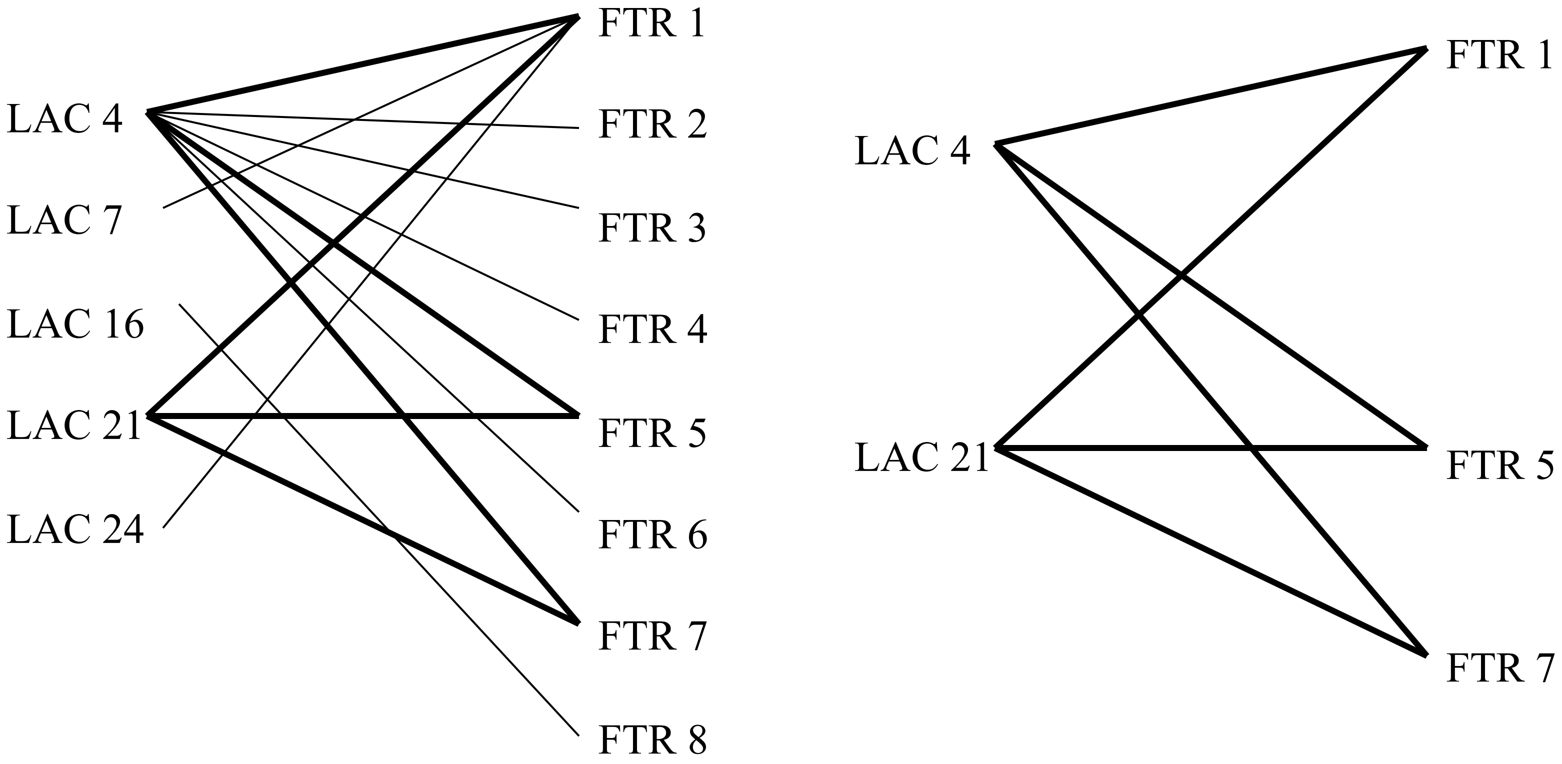
FTR 1
LAC 4
FTR 2
LAC 7
FTR 3
LAC 16
FTR 4
LAC 21
FTR 5
LAC 24
FTR 6
FTR 7
FTR 8
FTR 1
LAC 4
LAC 21
FTR 5
FTR 7

*LAC1 v. CAP59*

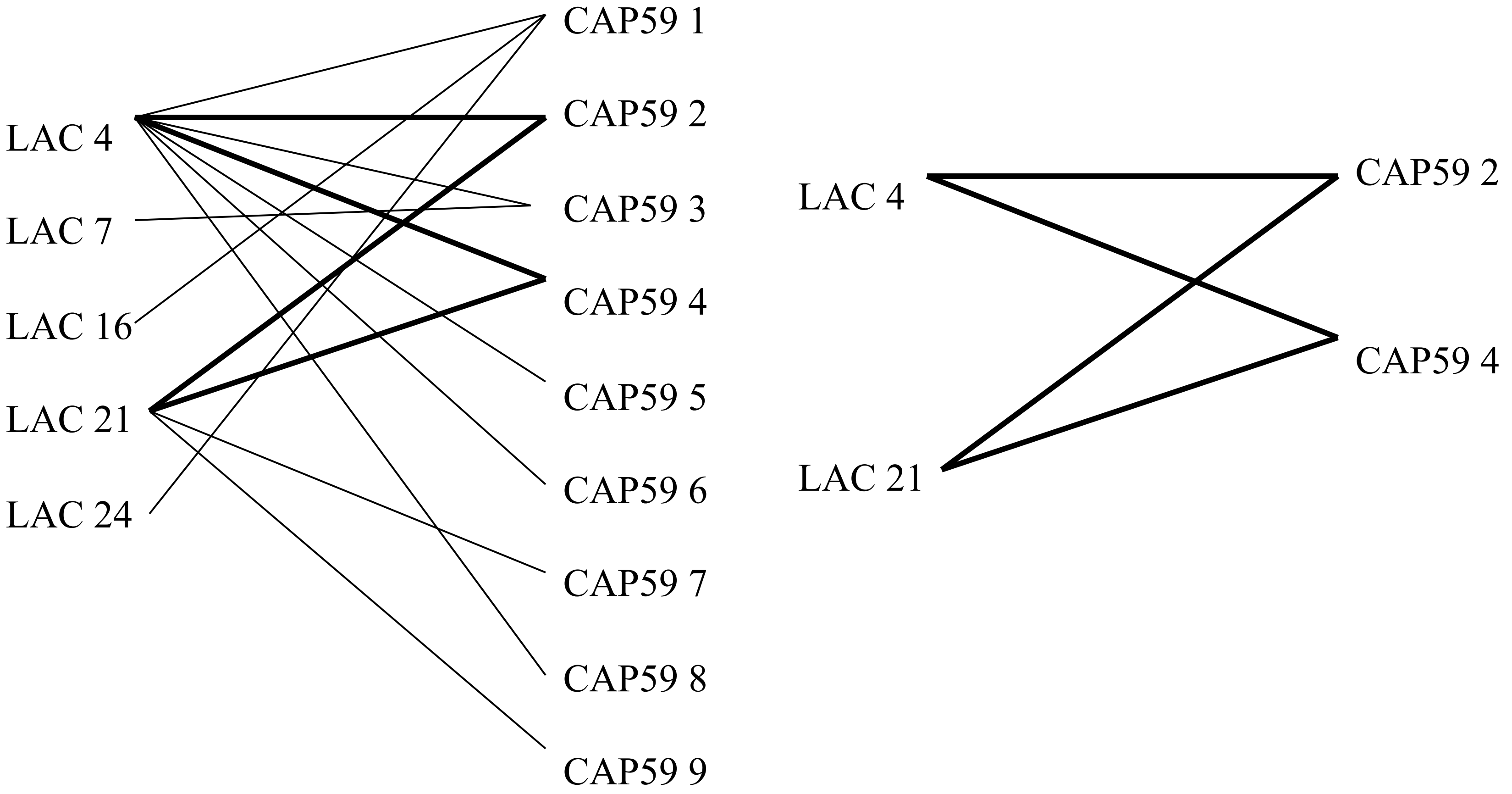

LAC 4
LAC 7
LAC 16
LAC 21
LAC 24
CAP59 1
CAP59 2
CAP59 3
CAP59 4
CAP59 5
CAP59 6
CAP59 7
CAP59 8
CAP59 9
LAC 4
LAC 21
CAP59 2
CAP59 4

## *CAP10 v. PLB1*

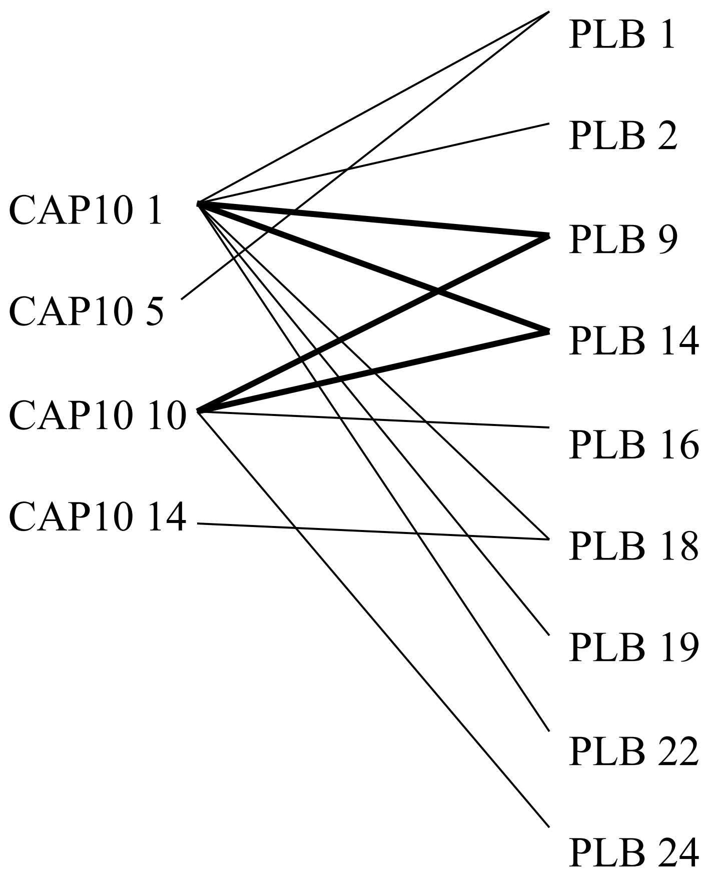

CAP10 1
CAP10 5
CAP10 10
CAP10 14
PLB 1
PLB 2
PLB 9
PLB 14
PLB 16
PLB 18
PLB 19
PLB 22
PLB 24


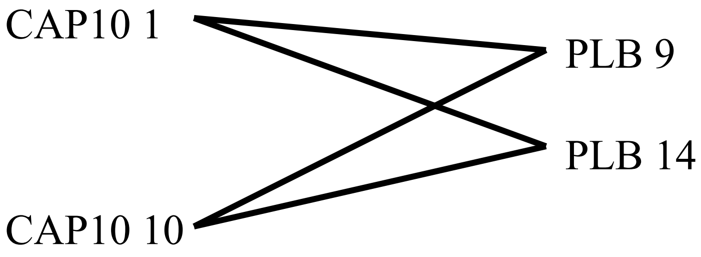

CAP10 1
CAP10 10
PLB 9
PLB 14

# *CAP10 v. HOG1*

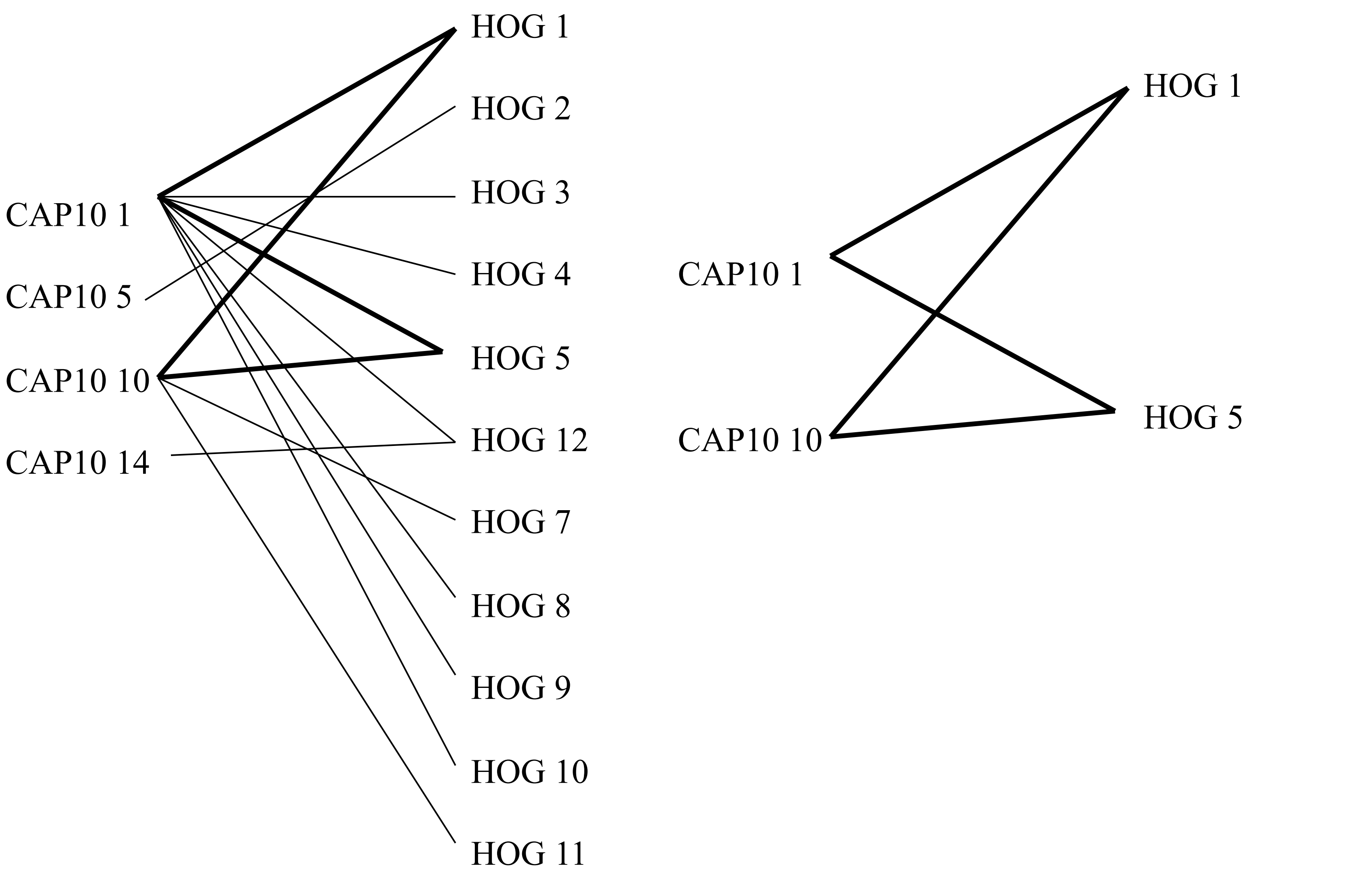

HOG 1
HOG 2
HOG 3
HOG 4
HOG 5
HOG 12
HOG 7
HOG 8
HOG 9
HOG 10
HOG 11
CAP10 1
CAP10 5
CAP10 10
CAP10 14
HOG 1
HOG 5
CAP10 1
CAP10 10

# *CAP10 v. CNB1*

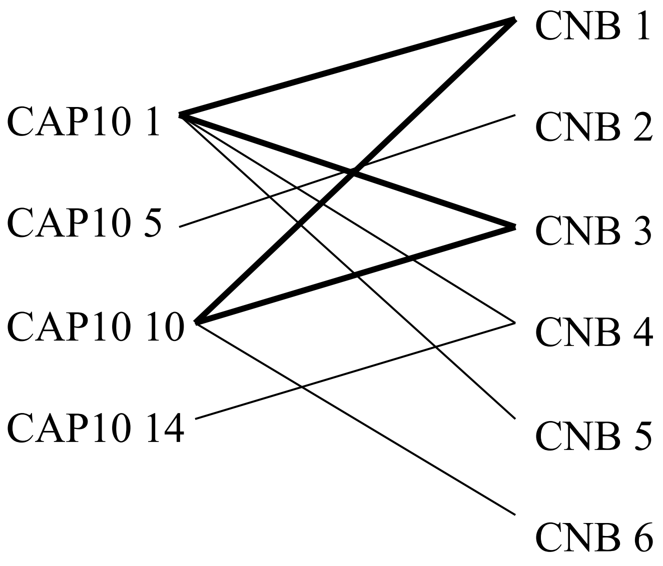
CAP10 1
CAP10 5
CAP10 10
CAP10 14
CNB 1
CNB 2
CNB 3
CNB 4
CNB 5
CNB 6

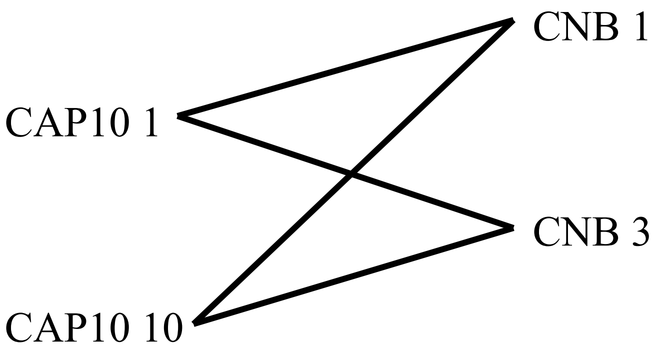
CAP10 1
CAP10 10
CNB 1
CNB 3

*PLB1 v. CNB1*

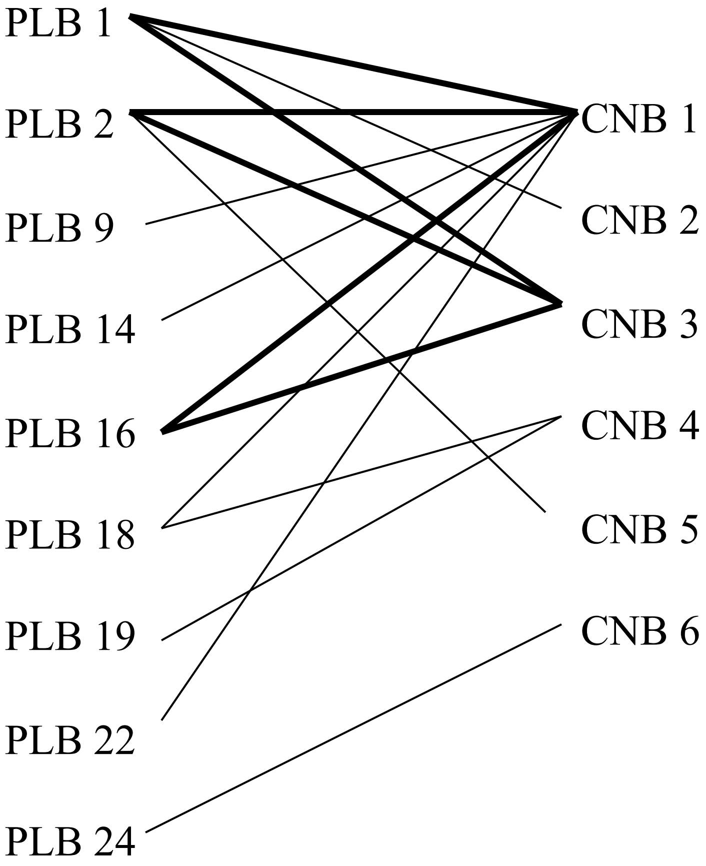
PLB 1
PLB 2
PLB 9
PLB 14
PLB 16
PLB 18
PLB 19
PLB 22
PLB 24
CNB 1
CNB 2
CNB 3
CNB 4
CNB 5
CNB 6

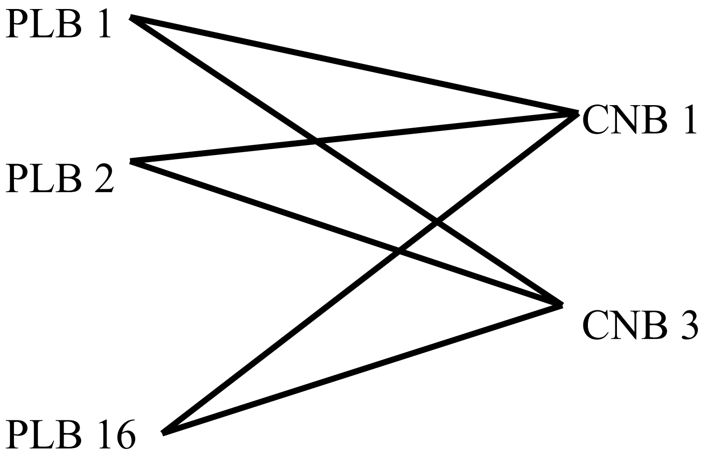
PLB 1
PLB 2
PLB 16
CNB 1
CNB 3

*PLB1 v. TOR1*

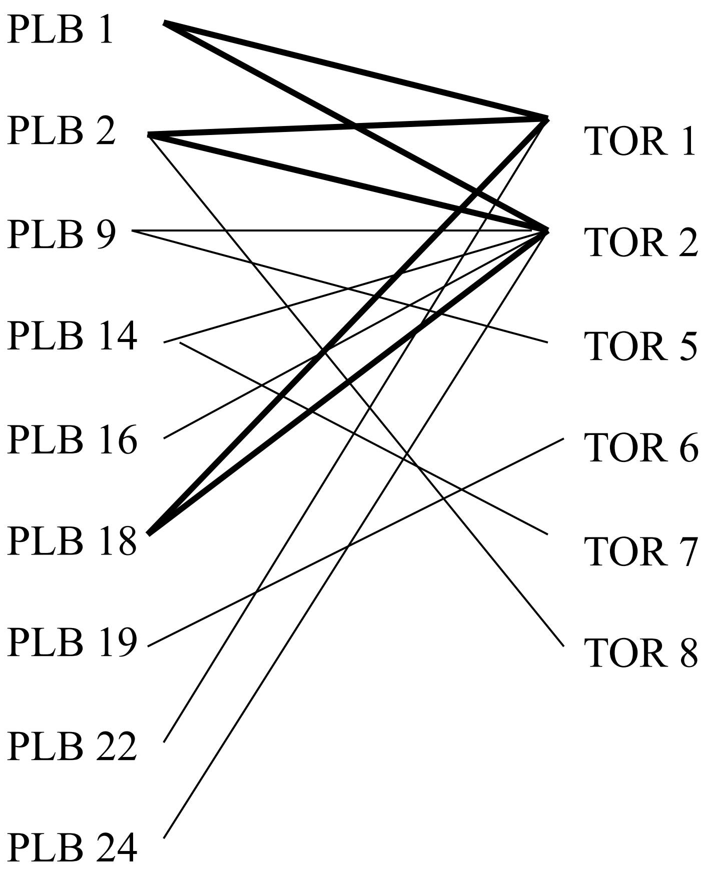
PLB 1
PLB 2
PLB 9
PLB 14
PLB 16
PLB 18
PLB 19
PLB 22
PLB 24
TOR 1
TOR 2
TOR 5
TOR 6
TOR 7
TOR 8

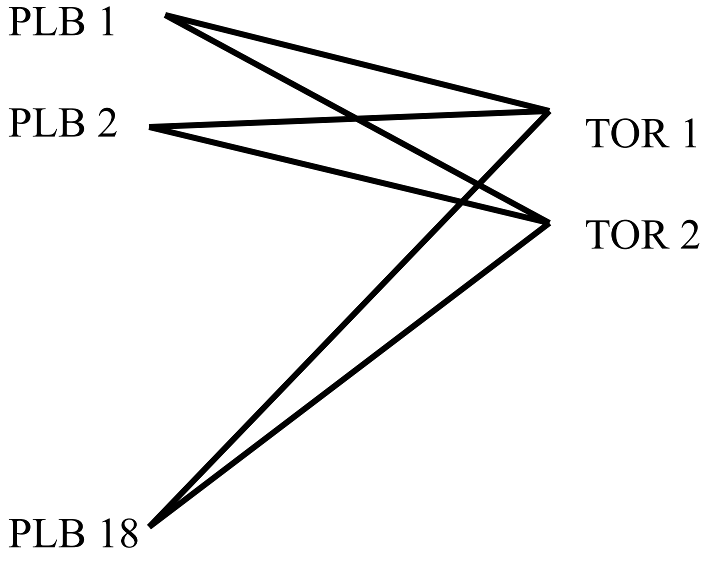
PLB 1
PLB 2
PLB 18
TOR 1
TOR 2

# *PLB1 v. FHB1*

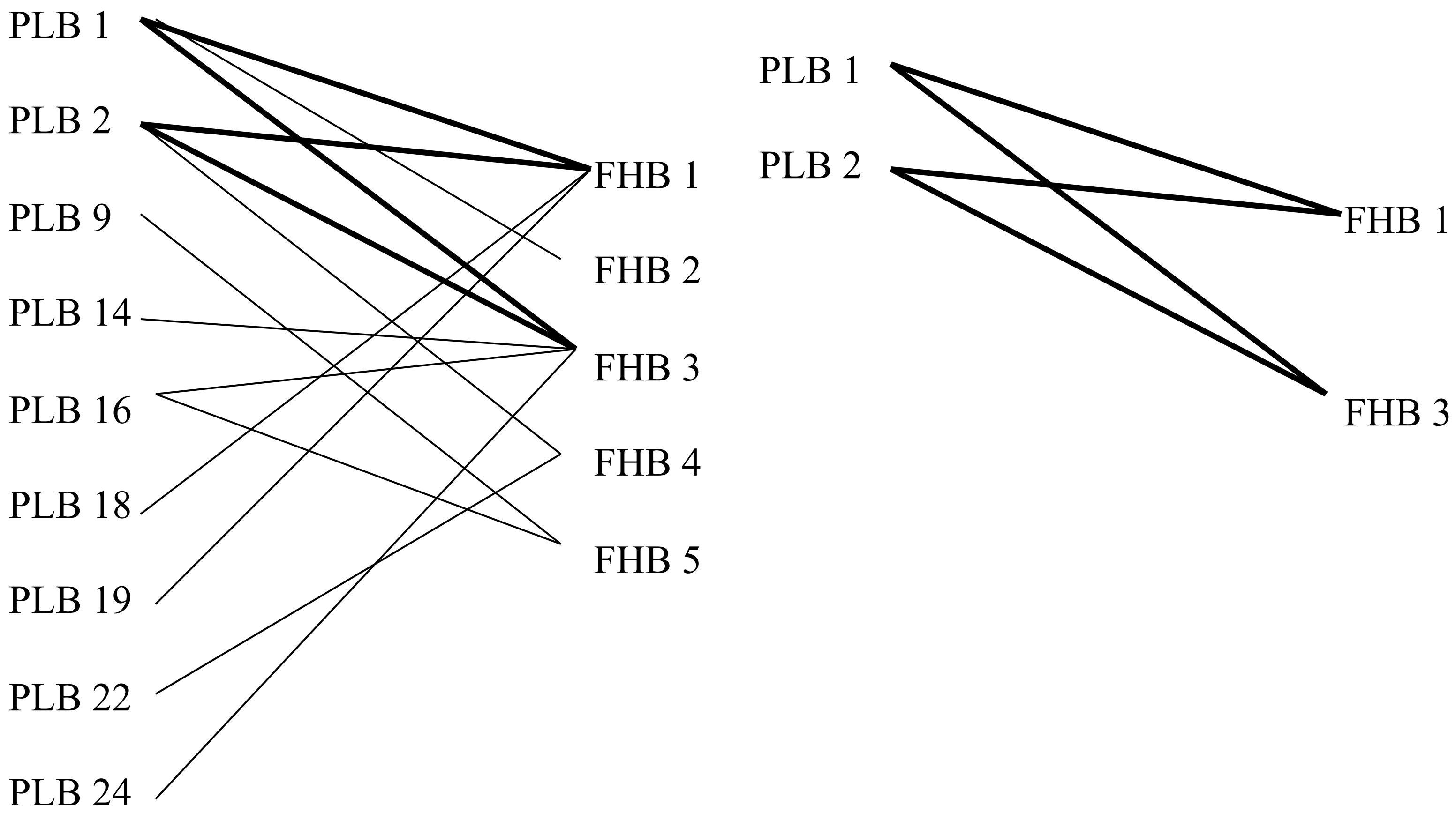
PLB 1
PLB 2
PLB 9
PLB 14
PLB 16
PLB 18
PLB 19
PLB 22
PLB 24
FHB 1
FHB 2
FHB 3
FHB 4
FHB 5
PLB 1
PLB 2
FHB 1
FHB 3

*PLB1 v. FTR1*

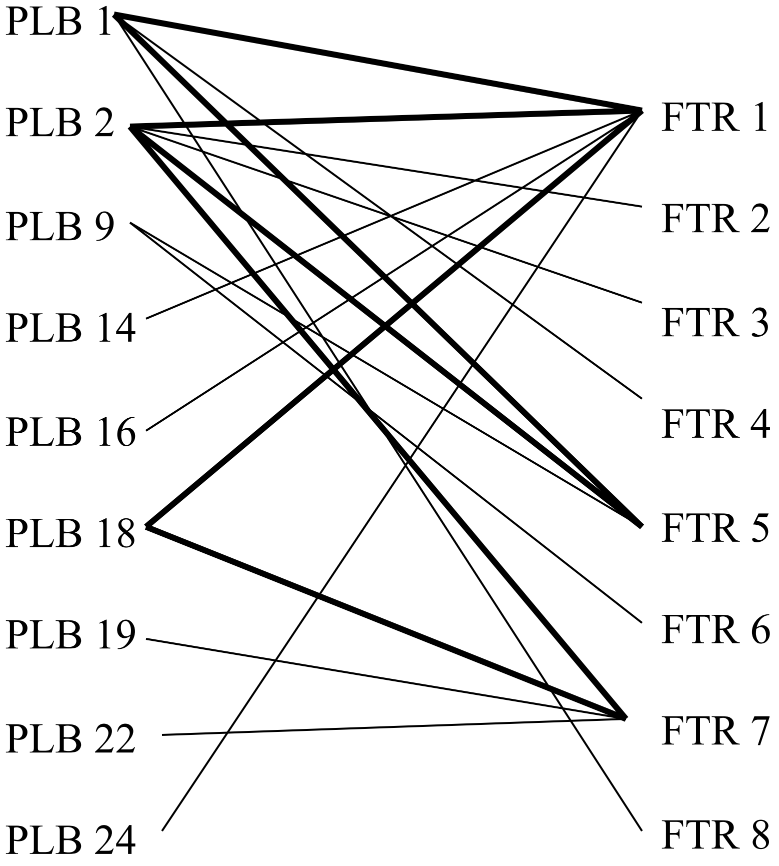
PLB 1
PLB 2
PLB 9
PLB 14
PLB 16
PLB 18
PLB 19
PLB 22
PLB 24
FTR 1
FTR 2
FTR 3
FTR 4
FTR 5
FTR 6
FTR 7
FTR 8

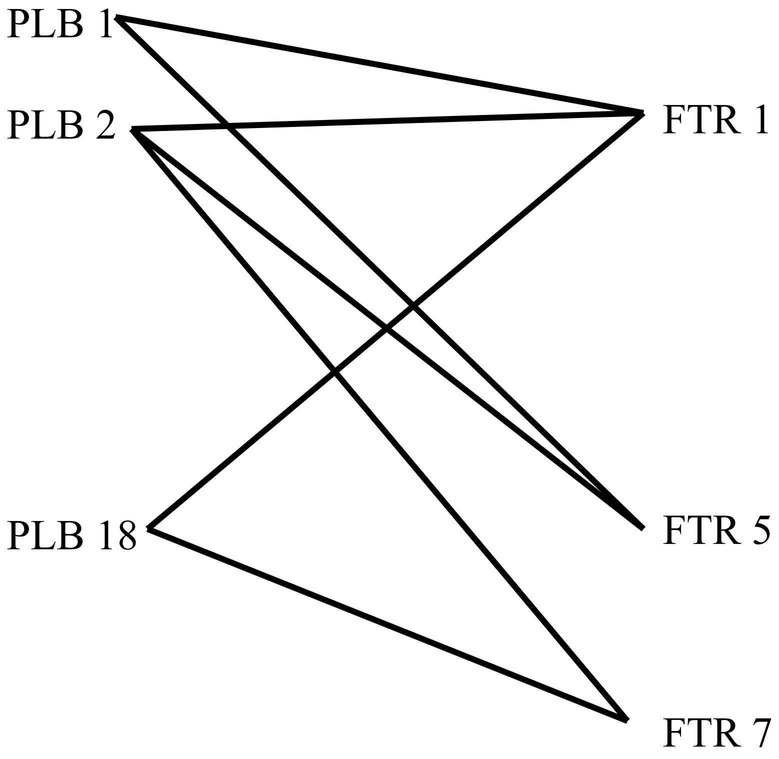
PLB 1
PLB 2
PLB 18
FTR 1
FTR 5
FTR 7

# *PLB1 v. CAP59*

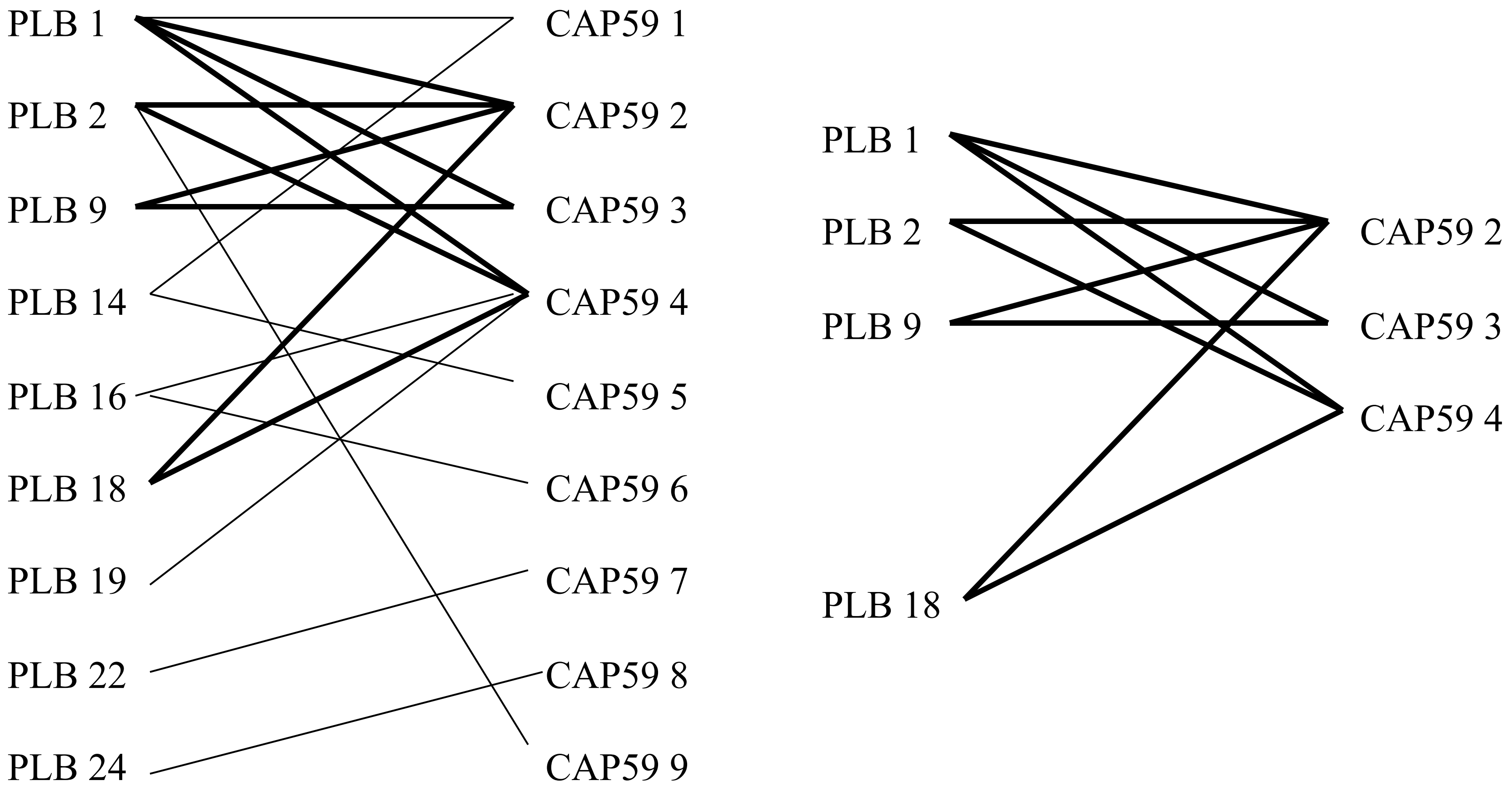
PLB 1
PLB 2
PLB 9
PLB 14
PLB 16
PLB 18
PLB 19
PLB 22
PLB 24
CAP59 1
CAP59 2
CAP59 3
CAP59 4
CAP59 5
CAP59 6
CAP59 7
CAP59 8
CAP59 9
PLB 1
PLB 2
PLB 9
PLB 18
CAP59 2
CAP59 3
CAP59 4

# *HOG1 v. TOR1*

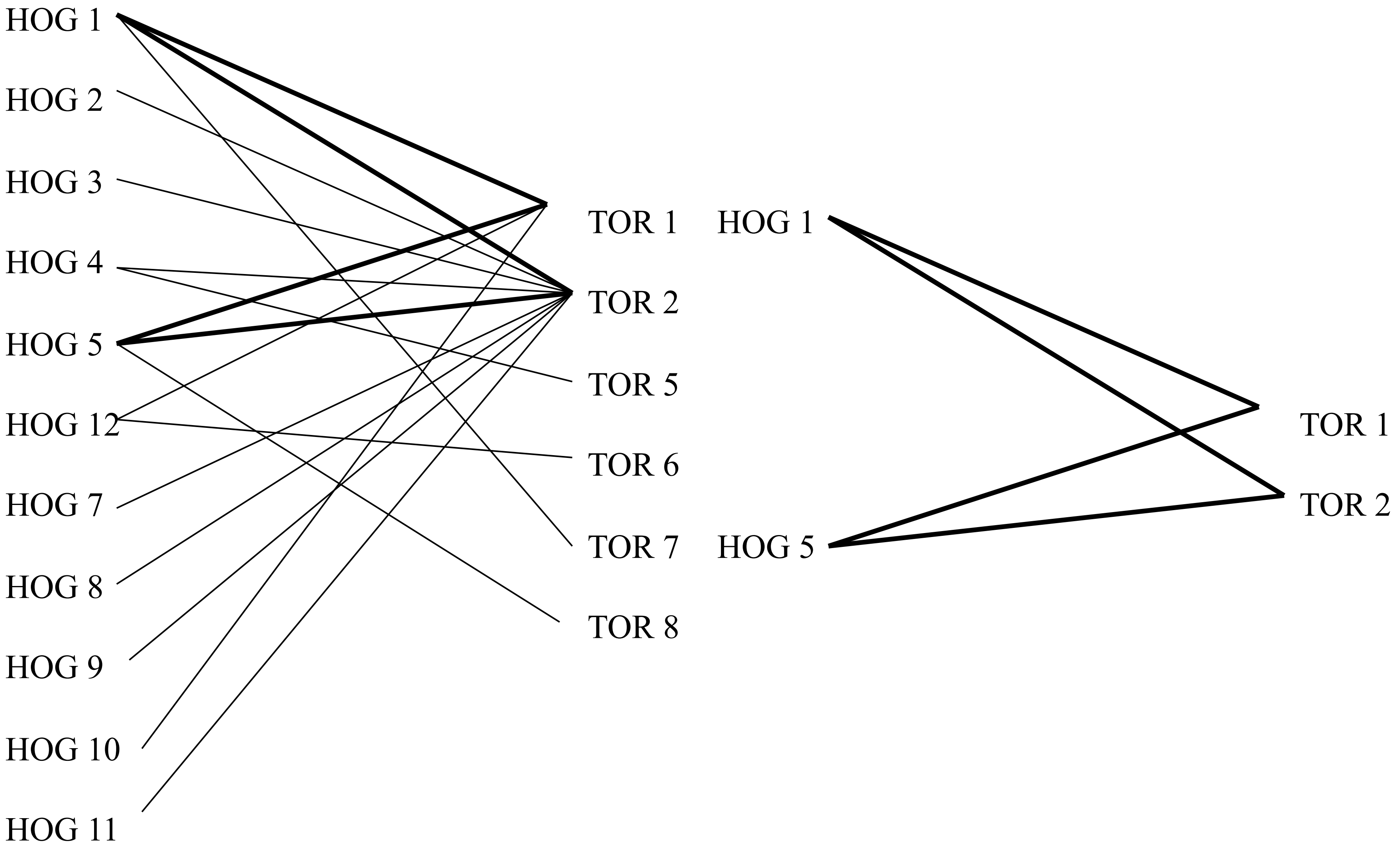
HOG 1
HOG 2
HOG 3
HOG 4
HOG 5
HOG 12
HOG 7
HOG 8
HOG 9
HOG 10
HOG 11
TOR 1
TOR 2
TOR 5
TOR 6
TOR 7
TOR 8
HOG 1
HOG 5
TOR 1
TOR 2

# *HOG1 v. FHB1*

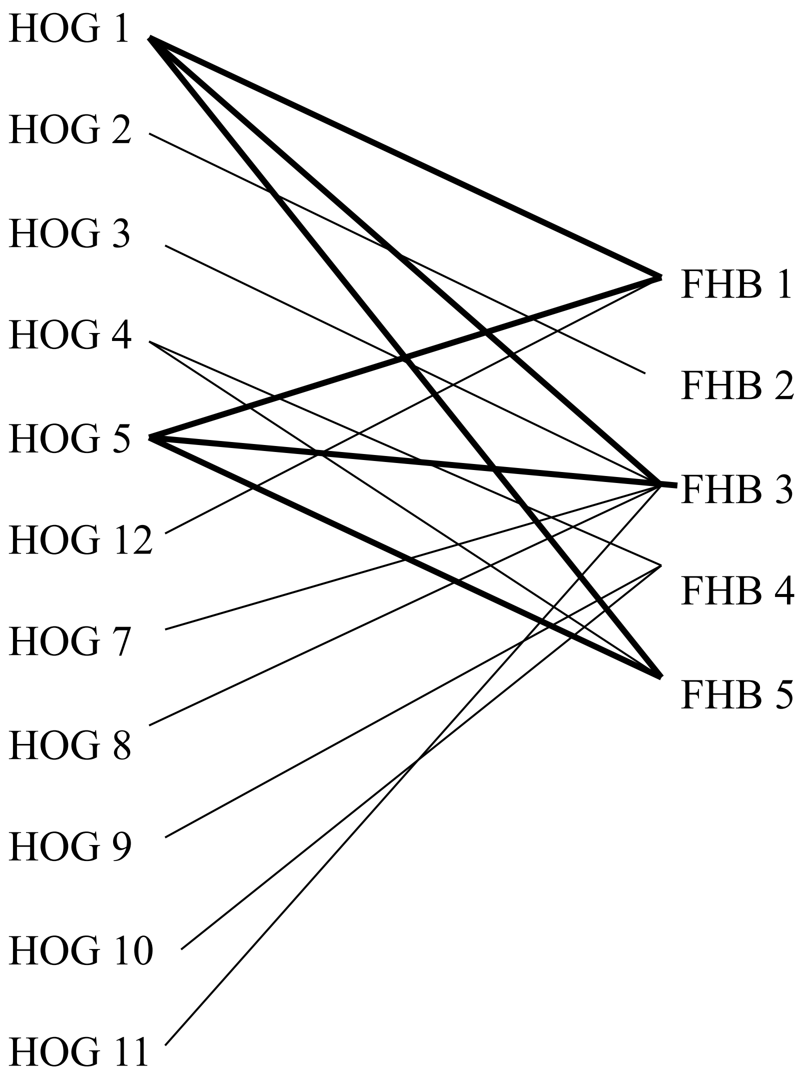
HOG 1
HOG 2
HOG 3
HOG 4
HOG 5
HOG 12
HOG 7
HOG 8
HOG 9
HOG 10
HOG 11
FHB 1
FHB 2
FHB 3
FHB 4
FHB 5

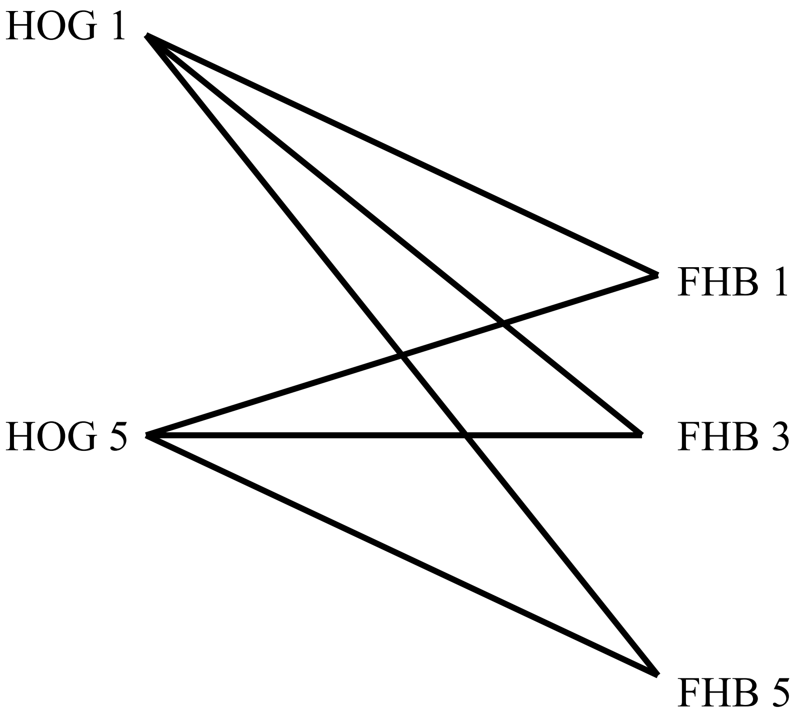
HOG 1
HOG 5
FHB 1
FHB 3
FHB 5

# *CNB1 v. FTR1*

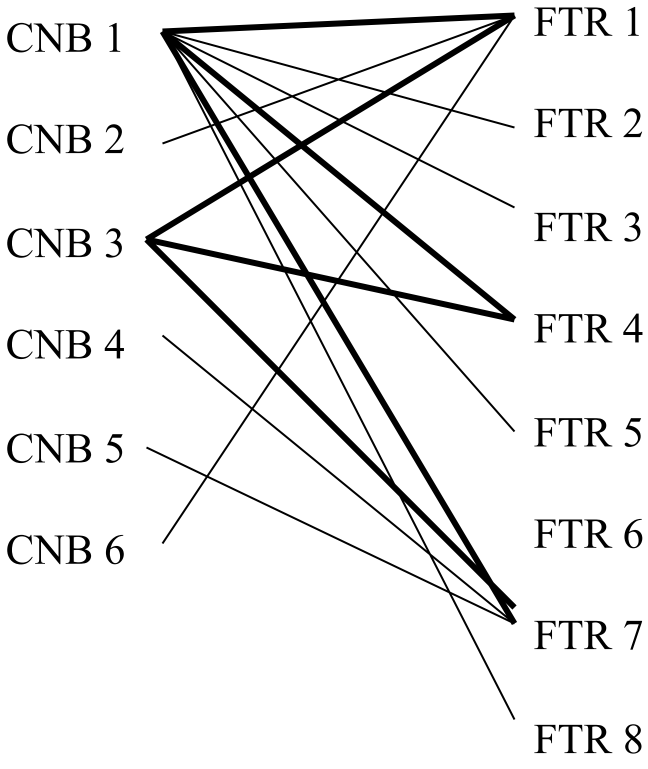
CNB 1
CNB 2
CNB 3
CNB 4
CNB 5
CNB 6
FTR 1
FTR 2
FTR 3
FTR 4
FTR 5
FTR 6
FTR 7
FTR 8

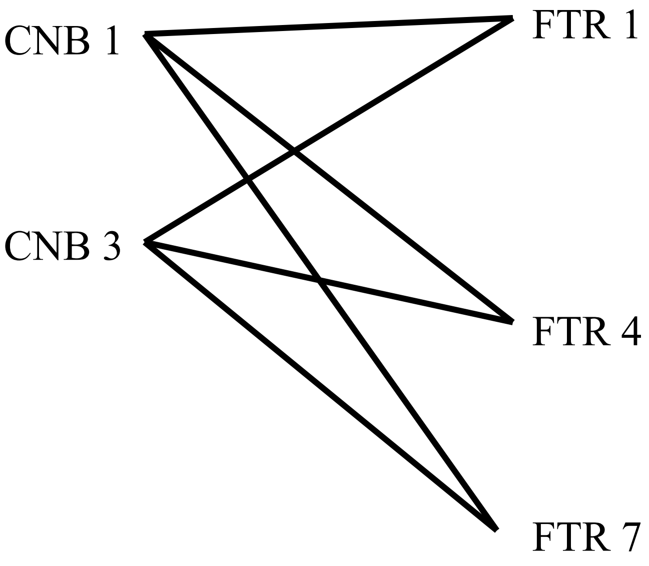
CNB 1
CNB 3
FTR 1
FTR 4
FTR 7

*TOR1 v. FHB1*

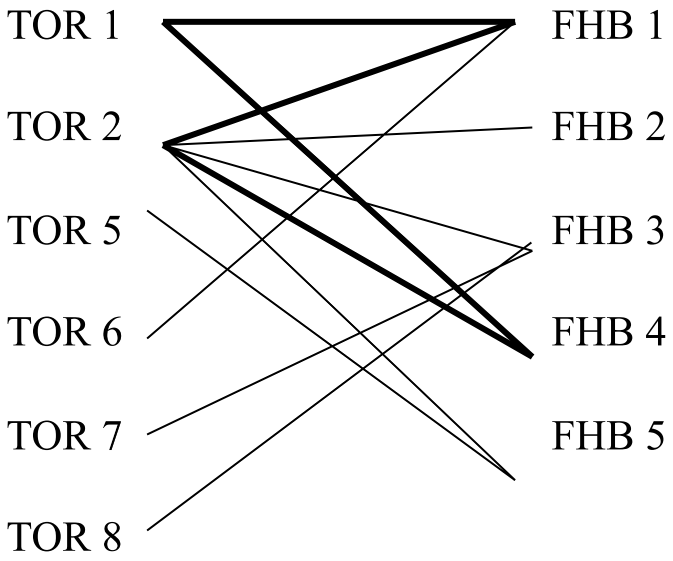

TOR 1
TOR 2
TOR 5
TOR 6
TOR 7
TOR 8
FHB 1
FHB 2
FHB 3
FHB 4
FHB 5


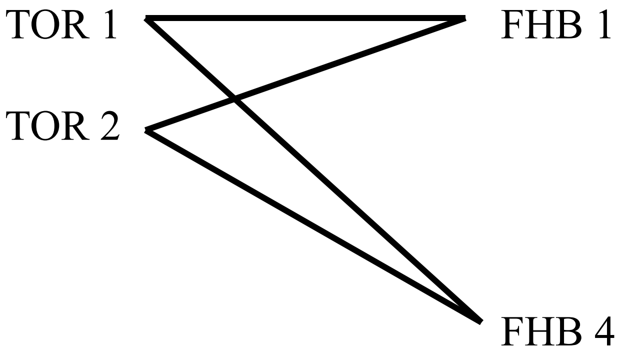

TOR 1
TOR 2
FHB 1
FHB 4

# *TOR1 v. CAP59*

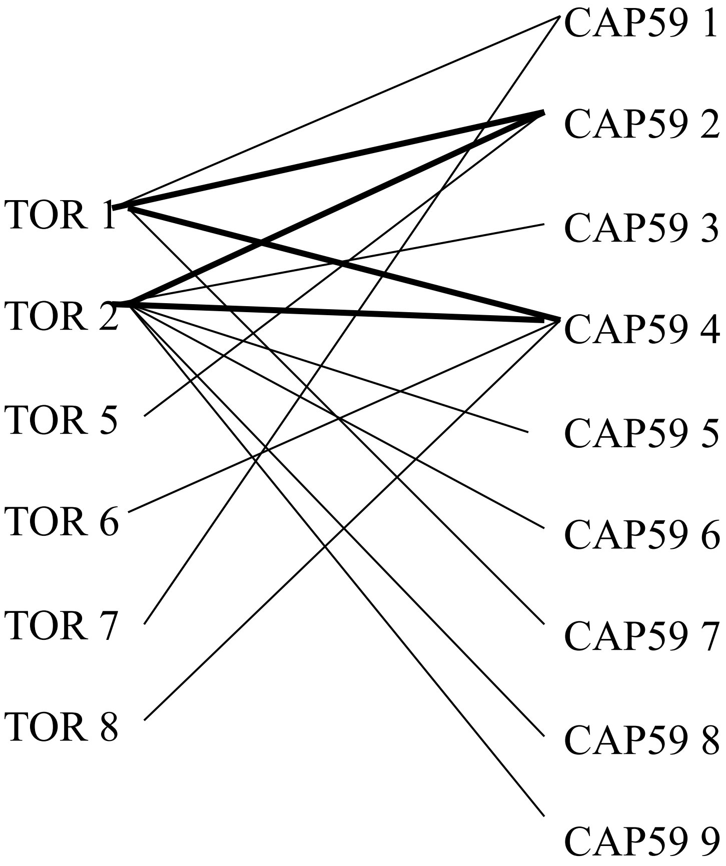

CAP59 1
CAP59 2
TOR 1
CAP59 3
TOR 2
CAP59 4
TOR 5
CAP59 5
TOR 6
CAP59 6
TOR 7
CAP59 7
TOR 8
CAP59 8
CAP59 9


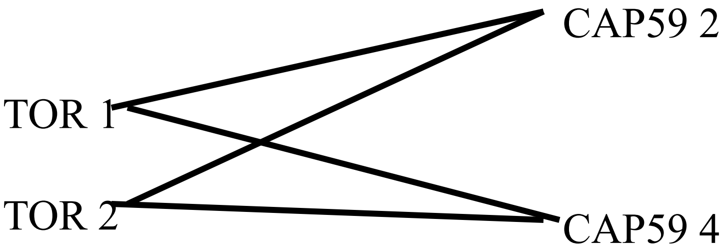

CAP59 2
TOR 1
TOR 2
CAP59 4

# *CRG1 v. FTR1*

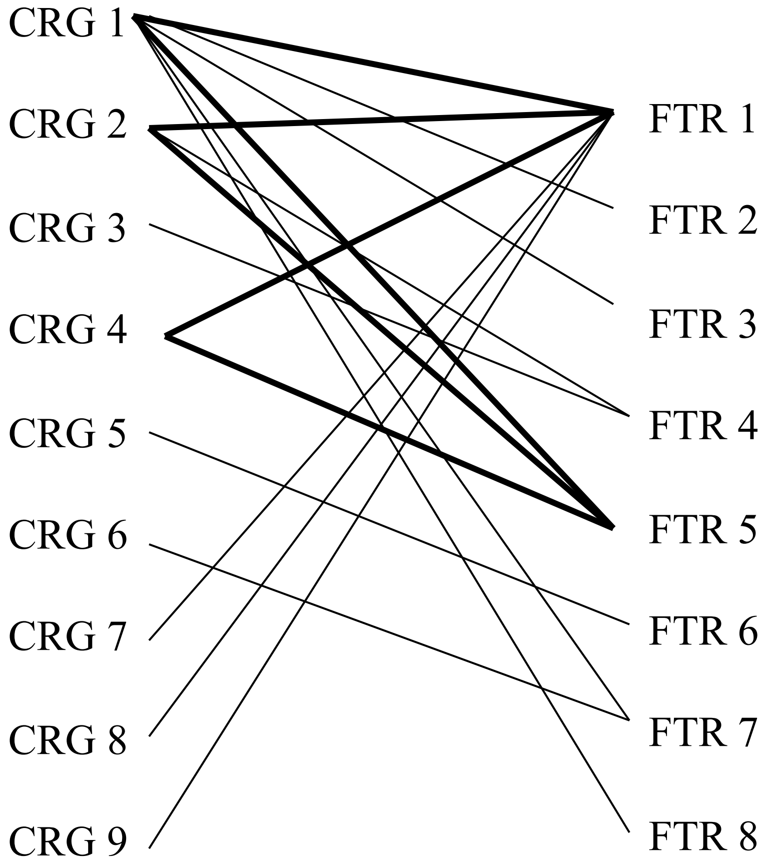
CRG 1
CRG 2
CRG 3
CRG 4
CRG 5
CRG 6
CRG 7
CRG 8
CRG 9
FTR 1
FTR 2
FTR 3
FTR 4
FTR 5
FTR 6
FTR 7
FTR 8

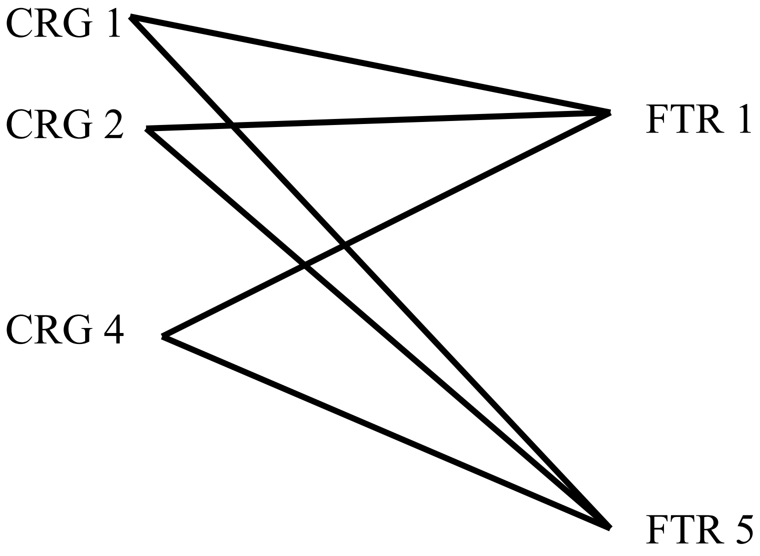
CRG 1
CRG 2
CRG 4
FTR 1
FTR 5

*FHB1 v. CAP59*

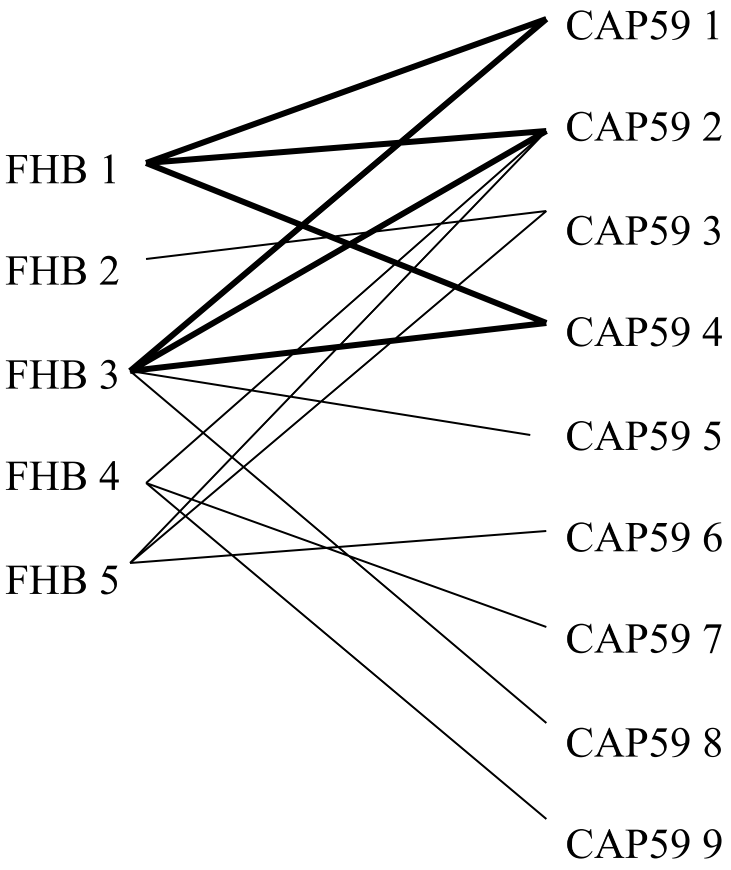
CAP59 1
CAP59 2
FHB 1
FHB 2
CAP59 3
CAP59 4
FHB 3
CAP59 5
FHB 4
CAP59 6
FHB 5
CAP59 7
CAP59 8
CAP59 9

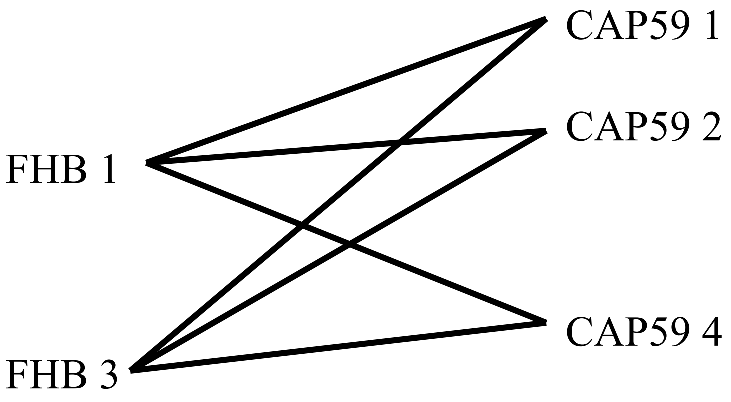
CAP59 1
CAP59 2
FHB 1
CAP59 4
FHB 3

# *FTR1 v. CAP59*

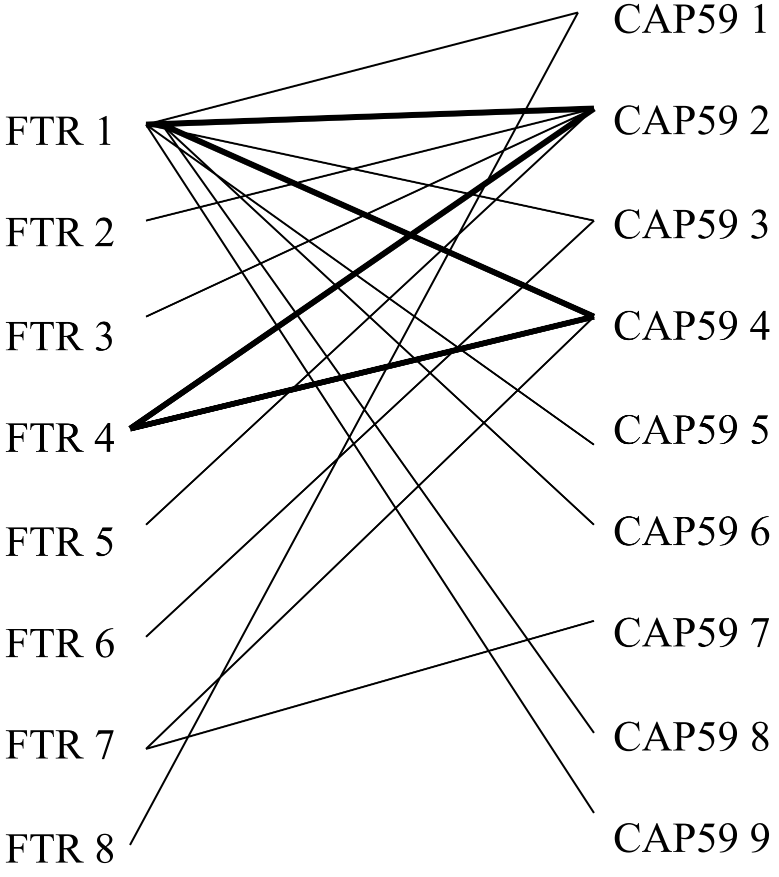
FTR 1
FTR 2
FTR 3
FTR 4
FTR 5
FTR 6
FTR 7
FTR 8
CAP59 1
CAP59 2
CAP59 3
CAP59 4
CAP59 5
CAP59 6
CAP59 7
CAP59 8
CAP59 9

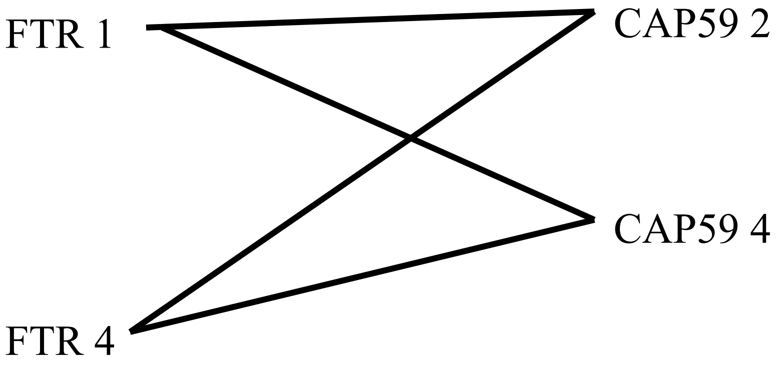
FTR 1
FTR 4
CAP59 2
CAP59 4

| | | | | | | | | | |
|---|---|---|---|---|---|---|---|---|---|
| ICB184 | 23 | - | 15 | 19 | 6 | 4 | 1 | 2 | 5 |
| 2004/335 | 23 | - | 22 | 7 | 6 | 4 | 1 | 2 | 5 |
| ICB183 | 23 | - | 30 | 7 | 21 | 4 | 14 | 18 | 5 |
| 93/980 | 23 | - | 27 | 5 | 22 | 21 | 1 | 19 | 5 |
| ICB182 | 23 | - | 21 | 20 | 6 | 21 | 1 | 2 | 5 |
| 97/170 | 23 | - | 28 | 5 | 21 | 21 | 1 | 2 | 5 |
| ICB179 | 28 | - | 16 | 7 | 6 | 4 | 10 | 16 | 5 |
| ICB97 | 28 | - | 25 | 7 | 24 | 4 | 10 | 24 | 5 |
| 96/1120-1 | 26 | - | 21 | 19 | 21 | 4 | 10 | 16 | 5 |
| 2003/125 | 19 | - | 26 | 7 | 25 | 21 | 1 | 9 | 5 |
| 98/1037-2 | 28 | - | 26 | 7 | 25 | 21 | 1 | 9 | 5 |
